# Modeling the Justinianic Plague: Comparing hypothesized transmission routes

**DOI:** 10.1371/journal.pone.0231256

**Published:** 2020-04-30

**Authors:** Lauren A. White, Lee Mordechai

**Affiliations:** 1 National Socio-Environmental Synthesis Center (SESYNC), Annapolis, Maryland, United States of America; 2 Department of History, Hebrew University of Jerusalem, Jerusalem, Israel; Syracuse University, UNITED STATES

## Abstract

The Justinianic Plague, the first part of the earliest of the three plague pandemics, has minimal historical documentation. Based on the limited primary sources, historians have argued both for and against the "maximalist narrative" of plague, i.e. that the Justinianic Plague had universally devastating effects throughout the Mediterranean region during the sixth century CE. Using primary sources of one of the pandemic’s best documented outbreaks that took place in Constantinople during 542 CE, as well as modern findings on plague etiology and epidemiology, we developed a series of dynamic, compartmental models of disease to explore which, if any, transmission routes of plague are feasible. Using expected parameter values, we find that the bubonic and bubonic-pneumonic transmission routes exceed maximalist mortality estimates and are of shorter detectable duration than described by the primary sources. When accounting for parameter uncertainty, several of the bubonic plague model configurations yielded interquartile estimates consistent with the upper end of maximalist estimates of mortality; however, these models had shorter detectable outbreaks than suggested by the primary sources. The pneumonic transmission routes suggest that by itself, pneumonic plague would not cause significant mortality in the city. However, our global sensitivity analysis shows that predicted disease dynamics vary widely for all hypothesized transmission routes, suggesting that regardless of its effects in Constantinople, the Justinianic Plague would have likely had differential effects across urban areas around the Mediterranean. Our work highlights the uncertainty surrounding the details in the primary sources on the Justinianic Plague and calls into question the likelihood that the Justinianic Plague affected all localities in the same way.

## Introduction

Since the beginning of the 21st century, scholars have paid increasing attention to the series of epidemics that began with the Justinianic Plague, the first part of the earliest plague pandemic (c. 541–750 CE). Historians have compiled catalogs of the known plague references [1–3], that build upon the ancient primary sources, i.e. texts that were written by contemporaries, who were often historians (e.g. Prokopios, Evagrios, John of Ephesos). Even so, the limited details in historical texts have led scholars to question whether the causative agent of Justinianic Plague was truly *Yersinia pestis*, a debate that was only resolved recently through ancient DNA analysis [4–7].

Historians have outlined two broad paradigms of the Justinianic Plague. Maximalists believe that the Justinianic Plague resulted in catastrophic mortality, killing between a quarter and half of the population of the Eastern Roman Empire, or 15–100 million people, over a few years [3,5,8]. This narrative often associates plague with the fall of Rome, the end of Antiquity, and the beginning of the Middle Ages. In contrast, others argue that such estimates of plague mortality are exaggerated [9–12]. Recent work has pointed to a series of proxy measures—ranging from contemporary inscriptions to pollen in lakebed sediments—that show no evidence for a major demographic change in the period [13].

Neither side has attempted to employ mathematical models of disease in their arguments. Instead, maximalist mortality estimates have been based on anecdotal evidence in the primary sources and simple comparisons with the Black Death. However, unlike the Black Death, the Justinianic Plague has little concrete evidence. For example, there are no historical documents for the Justinianic Plague that would allow scholars to reconstruct mortality rates or charts. In fact, the Justinianic Plague features almost no precise, time-course data that could be used to validate an epidemiological model of the type that has been used for the Second and Third Pandemics (e.g. [14–16]).

The complex transmission cycle of plague makes understanding the potential effects of the Justinianic Plague even more challenging. Modern plague, the closest equivalent to historical plague [17], relies on numerous sylvatic rodent reservoirs with periodic incursions into domestic animal and human populations. Bubonic plague in humans may progress to secondary pneumonic plague, which can lead to person-to-person transmission that is independent from the presence of a vector [18,19]. The epidemiological pathway of the Justinianic Plague, however, remains unclear. Although the main assumption in scholarship is that rats and fleas are the primary reservoirs and vectors contributing to spillover in humans, there is very little evidence for enough rats in the premodern Mediterranean to sustain a pandemic (cf. references in [12,20,21]). While pneumonic plague and human ectoparasites (i.e. human lice and fleas), have been suggested as an alternative pathway for the Second Pandemic [16], neither transmission mode is well supported for the Justinianic Plague and both remain conjectural.

The city of Constantinople in 542 represents the best-documented case study of the first pandemic by a large margin. A huge city by contemporary standards, Constantinople was the capital of the Eastern Roman Empire and the permanent place of residence of both the emperor and the imperial government. The historical sources that report on the outbreak refer to its temporal shape, length, and a few anecdotal mortality counts. In parallel, historical research has provided estimates of additional parameters such as the city’s population and general life expectancy in antiquity. Since the primary sources provide almost no concrete supporting evidence about the transmission mode of the Justinianic Plague, we developed a series of dynamic, ordinary differential equation (ODE) models that represent hypothesized transmission routes based on the modern understanding of plague etiology and transmission. We then compared the output of these models to the anecdotal evidence from the primary sources to test hypotheses in contemporary historical research about the transmission route and potential magnitude of this outbreak.

We sought to answer three key questions: (1) given our modern understanding of plague epidemiology, could a plague outbreak in Constantinople have the impact described by the historical primary sources, as argued by the maximalist narrative?; (2) which, if any, hypothesized transmission routes would enable an impact of this magnitude?; and (3) within the context of our sensitivity analysis, to what extent are the results from Constantinople generalizable to other first pandemic outbreaks around the Mediterranean? Although we focus our analysis on the case study of Constantinople in 542 and evaluate the results of our models accordingly, the uncertainty of the model parameters can serve as a proxy for the variety of Mediterranean ecosystems affected by the Justinianic Plague.

## Methods

### Model development

We developed the following ODE models: (1) pneumonic plague (with and without an incubation period for humans); (2) bubonic plague with the traditional rat, flea, and human dynamics (with and without an incubation period for humans, rat growth dynamics, and innate resistance in rats); and (3) bubonic plague that can develop into secondary pneumonic infection and ongoing pneumonic transmission. We compared time course results using expected parameter values and compared outcomes from LHS sampling. We evaluated the models based on the relevant primary sources that plague historians use to study the Justinianic Plague.

#### Initial conditions and parameter estimates

Since historians debate practically all late antique quantitative historical data, parameter values derived from the historical sources remain rough estimates. Our parameter values are therefore assumptions based on historical scholarship. We assumed an initial population size of 500,000 people in Constantinople, based on the estimates of its population during the outbreak [22]. We also assumed that the human population size was approximately constant apart from disease-induced mortality, such that human birth rates were roughly equal to human non-plague related mortality rates and that mean life expectancy was between 20 and 30 years of age [23].

We used recent literature on plague to derive relevant biological parameter values and included possible ranges for exploration with Latin Hypercube Sampling (LHS) (Table 1). We relied on a combination of estimates from empirical infection studies, mechanistic disease models, and models fitted to historical and present plague outbreaks. Where possible, parameter estimates for hosts and vectors are based on the estimates for the black rat (*Rattus rattus)* and the oriental rat flea (*Xenopsylla cheopis*), which are critical actors in plague transmission worldwide; both are commonly associated with historical plague [3,24] and frequently appear in earlier plague studies and models (e.g. [16,25,26]). Expected values were mean values in the traditional sense for normally distributed parameters, but could also be mode values for asymmetrical triangle distributions or simply a point estimate for uniform distributions. When parameter estimates from the literature were highly variable (e.g. estimates of pneumonic plague transmission rate or transmission rate of bubonic plague from fleas to humans), we used a uniform sampling distribution to better assess the effects of parameter uncertainty on model outcomes.

**Table 1:** Expected parameter values derived from the literature.

Parameter	Description	Expected value [Range: min, max]	Distribution	Reference(s)
*r*_*h*_	Human birth rate	1/(25·365) = 0.00011 [1/(30·365) = 0.000091, 1/(20·365) = 0.00013] days^-1^	Uniform	[23]; also, consistent with 0.04 yr^-1^ [26]
*d*_*h*_	Natural human death rate	1/(25·365) = 0.00011 [1/(30·365) = 0.000091, 1/(20·365) = 0.00013] days^-1^	Uniform	[23]; also, consistent with 0.04 yr^-1^ [26]
*β*_*p*_	Pneumonic plague transmission rate in humans	0.08 [0.01, 1] days^-1^	Uniform	0.0734 (SE = 0.00005) [27]; 0.08 [28]; 0.084 [19];0.42–0.48 [16];
*σ*_*p*_^−1^	Duration of pneumonic incubation rate in humans	4.3 [2.5–6.1] days	Normal	[27]; 2–4 days [18]
*γ*_*p*_^−1^	Duration of pneumonic infection period in humans	2.5 [1.3–3.7] days	Normal	[27]
*β*_*r*_	Transmission rate from fleas to rats	1.248 [0–3.67] fleas^-1^ days^-1^	Triangle	0.04–0.14 days^-1^ [16]; 0–3.67 days^-1^ *calculated from percent per flea transmission efficiency/days post infection at 23°C [29]
*α*	Flea searching efficiency	3/*S*_*r*_(*t* = 0) [0.39 < *αK*_*r*_< 20] rats^-1^	Uniform	[26,30]
*r*_*r*_	Reproductive rate in rats	0.014 [0.011, 0.016] rats days^-1^	Uniform	5/365 days [25,26]
*K*_*r*_	Carrying capacity of rats	*N*_*r*_(*t* = 0) [0.5*N*_*r*_(*t* = 0), 1.5*N*_*r*_(*t* = 0)] rats	Uniform	Varied in conjunction with sensitivity analysis on initial condition (N_r_)
*d*_*r*_	Natural death rate in rats	0.2 year^-1^ /365 = 0.00055 [0.1/365 = 0.00027, 0.3/365 = 0.00082] days^-1^	Uniform	[25,26]
*p*_*r*_	Probability of rats inheriting resistance	0.65 [0.4–0.9]	Uniform	0.5 [31]; 0.5–0.9 [32]; 0.4–0.8 [33]
*γ*_*r*_^−1^	Duration of bubonic plague infectious period in rats	5.15 [4.71–5.59] days	Normal	Based on low-moderate infection doses in rats outside of plague endemic areas in Madagascar [32]
*g*_*r*_	Probability of rats recovering from bubonic plague	0.06 [0.0–0.37]	Triangle	“24–37% of rats surviving a low- dose infection . . .compared with only 0–6% at high dose” [32]
*r*_*f*_	Growth rate of fleas	0.0084 [0.0084, 20/365 = 0.055] fleas/day	Uniform	[16,25,26]
*K*_*f*_	Flea carrying capacity per rat	6 [3.29, 11.17] fleas	Normal	[16,25,26]
*d*_*f*_^−1^	Flea lifespan	5 [1, 11.66] days	Triangle	[16,34]
*β*_*b*_	Transmission rate for bubonic plague from rat fleas to humans	0.19 [0.01, 1] days^-1^	Uniform	0.18–0.20 [16]
*σ*_*b*_^−1^	Duration of bubonic plague incubation period in humans	4 [2, 6] days	Triangle	[35]; 4.3 days [36]
*γ*_*b*_^−1^	Duration of bubonic plague infectious period in humans	10 [3, 10] days	Triangle	3.4 days [36]; 4–10 days [37]; 10 days [16];
*g*_*h*_	Probability of humans recovering from bubonic plague	0.34 [0.30, 0.40]	Triangle	0.40 [16]; “Among 511 plague cases occurring before 1942 (pre-antibiotics) with outcome information, 336 (66%) were fatal” [38]
*p*	Probability of human bubonic plague developing into secondary pneumonic plague	0.10 [0, 0.15]	Triangle	3% [36]; <5% [18]; ~10% [39]; 8–10% [37]; ~12% [40]; 5–15% [41]

Here we include the parameter, its description, expected value, range tested during the global sensitivity analysis (LHS-PRCC), and type of distribution used for sampling.

#### Pneumonic plague (humans only)

Pneumonic plague consists of human-to-human airborne infection. For pneumonic plague, we considered transmission solely within the human population and investigated the consequences of demographic rates and an incubation period on the predicted epidemic dynamics. Without treatment, pneumonic plague has a fatality rate approaching 100%. Therefore, in accordance with previous pneumonic plague models, we did not include a recovery class [16,27]. We assumed frequency-dependent transmission since close contact with an infected individual drives pneumonic plague transmission, requiring exposure to aerosolized bacteria [18,42]. Beginning with the simplest case of a Susceptible-Infected-Removed (SIR) compartmental model, the total number of humans (*N*_*h*_) is given by the equation:

$$N_{h}(t)=S_{h}(t)+I_{h}(t)$$

The change in the number of susceptible (*S*_*h*_), infected (*I*_*h*_), and dead (*D*_*h*_) humans are given by the equations:

$$\frac{dS_{h}}{dt}=b_{h}S_{h}-\beta_{p}S_{h}\frac{I_{h}}{N_{h}}-d_{h}S_{h}$$

$$\frac{dI_{h}}{dt}=\beta_{p}S_{h}\frac{I_{h}}{N_{h}}-\gamma_{p}I_{h}$$

$$\frac{dD_{h}}{dt}=\gamma_{p}I_{h}$$

Extending this to an SEIR framework by adding in an incubation period (*σ*_*p*_^−1^), the total number of humans becomes: *N*_*h*_(*t*) = *S*_*h*_(*t*)+*E*_*h*_(*t*)+*I*_*h*_(*t*)

In addition, the rates of change of the number of susceptible (*S*_*h*_), exposed (*E*_*h*_), infected (*I*_*h*_), and dead (*D*_*h*_) humans are given by the equations:

$$\frac{dS_{h}}{dt}=b_{h}S_{h}-\beta_{p}S_{h}\frac{I_{h}}{N_{h}}-d_{h}S_{h}$$

$$\frac{dE_{h}}{dt}=\beta_{p}S_{h}\frac{I_{h}}{N_{h}}-\sigma_{p}E_{h}\frac{dI_{h}}{dt}=\sigma_{p}E_{h}-\gamma_{p}I_{h}$$

$$\frac{dD_{h}}{dt}=\gamma_{p}I_{h}$$

#### Bubonic plague (humans, fleas, and rats)

The most common plague model suggested in the scholarly literature is a transmission cycle maintained through rodents and fleas. Bubonic plague occurs in wild rodent populations and occasionally spills over to humans through contact with wild or peridomestic rodents [43]. The most common rodent associated with plague spillover to humans is the black rat (*Rattus rattus)*. Third Pandemic comparatives from 20th century India have suggested that high mortality among rats precedes human mortality, as fleas eventually turn to feed upon humans after their preferred rat hosts have died [44].

To model the bubonic plague transmission route, we considered populations of rats (the primary host), fleas (their vectors), and humans. For rats, dynamics are given in the form of a Susceptible-Infected-Recovered (SIR) set of equations where the total number of rats (*N*_*r*_) is given by:

$$N_{r}(t)=S_{r}(t)+I_{r}(t)+R_{r}(t)$$

Ignoring births and deaths in the rat population, the number of susceptible (*S*_*r*_), infected (*I*_*r*_), recovered (*R*_*r*_), and dead (*D*_*r*_) rats is given by:

$$\frac{dS_{r}}{dt}=-\beta_{r}S_{r}F(1-e^{-\alpha N_{r}})/N_{r}$$

$$\frac{dI_{r}}{dt}=\beta_{r}S_{r}F(1-e^{-\alpha N_{r}})/N_{r}-\gamma_{r}I_{r}$$

$$\frac{dR_{r}}{dt}=g_{r}\gamma_{r}I_{r}$$

$$\frac{dD_{r}}{dt}={(1-g}_{r})\gamma_{r}I_{r}$$

Here *β*_*r*_ describes the transmission rate from rats to fleas, *α* describes the flea searching efficiency, *γ*_*r*_^−1^ describes the bubonic infectious period in rats, and *g*_*r*_ describes the probability that rats survive bubonic plague infection. We assume that the encounter rate between rats and fleas is represented by a random search process of fleas within a limited area, which is modulated by the number of available rats (*N*_*r*_) and the searching efficiency of the fleas (*α*) [25,26]. The formulation for this per capita searching efficiency (*α*) comes from Nicholson & Bailey’s description of host-parasitoid dynamics [45] and has been used extensively in the plague modeling literature to describe flea and rat encounter rates [16,25,26]. The model accounts for the number of expected fleas per rat (*H*) and the number of free infectious fleas (*F*) in the environment that may encounter human hosts.

$$\frac{dH}{dt}=r_{f}H(1-H/K_{f})$$

$$\frac{dF}{dt}=(1-g_{r})\gamma_{r}I_{r}H-d_{f}F$$

Previous modeling studies have identified the sensitivity of this model to the interaction between carrying capacity (*K*_*r*_) and flea searching efficiency (*α*) [25,26]. Recent studies of endemic plague foci suggest that some rat populations are resistant to plague [31,32]. Therefore, we also modelled the case where rats grow at a rate modulated by their carrying capacity (*K*_*r*_), die at a certain rate (*d*_*r*_), and have a probability (*p*_*r*_) of being born resistant to plague. The series of equations for the rat population then becomes:

$$\frac{dS_{r}}{dt}=-r_{r}S_{r}(1-N_{r}/K_{r})+r_{r}R_{r}(1-p_{r})-d_{r}S_{r}-\beta_{r}S_{r}F(1-e^{-\alpha N_{r}})/N_{r}$$

$$\frac{dI_{r}}{dt}=\beta_{r}S_{r}F(1-e^{-\alpha N_{r}})/N_{r}-\gamma_{r}I_{r}$$

$$\frac{dR_{r}}{dt}={r_{r}R_{r}(p_{r}-N_{r}/K_{r})+g}_{r}\gamma_{r}I_{r}-d_{r}S_{r}$$

$$\frac{dD_{r}}{dt}={(1-g}_{r})\gamma_{r}I_{r}$$

For the bubonic SIR model, the total number of humans (*N*_*h*_) is given by: *N*_*h*_(*t*) = *S*_*h*_(*t*)+*I*_*h*_(*t*)+*R*_*h*_(*t*)

In addition, the number of susceptible (*S*_*h*_), infected (*I*_*h*_), recovered (*R*_*h*_), and dead (*D*_*h*_) humans are given by the equations:

$$\frac{dS_{h}}{dt}=-\beta_{b}\frac{S_{h}}{N_{h}}F(e^{-\alpha N_{r}})+b_{h}(S_{h}+I_{h})-d_{h}S_{h}$$

$$\frac{dI_{h}}{dt}=\beta_{b}\frac{S_{h}}{N_{h}}F(e^{-\alpha N_{r}})-\gamma_{b}I_{h}$$

$$\frac{dR_{h}}{dt}=g_{h}\gamma_{b}I_{h}-d_{h}R_{h}$$

$$\frac{dD_{h}}{dt}=(1-g_{h})\gamma_{b}I_{h}$$

For the bubonic SEIR model, including an incubation period, the total number of humans is represented by the equation: *N*_*h*_(*t*) = *S*_*h*_(*t*)+*E*_*h*_(*t*)+*I*_*h*_(*t*)+*R*_*h*_(*t*)

The number of susceptible (*S*_*h*_), exposed (*E*_*h*_), infected (*I*_*h*_), recovered (*R*_*h*_), and dead (*D*_*h*_) humans are given by the equations:

$$\frac{dS_{h}}{dt}=-\beta_{b}\frac{S_{h}}{N_{h}}F(e^{-\alpha N_{r}})+b_{h}(S_{h}+I_{h})-d_{h}S_{h}$$

$$\frac{dE_{h}}{dt}=\beta_{b}\frac{S_{h}}{N_{h}}F(e^{-\alpha N_{r}})-\sigma_{b}E_{h}$$

$$\frac{dI_{h}}{dt}=\sigma_{b}E_{h}-\gamma_{b}I_{h}$$

$$\frac{dR_{h}}{dt}=g_{h}\gamma_{b}I_{h}-d_{h}R_{h}$$

$$\frac{dD_{h}}{dt}=(1-g_{h})\gamma_{b}I_{h}$$

#### Bubonic/Pneumonic transmission

In the final scenario, we model a pneumonic plague outbreak that emerges and is transmitted separately from a bubonic plague transmission cycle. Humans infected with rat-and-flea transmitted bubonic plague can develop secondary pneumonic plague, potentially initiating a chain of human-to-human pneumonic plague transmission [36,37]. In this case, the total number of humans is described by the equation:

$$N_{h}(t)=S_{h}(t)+E_{b}(t)+E_{p}(t)+I_{b}(t)+I_{p}(t)+R_{h}(t)$$

In addition to the susceptible, recovered, and dead classes appearing in prior models, individuals may experience exposure to bubonic (*E*_*b*_) or pneumonic plague (*E*_*b*_) and proceed to infection with bubonic (*I*_*b*_) or pneumonic plague (*I*_*p*_):

$$\frac{dS_{h}}{dt}=-\beta_{b}\frac{S_{h}}{N_{h}}F(e^{-\alpha N_{r}})-\beta_{p}S_{h}\frac{I_{h}}{N_{h}}+b_{h}(S_{h}+I_{h})-d_{h}S_{h}$$

$$\frac{dE_{b}}{dt}=\beta_{b}\frac{S_{h}}{N_{h}}F(e^{-\alpha N_{r}})-\sigma_{b}E_{h}$$

$$\frac{dE_{p}}{dt}=\beta_{p}S_{h}\frac{I_{h}}{N_{h}}-\sigma_{p}E_{p}$$

$$\frac{dI_{b}}{dt}=\sigma_{b}E_{b}-\gamma_{b}I_{b}$$

$$\frac{dI_{p}}{dt}=\sigma_{p}E_{p}+p\gamma_{b}I_{b}-\gamma_{p}I_{p}$$

$$\frac{dR_{h}}{dt}=g_{h}\gamma_{b}I_{b}-d_{h}R_{h}$$

$$\frac{dD_{h}}{dt}=(1-p-g_{h})\gamma_{b}I_{h}+\gamma_{p}I_{p}$$

Those with bubonic plague may transition to pneumonic plague with a probability, *p*, recover with a probability, *g*_*h*_, or die. As outlined in the pneumonic models above, those infected with pneumonic plague are assumed to have 100% fatality.

### Time course results and sensitivity analysis

**Fig 1:**
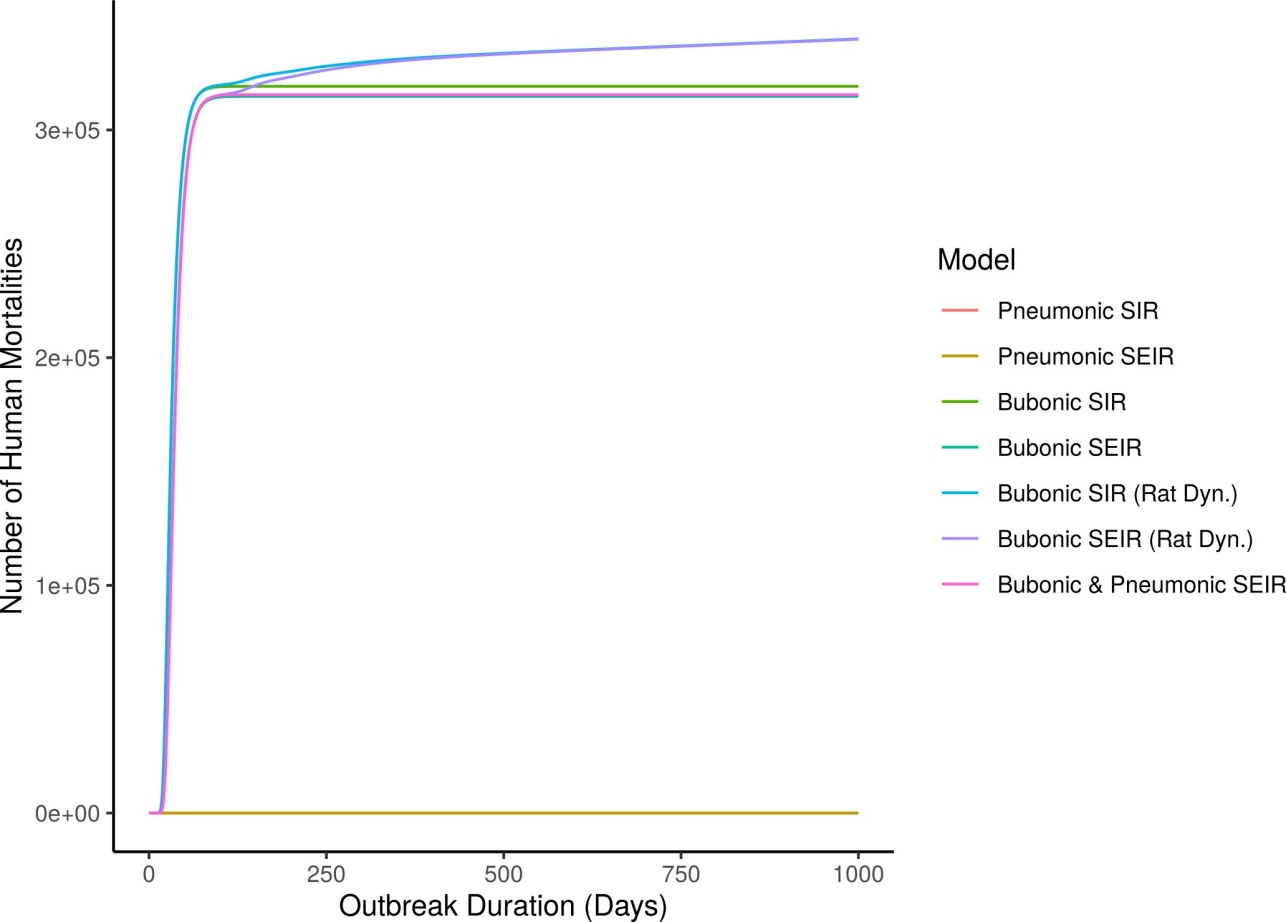
Time course results of different transmission mode models with rat to human ratio of 1:1. Produced using expected transmission values from Table 1. Initial conditions: number of susceptible humans, *S*_*h*_(*t* = 0) = 500,000, number of susceptible rats, *S*_*r*_(*t* = 0) = 499,999, and number of infected rats, *I*_*r*_(*t* = 0) = 1.

We solved models numerically using the *ode* function from the *deSolve* package in R [46]. For time course results of bubonic models, we explored initial conditions with different ratios of rats-to-people: 1:2, 1:1, 2:1 (Fig 1 and S1 and S2 Figs). For all models, we conducted a global sensitivity analysis using Latin Hypercube Sampling with Partial Ranked Correlation Coefficients (LHS-PRCC) to evaluate the relative importance of each parameter to the model outcome by detecting monotonic relationships between parameters and outputs while accounting for the effects of all other parameters [47]. LHS is a sampling scheme that divides each parameter space into N fractions and samples only once from each of those fractions. Therefore, LHS is a more efficient sampling method than general Monte Carlo Sampling [48]. The minimum required sample size (*N*) for LHS is *N*≥*K*+1 or *N*≥4/3∙*K* where *K* is the number of parameters included in the LHS [49]. The models here range in complexity from 4 to 17 parameters per model. We created 100 subdivisions per parameter using the *lhs* package in R (version 3.5.3) to generate the LHS framework [50].

We conducted PRCC on two outcomes: total mortality and detectable outbreak duration. We calculated detectable outbreak duration as the number of non-consecutive days where the mortality rate exceeded 100 deaths per day. To account for multiple comparisons, we used a Bonferroni altered/corrected p-value (i.e. *p* = 0.05 divided by the number of model parameters) for calculating confidence intervals. We used 500 bootstraps replicates per sensitivity index using the *pcc* function from the *sensitivity* package when calculating the PRCC values [51]. All code is deposited at Zenodo: https://doi.org/10.5281/zenodo.3728203.

### Model evaluation: Evidence from the primary sources

Although numbers in premodern historical texts are notoriously unreliable, plague maximalists and the majority of broader scholarship have generally accepted the accounts and the numbers they provide [4,52]. Since we attempted to test for the feasibility of the maximalist narrative of the Justinianic Plague, we accepted these numbers at face value for comparison in our analysis.

The historian John of Ephesus, one eyewitness of the first outbreak, estimated that 300,000 people in Constantinople died during the first outbreak in the city, claiming that officials stopped counting when they reached 230,000 [53]. Maximalists also cite Prokopios as evidence that plague killed half the empire’s population, although the relevant statement is often presented without context [4,12,54]. Regardless, for the purposes of this paper we estimated the mortality of the first outbreak in Constantinople as about half the city’s population (i.e. 250,000 people), following the higher end of the range of the maximalist interpretation. Prokopios, the most important eyewitness for the Justinianic Plague, supplies additional information when he asserts that [55]:

*“Now the disease in Byzantium* [i.e. Constantinople] *ran a course of four months*, *and its greatest virulence lasted about three*. *And at first the deaths were a little more than the normal*, *then the mortality rose still higher*, *and afterwards the tale of dead reached five thousand each day*, *and again it even came to ten thousand and still more than that*.*”*

As the quote reveals, it is impossible to extract precise numbers from Prokopios’ account. Historians debate how to treat such vague and suspiciously round estimates, with some choosing to accept these estimates at face value and others remaining more critical towards them (compare [3,12]). Although the contemporary Roman Empire was a complex bureaucratic society, how Prokopios or other sources would have known these numbers remains unclear. It seems unlikely that authorities counted corpses when, as the sources say, people stayed off the streets, corpses remained unburied, and the government shut down. Notably, other ancient historians refer to reasonable methodologies–for example, after an earthquake in Antioch in the late sixth century, the historian Evagrios explains his estimate of 60,000 deaths by the number of people who stopped coming to reclaim their government-supplied free bread [56].

Converting Prokopios’ text into quantitative metrics, we arbitrarily assumed that plague would be noticeable at about 100 deaths/day above the baseline mortality rate in the city over all four months, and that the three months with higher mortality should have at least 250 additional deaths/day. We further use Prokopios’ information about the peak of the mortality rate during the Constantinople outbreak—namely, his claim that mortality reached 5,000 deaths/day and eventually 10,000 deaths/day. We chose to follow Prokopios as his relatively detailed account serves as the basis of modern scholarship on plague and is perceived by scholars to be a more reliable source that John of Ephesus, who also provides mortality estimates of up to 16,000 deaths/day and the death of 99.9% of the population [53,57].

## Results

### Model outcomes with expected parameter values

Using expected parameter values, the outbreaks predicted by the pneumonic (SIR and SEIR) models did not spread successfully and did not exhibit mortality rates exceeding 100 deaths per day (Fig 1). The results of all five bubonic and bubonic-pneumonic models were relatively similar and substantially different from the pneumonic models, with estimates of c. 315,000–340,000 mortalities (63–68% of the city’s population). The two bubonic plague model variants with rat reproduction and resistance predicted the highest mortality among these models (Fig 1). The mortality in all five models followed a similar trajectory over time. Noticeable mortality of more than 100 deaths/day began between days 13–15, lasted between 70–76 days, and ended between days 83–91 (Table 2). The more significant mortality of more than 250 deaths/day began between days 19–22, continued for between 60–64 days, and ended between days 74–80 (Table 2). The bubonic SEIR model with rat growth and resistance predicted an additional period of 27 days of high mortality over a month after the previous phase of increased mortality ended (for a total of 103 days with increased mortality). The bubonic SIR model with rat growth and resistance predicted a similar trend, albeit below the 100 deaths/day limit (Table 2).

**Table 2:** Summary of model output for each model type with rat to human ratio of 1:1.

Model	Detectable outbreak duration (deaths/day)		Maximum mortality rate/day	Total mortality (humans)
	> 100	> 250		
Pneumonic SIR	0	0	0.34	1.25
Pneumonic SEIR	0	0	0.33	1.25
Bubonic SIR	70	60	15152	319170
Bubonic SEIR	74	63	12918	314772
Bubonic SIR (Rat dynamics)	72	61	15116	339989
Bubonic SEIR (Rat dynamics)	103 (76 consecutive days, 27 additional days after a break of 39 days)	64	12891	339848
Bubonic/Pneumonic SEIR	74	64	12778	315489

Outcomes include detectable outbreak duration, maximum mortality rate per day, and total mortality. Initial conditions: Number of susceptible humans, *S*_*h*_(*t* = 0) = 500,000, number of susceptible rats, *S*_*r*_(*t* = 0) = 499,999, and number of infected rats, *I*_*r*_(*t* = 0) = 1.

These findings were relatively robust to changes in the initial conditions of the rat to human ratio for the bubonic models (S1 and S2 Figs). With a 1:2 ratio of rats to humans, total mortality decreased to approximately 250,000–257,000 deaths for bubonic SIR, bubonic SEIR, and bubonic/pneumonic SEIR models (S1 Fig). Bubonic SIR and SEIR models with rat growth and resistance still predicted roughly 330,000 deaths. However, these outbreaks took substantially longer (374 and 405 days at greater than 100 deaths per day respectively) (S1 Table). A 2:1 ratio of rats to humans increased the convergence of all of the bubonic models and the bubonic/pneumonic model (S2 Fig). These models predicted human fatalities ranging from 329,000 to 342,000 deaths with 67–71 days exceeding 100 mortalities per day (S2 Table).

### Sensitivity analysis: Model outcome variability and parameter influence

**Fig 2:**
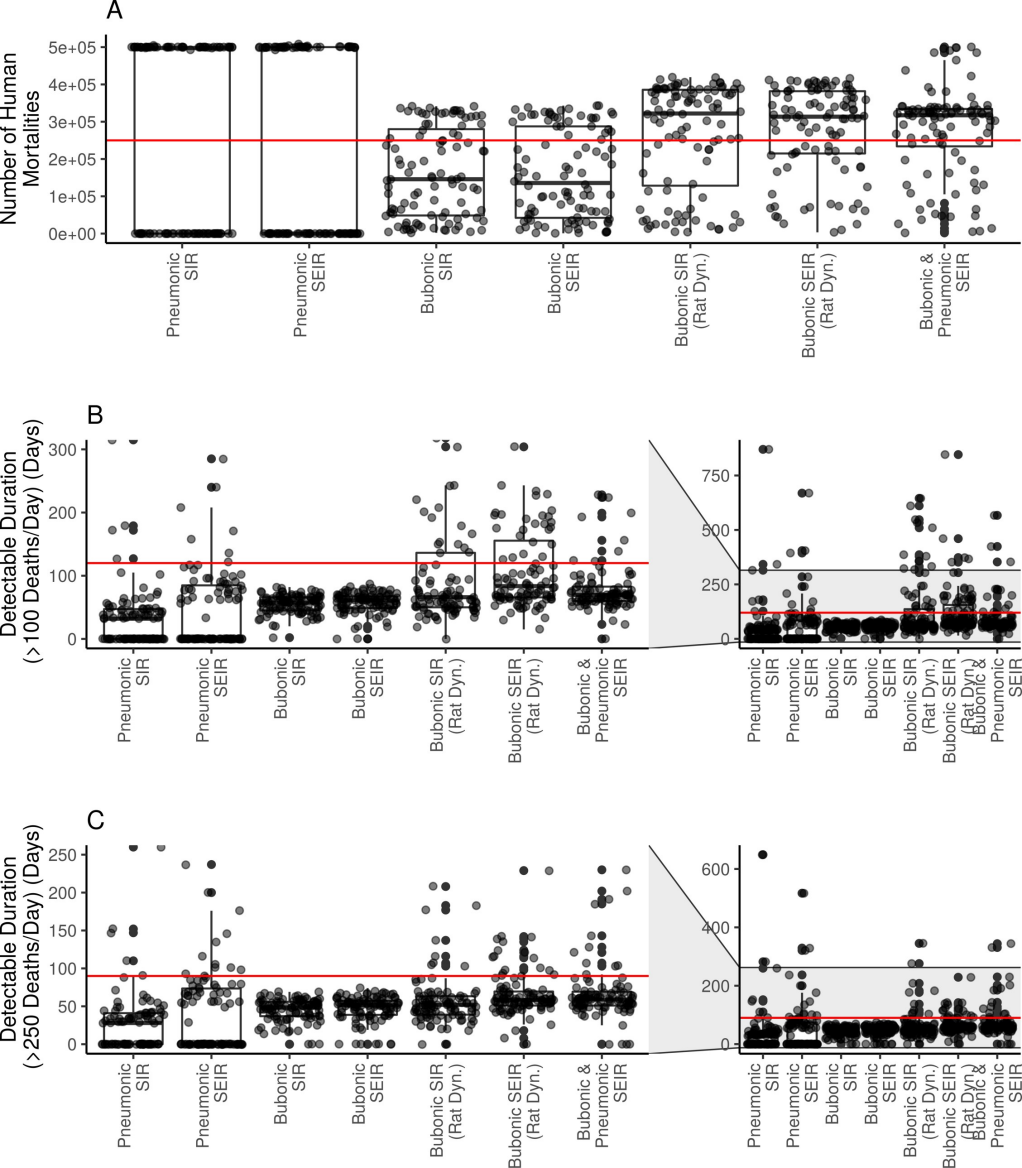
Box and whisker plot showing results of uniform LHS sampling. (A) number of human mortalities; (B) detectable outbreak duration (>100 deaths per day, non-consecutive) with inset including outliers (up to 5000 days); and (C) detectable outbreak duration (>250 deaths per day, non-consecutive) with inset including outliers (up to 5000 days). Red lines depict estimated comparison values from historical primary source accounts: (A) 250,000 mortalities; (B) 4 months or 120 days of mortality exceeding 100 deaths per day; and (C) 3 months or 90 days of mortality exceeding 250 deaths per day.

Based on the LHS sampling results, the estimated mortality of 250,000 deaths from the primary sources falls within the interquartile range for all models (Fig 2A). The median outbreak size is higher for the bubonic SIR and SEIR models with rat dynamics, and the bubonic/pneumonic SEIR model (Fig 2A). The median outbreak size is lower for the bubonic SIR and SEIR models and both pneumonic plague models (Fig 2A). Only one of the models contains the detectable outbreak duration of about 120 days (>100 deaths/day) within their interquartile range: bubonic SIR with rat dynamics (Fig 2B). For the remaining models, the median outbreak size and interquartile range is considerably less than the 120 days reported by the primary sources. None of the models contain the detectable outbreak duration of 90 days (>250 deaths/day) within their interquartile range. These results were robust to the more conservative approach of using uniform sampling distributions for all parameters, although the interquartile range of the detectable outbreak period increased to include the primary source estimates for both pneumonic plague models (S3 Fig).

For the pneumonic SIR and SEIR models, pneumonic transmission rate (𝛽_p_) was positively correlated with outbreak size and detectable duration, and pneumonic plague infectious period (𝛾_p_^-1^) was correlated with decreased outbreak size and detectable duration (S4 Fig). For the bubonic SIR and SEIR models with and without rat dynamics, flea searching efficiency (*α*), flea death rate (*d*_*f*_), and rat recovery probability (*g*_*r*_) were generally all negatively correlated with total mortality (S5 Fig, panels A & C) and duration (S5 Fig, panels B & D). For the bubonic SIR and SEIR models with rat dynamics, transmission rate from fleas to humans (𝛽_b_) also correlated positively with total mortality (S6 Fig).

For the bubonic/pneumonic SIR model, flea searching efficiency (*α*), longer infectious periods of pneumonic plague in humans (𝛾_p_^-1^), and a shorter pneumonic plague incubation period (𝜎_p_^-1^) were correlated with a higher total human mortality (S7A Fig). Flea searching efficiency (*α*) was positively correlated with outbreak duration, while a decreased bubonic incubation period in humans (𝜎_b_^-1^) was correlated with longer detectable outbreaks (S7B Fig). These parameters generally remained significant with the more conservative sampling approach using only uniform parameter distributions (S1 Appendix). A full description of LHS PRCC results and individual correlation plots for each parameter and outcome combination are available in the S1 and S2 Appendices.

## Discussion

Previous retrospective modeling efforts for the Second and Third Pandemics have used precise outbreak data to statistically estimate unknown transmission parameters (e.g. [27]) or to compare possible transmission routes (e.g. [16]). In the absence of such detailed data for the first pandemic in Constantinople in 542, we relied on the testimony of primary sources that gave us rough estimates of total mortality and observed epidemic duration. We then developed a series of compartmental models to test the possible hypothesized transmission routes for the Justinianic Plague with parameters formed from our modern understanding of plague etiology.

### Pneumonic plague

Although the pneumonic plague transmission route remains attractive in some historical literature because of the higher case mortality associated with it, the primary sources for the Justinianic Plague preserve little evidence for it. Very few sources report symptoms that are consistent with pneumonic plague. Moreover, since the buboes—that occur in bubonic but not pneumonic plague—are the most common symptoms reported by far, scholars tend to diagnose late antique plague based on outbreaks during which the sources refer to buboes. It is nearly impossible to identify late antique pneumonic plague and differentiate it from any other epidemic mentioned in the sources.

More broadly, there is little historical evidence for large-scale epidemics of pneumonic plague: the very high mortality rate of pneumonic plague and its relatively brief incubation period tend to kill humans too quickly. Moreover, pneumonic plague outbreaks require close contact between humans and specific environmental conditions. There were two large outbreaks of pneumonic plague in East Asia in the early 20th century that killed tens of thousands of people, but all evidence suggests that these required exceptional cultural and environmental contexts (e.g. [18]).

Using expected parameter values, both our pneumonic plague models (SIR and SEIR) failed to replicate the historical Justinianic Plague for Constantinople. Neither model predicted successful outbreaks (Fig 1), which is not consistent with current maximalist estimates. In addition, neither model yielded periods where disease-related mortality exceeded 100 deaths per day, compared to Prokopios’ account that described visible mortality in the city over four months. Similarly, the highest daily mortality counts in both cases were also considerably lower than the 10,000 claimed by Prokopios. With LHS sampling, the maximalist estimate of ~250,000 deaths falls within the interquartile range of both the pneumonic SIR and SEIR models (Fig 2A). However, the model mortalities were largely dichotomous: either failing to foster onward transmission or decimating the entire population. The median mortalities for both model formulations were substantially lower than primary source descriptions (Fig 2A), offering evidence against the idea that pneumonic plague could spread consistently with high mortality through late antique Mediterranean populations.

### Bubonic plague

Only limited historical evidence supports the bubonic mode of transmission. Comparisons to the Third Pandemic suggest that the number of rats required to support a major epidemic among humans would be very high, as the ratio of infected rats to total rats appears to have been low in most cases. For example in early 20^th^ century Bombay, perhaps the worst case of urban plague during the Third Pandemic, less than 3% of 500,000 rats caught had plague [44]. Similarly low ratios of infected rats to total rats commonly appeared in later surveys as well [58]. A substantial rat population is therefore required to maintain the outbreak among humans. Rat to human ratios are often estimated at 1:1 in an urban environment [16,44].

However, with the exception of one vague reference in a historical text (to generic “mice” in a list of animals who died in the plague), none of the surviving late antique accounts of plague mentions rats. This differs significantly from the Third Pandemic evidence that suggests that preceding rat mortality was very noticeable [44]. There has also been very little archaeological evidence of late antique rat remains in the many hundreds of archaeological excavations throughout the Mediterranean [20,59]. The total number of rat bones found in the Eastern Mediterranean—where we have the most textual evidence for plague—is in the low dozens over centuries. Rat bones are admittedly quite difficult to find in archaeological excavations, yet scholars have been searching for them for over a century and have compiled catalogs of them for over four decades (e.g. [60]).

Although our results for bubonic plague are closer to those of the maximalist interpretation, they ultimately failed to replicate the historical descriptions of the Justinianic Plague outbreak in Constantinople. With expected parameter values, the total number of deaths reached around 315,000–340,000 depending on model construction, which exceeds even the highest current maximalist estimates for Justinianic Plague mortality (Fig 1). At 15,000 to 19,000, the maximum deaths per day exceeded Prokopios’ account of 10,000 (Table 2). In contrast, the total length of increased mortality in most models is shorter than Prokopios’ account with 70–76 days of mortality exceeding 100 deaths per day (Table 2). Although the SEIR model with rat growth and resistance resulted in more days of noticeable mortality (103 in total), these days are split between two periods—a catastrophic period of high mortality, followed by a month-long lull with a subsequent second month-long increase in observable mortality. This unexpected pattern does not appear to conform to the primary source evidence.

For LHS sampling, all bubonic plague model variants contained the mortality estimate of 250,000 deaths within their interquartile ranges (Fig 2A). However, with the exception of the bubonic SIR model with rat dynamics, none of the models contained the detectable outbreak duration of 120 days within their interquartile range (Fig 2B). These results similarly do not conform to the current scholarly understanding of the Justinianic Plague.

### Combined bubonic-pneumonic plague

Our third model combined different transmission routes, coupling the higher mortality of pneumonic plague with the increased persistence of the bubonic rat-flea model, allowing for continuous bubonic infections that potentially transform into pneumonic plague. With expected parameter values, the results of this model were consistent with those of the bubonic models (Fig 1). However, overall mortality reached c. 316,000 (~63% of the population), higher than the current maximalist estimates (Table 2). The maximum death count per day was c. 12,800, i.e. higher than Prokopios’ estimates, while the length of observable increased mortality was very close to those of the bubonic model configurations (i.e. 89 days) (Table 2). Looking at the LHS results, the maximalist estimate of 250,000 deaths is contained within the interquartile range, but remains lower than the median outcome (Fig 2A). Similarly, the interquartile estimate of detectable outbreak duration is shorter than Prokopios’ account of 120 days (Fig 2B).

### Parameter importance and epistemic uncertainty

We conducted a global sensitivity analysis to assess the relative importance of parameters in dictating model outcomes and to identify epistemic uncertainty for model parameters with limited empirical backing. Several parameters with high epistemic uncertainty were consistently important across different model configurations. For example, for both the pneumonic SIR and SEIR models, pneumonic transmission rate (𝛽_p_) was positively correlated with outbreak size (S4 Fig). Fitted estimates in the literature for contemporary outbreaks range from 0.07–0.46 [16,19,27,28]. In our LHS-PRCC analysis, we conservatively tested a range of 0.01–1 days^-1^ for this parameter. Our results suggest that when using our proposed structure and parameter values there is a threshold for these pneumonic models where outbreaks are either completely successful or fail to spread entirely (Fig 2A). Similarly, transmission rate from fleas to humans (𝛽_b_) correlated positively with total mortality for both bubonic models with rat dynamics (S6 Fig), but this parameter has little empirical support for validating a realistic range.

For bubonic model variants, flea searching efficiency (*α*) consistently correlated negatively with total mortality (S5 and S6 Figs). Previous modeling studies have identified the sensitivity of this model to the interaction between carrying capacity (*K*_*r*_) and flea searching efficiency (*α*) [26,61]. Even so, several modeling studies have treated *α* as a fixed point value independent of density (e.g. [30]) or as a fixed value dependent on rat density without acknowledging its contribution to model outcome (e.g. [16]). Our work suggests that a better empirical understanding of the relationship between rat density and flea search efficiency would be important for future models using this construction to conceptualize encounters between rats and fleas.

### Limitations and future directions

Although we tested a variety of model constructions and conducted a comprehensive global sensitivity analysis, our approach still has several limitations. We investigated multiple hypothesized transmission routes that reflect the modern etiology of *Yersinia pestis*, but these models may still not capture the true epidemiology of the Justinianic Plague (cf. [16,25]). Other studies have highlighted the complexity of the plague transmission cycle. For example, we did not consider the potential of dead hosts (i.e. humans or rats) to serve as reservoirs [30,62], or the myriad of sylvatic hosts contributing to spillover and maintenance of endemic plague [43,63,64]. Similarly, consistent with other modeling studies (e.g. [16,25,26]), we did not differentiate between early phase transmission and blocked transmission, wherein *Y*. *pestis* produces a biofilm blocking the flea foregut, both increasing transmission efficiency and decreasing flea life span [30,62]. Finally, flea life span and *Y*. *pestis* transmission efficiency is temperature dependent [29,34]; we did not explore the consequences of seasonal variation on epidemic outcomes (e.g. [65]). A related issue is that the genetic composition of the *Y*. *pestis* that is associated with the Justinianic Plague is slightly different from that of the current day *Y*. *pestis* [7,17]. We therefore cannot rule out the possibility that the late antique *Y*. *pestis* was more or less lethal to humans.

Other factors that we cannot completely dismiss include possible differences between late antique and modern hygiene, public health practices, and reduced human immunocompetence due to increased risk of comorbidities or poor nutritional status. Discussions in the historical literature have just begun examining the possibility of nutritional stress and coinfection, although data remains anecdotal and incomplete (e.g. [3,66]) We have no data about where in Constantinople plague-related mortality took place, or about specific practices and locations of waste disposal in the city that might be associated with higher rat populations. Although our conservative parameter estimates (Table 1) and LHS approach may capture some of these dimensions, we did not attempt to explicitly model differences in hygiene or comorbidity with different diseases as any such analysis would be conjectural and based on very sparse data at best.

For human birth and natural death rates, we assumed average life expectancy of 25 years (range 20–30 years). In reality, however, non-plague related mortality in this period would have been much higher in infancy and pre-adolescence with the likelihood of surviving to an older age (50–60 years and beyond) improving after these earlier stages of life ([22] citing [67]). Future extensions of this model could relax the assumption of exponentially distributed demographic rates [68]. Our models did not consider any social response to plague, although contemporaries could have mitigated their risk through several simple strategies such as leaving the city or minimizing social contact. The limited data available to us led us to model the most important city within the empire; although the results could, with some reservations, be suggestive of other urban centers in the empire, they would say nothing about the empire’s rural population, which made up the vast majority of its total population.

It is possible that the primary sources that we used to evaluate our models are themselves exaggerated, and that the demographic effects of the Justinianic Plague were far lower than expected. As suggested above, this interpretation is consistent with the general skepticism historians have with regard to premodern primary sources, and with the fact that scholarly estimates of parameter values vary by orders of magnitude even when describing fairly concrete values such as the number of deaths. This line of reasoning has been suggested before, considering the general absence of non-textual evidence for the Justinianic Plague causing extremely high mortality in Constantinople and beyond. While this paper does not prove that these primary sources and maximalist studies are wrong, it casts doubt on such maximalist interpretations.

Although the model results did not corroborate the few details that the primary sources supply, the variability of these model results is potentially important for our broader understanding of the Justinianic Plague and its effects across Mediterranean urban centers. Assuming that the variability in the models’ parameters would reflect variability in the environmental (e.g. temperature, precipitation, humidity), ecological (e.g. rat and flea densities) and social systems (e.g. contact patterns) around the Mediterranean, the spread of the results of all models suggests that plague’s impacts on other urban areas could differ substantially from its impacts on Constantinople. This in turn confirms the fragmentary evidence for the First Pandemic, where certain areas seem to be associated with plague more often than others, and empirical evidence from the Third Pandemic that indicates that plague mortality varied considerably both temporally and spatially (e.g. table in [69]).

Nonetheless, since we evaluated these models in a deterministic framework, we cannot rule out the effects of stochasticity that could lead to probabilistically low, but singularly large outbreaks [27]. Future modeling extensions could extend this framework to allow for stochasticity and more realistic spatial structure. Our models assumed that all the city’s inhabitants mixed homogeneously within the same population; additional realism could be added by incorporating a metapopulation structure to reflect the uneven contact mixing structure of a large urban metropolis (e.g. [25,26]) or by using a spatial network modeling framework to examine how travel and trading routes affect the potential for plague outbreaks [70]).

## Conclusions

In this paper, we have attempted to reconstruct the common scenario of the Justinianic Plague that former literature accepts. According to this scenario, between a quarter and half the population of Constantinople perished during the first outbreak of plague in the city in 542. Despite their general agreement on this conclusion, historians have proposed different models to explain the plague epidemiology that appears in the historical texts that serve as the foundation of modern plague narratives. Instead of selecting one of these epidemiological models, we tested them all with parameter values supported by our modern understanding of plague etiology. While several of the bubonic plague model configurations yielded interquartile estimates arguably consistent with the upper end of maximalist estimates of mortality, plague in these models lasted for considerably less time than suggested by the primary sources. By contrast, both pneumonic models suggest that pneumonic plague by itself was unlikely to cause almost any mortality in Constantinople. When viewed in light of the historical evidence, the model results therefore indicate that the outbreak in Constantinople was very likely not a pneumonic plague outbreak.

Our results suggest that given what we know of modern plague etiology it would have been highly unlikely for a plague outbreak to have the magnitude of impact with the simultaneous outbreak duration that the primary sources describe. Since the outbreak at Constantinople underlies the scholarly understanding of the broader Justinianic Plague and has the most evidence compared to outbreaks elsewhere or later in the first pandemic, our results suggest that the Justinianic Plague behaved differently than the current maximalist consensus postulates, and thus contribute to the broader discussion of the impact of plague during Late Antiquity.

## Supporting information

S1 Fig Time course results of different transmission mode models with rat to human ratio of 1:2. Produced using expected transmission values from Table 1. Initial conditions: number of susceptible humans, *S*_*h*_(*t* = 0) = 500,000, number of susceptible rats, *S*_*r*_(*t* = 0) = 249,999, and number of infected rats, *I*_*r*_(*t* = 0) = 1.
(TIF)

S2 Fig Time course results of different transmission mode models with rat to human ratio of 2:1. Produced using expected transmission values from Table 1. Initial conditions: number of susceptible humans, *S*_*h*_(*t* = 0) = 500,000, number of susceptible rats, *S*_*r*_(*t* = 0) = 999,999, and number of infected rats, *I*_*r*_(*t* = 0) = 1.
(TIF)

S3 Fig Box and whisker plot showing results of uniform LHS sampling. (A) number of human mortalities; and (B) detectable outbreak duration (>100 deaths per day, nonconsecutive) with inset including outliers (up to 5000 days); and (C) detectable outbreak duration (>250 days, nonconsecutive) with inset including outliers (up to 5000 days) zooming in on time axis of 0 to 1000 days. Red lines depict estimated comparison values from historical primary source accounts: (A) 250,000 mortalities; (B) 4 months or 120 days of mortality exceeding 100 deaths per day; and (C) 3 months or 90 days of mortality exceeding 250 deaths per day.
(TIF)

S4 Fig LHS-PRCC results for pneumonic plague SIR and SEIR models. (A) Total mortality for SIR; (B) detectable outbreak duration (days) for SIR; (C) total mortality for SEIR; and (D) detectable outbreak duration (days) for SEIR.
(TIF)

S5 Fig LHS-PRCC results for bubonic plague SIR and SEIR models. (A) Total mortality for SIR; (B) detectable outbreak duration (days) for SIR; (C) total mortality for SEIR; and (D) detectable outbreak duration (days) for SEIR.
(TIF)

S6 Fig LHS-PRCC results for bubonic plague SIR and SEIR models with rat dynamics. (A) Total mortality for SIR; (B) detectable outbreak duration (days) for SIR; (C) total mortality for SEIR; and (D) detectable outbreak duration (days) for SEIR.
(TIF)

S7 Fig LHS-PRCC results for bubonic/pneumonic plague SEIR model. (A) Total mortality for SIR and (B) detectable outbreak duration (days).
(TIF)

S1 Table Summary of model output for each model type with rat to human ratio of 1:2. Outcomes include detectable outbreak duration, maximum mortality rate per day, and total mortality. Initial conditions: number of susceptible humans, *S*_*h*_(*t* = 0) = 500,000, number of susceptible rats, *S*_*r*_(*t* = 0) = 249,999, and number of infected rats, *I*_*r*_(*t* = 0) = 1.
(DOCX)

S2 Table Summary of model output for each model type with rat to human ratio of 2:1. Outcomes include detectable outbreak duration, maximum mortality rate per day, and total mortality. Initial conditions: number of susceptible humans, *S*_*h*_(*t* = 0) = 500,000, number of susceptible rats, *S*_*r*_(*t* = 0) = 999,999, and number of infected rats, *I*_*r*_(*t* = 0) = 1.
(DOCX)

S1 Appendix LHS uniform sampling results. Includes individual scatter plots of parameters vs. model outcomes and PRCC plots.
(DOCX)

S2 Appendix LHS non-uniform sampling results. Includes individual scatter plots of parameters vs. model outcomes and PRCC plots.
(DOCX)
