# Supplementary figures and images for: Modeling the Justinianic Plague: Comparing hypothesized transmission routes
Source: PLoS One. 2020 Apr 30;15(4):e0231256. doi: 10.1371/journal.pone.0231256 (PMC7192389; doi:10.1371/journal.pone.0231256)

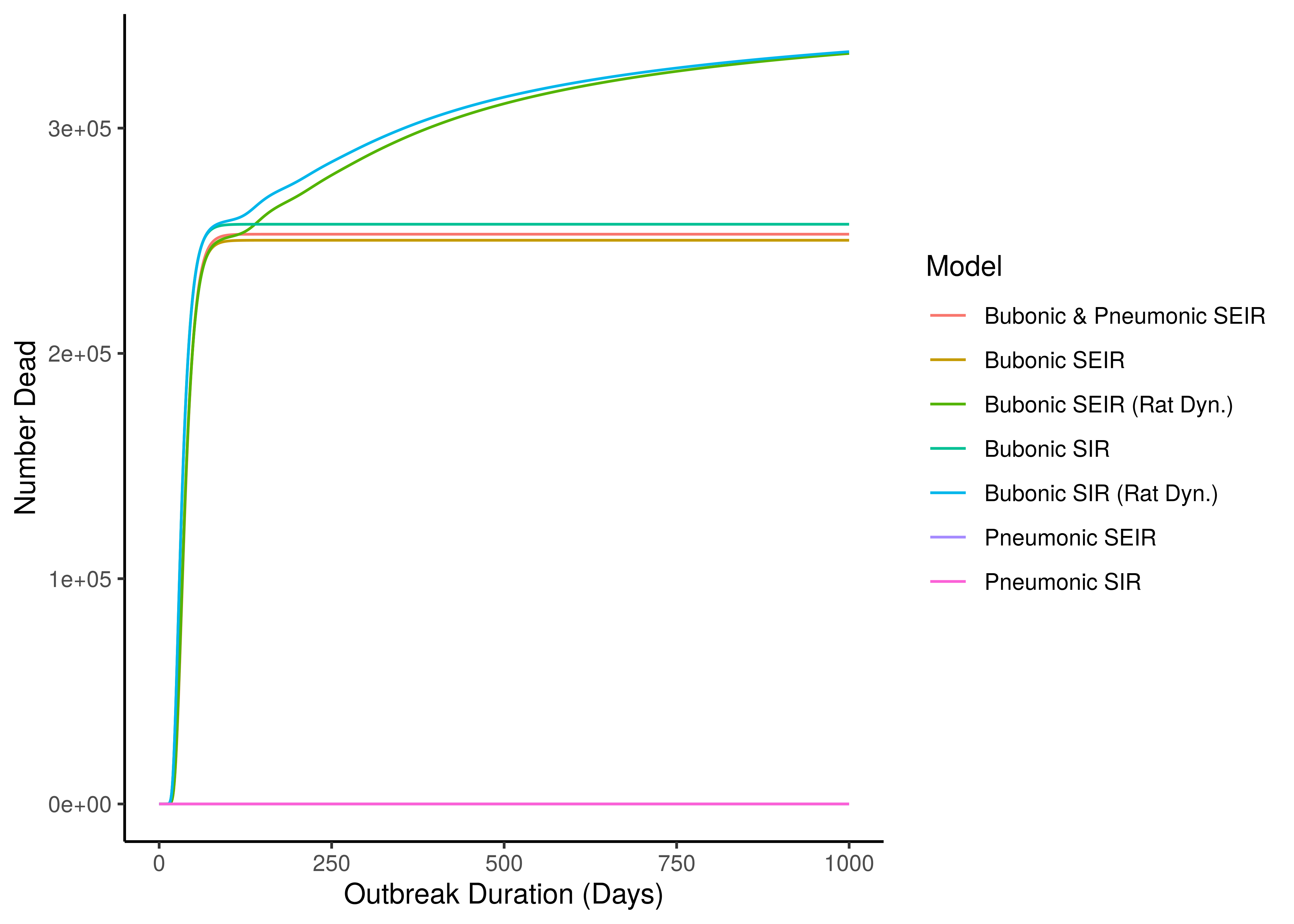

Supplement: S1 Fig — Produced using expected transmission values from Table 1. Initial conditions: number of susceptible humans, Sh(t = 0) = 500,000, number of susceptible rats, Sr(t = 0) = 249,999, and number of infected rats, Ir(t = 0) = 1. (TIF) [file pone.0231256.s001.tif]

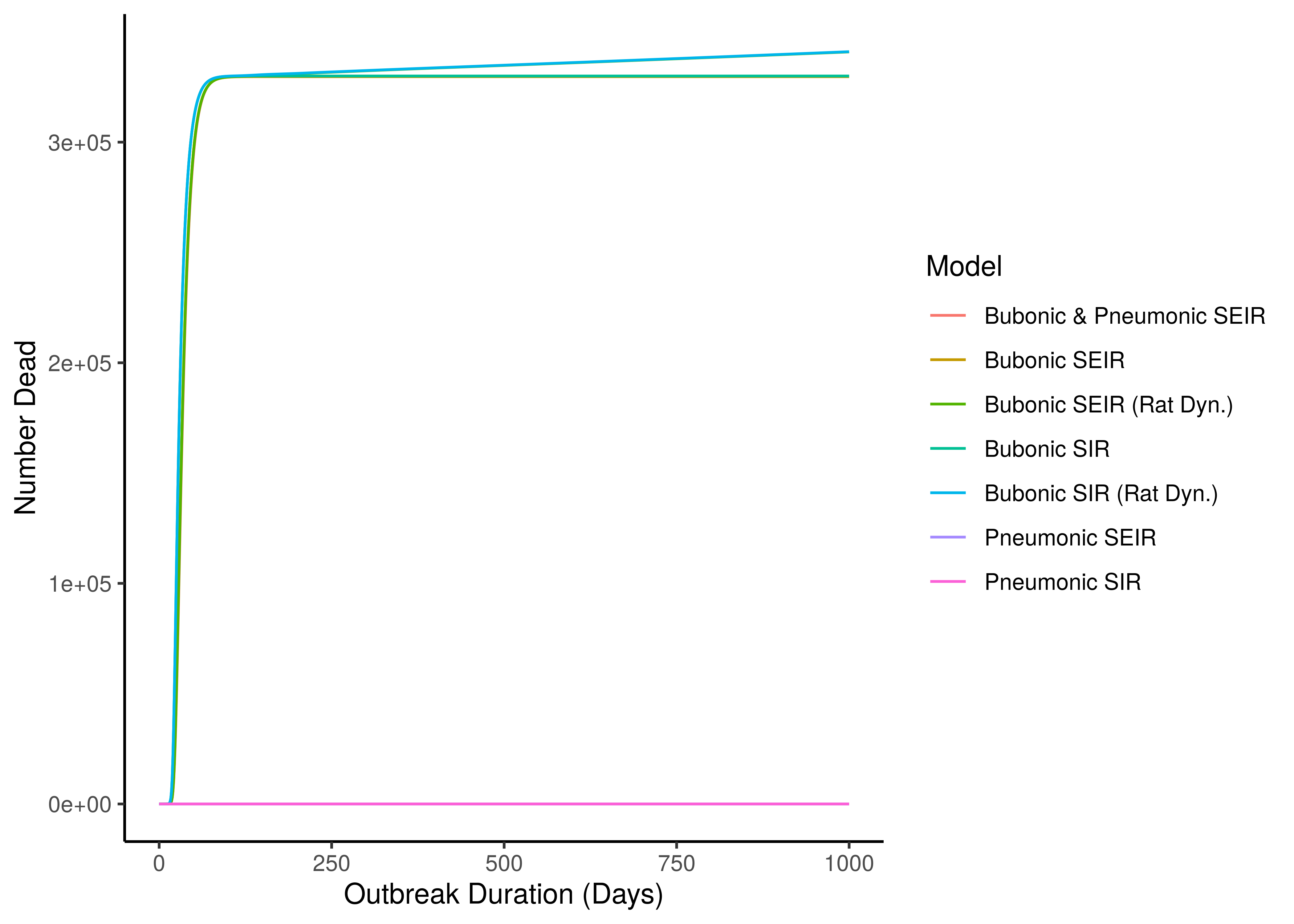

Supplement: S2 Fig — Produced using expected transmission values from Table 1. Initial conditions: number of susceptible humans, Sh(t = 0) = 500,000, number of susceptible rats, Sr(t = 0) = 999,999, and number of infected rats, Ir(t = 0) = 1. (TIF) [file pone.0231256.s002.tif]

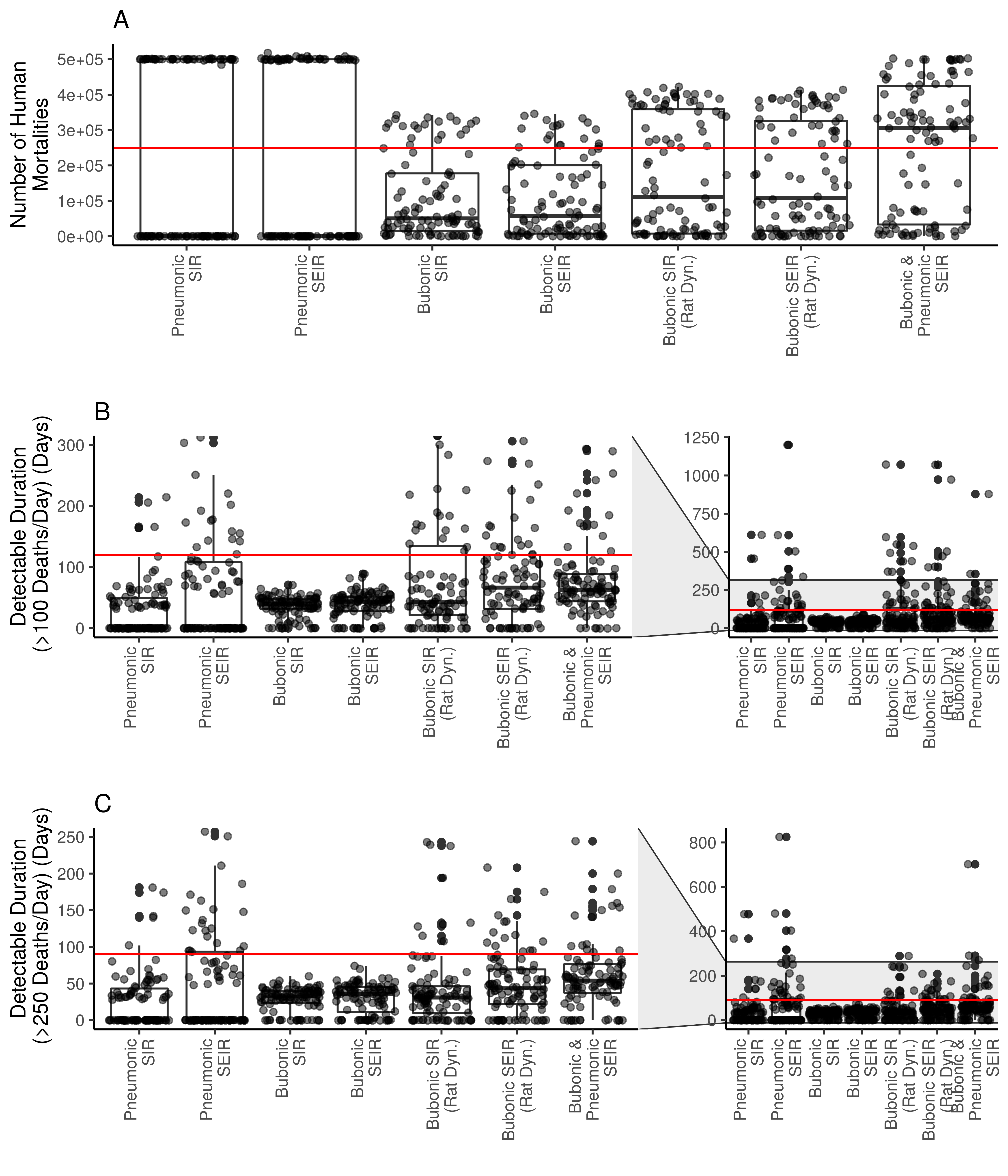

Supplement: S3 Fig — (A) number of human mortalities; and (B) detectable outbreak duration (>100 deaths per day, nonconsecutive) with inset including outliers (up to 5000 days); and (C) detectable outbreak duration (>250 days, nonconsecutive) with inset including outliers (up to 5000 days) zooming in on time axis of 0 to 1000 days. Red lines depict estimated comparison values from historical primary source accounts: (A) 250,000 mortalities; (B) 4 months or 120 days of mortality exceeding 100 deaths per day; and (C) 3 months or 90 days of mortality exceeding 250 deaths per day. (TIF) [file pone.0231256.s003.tif]

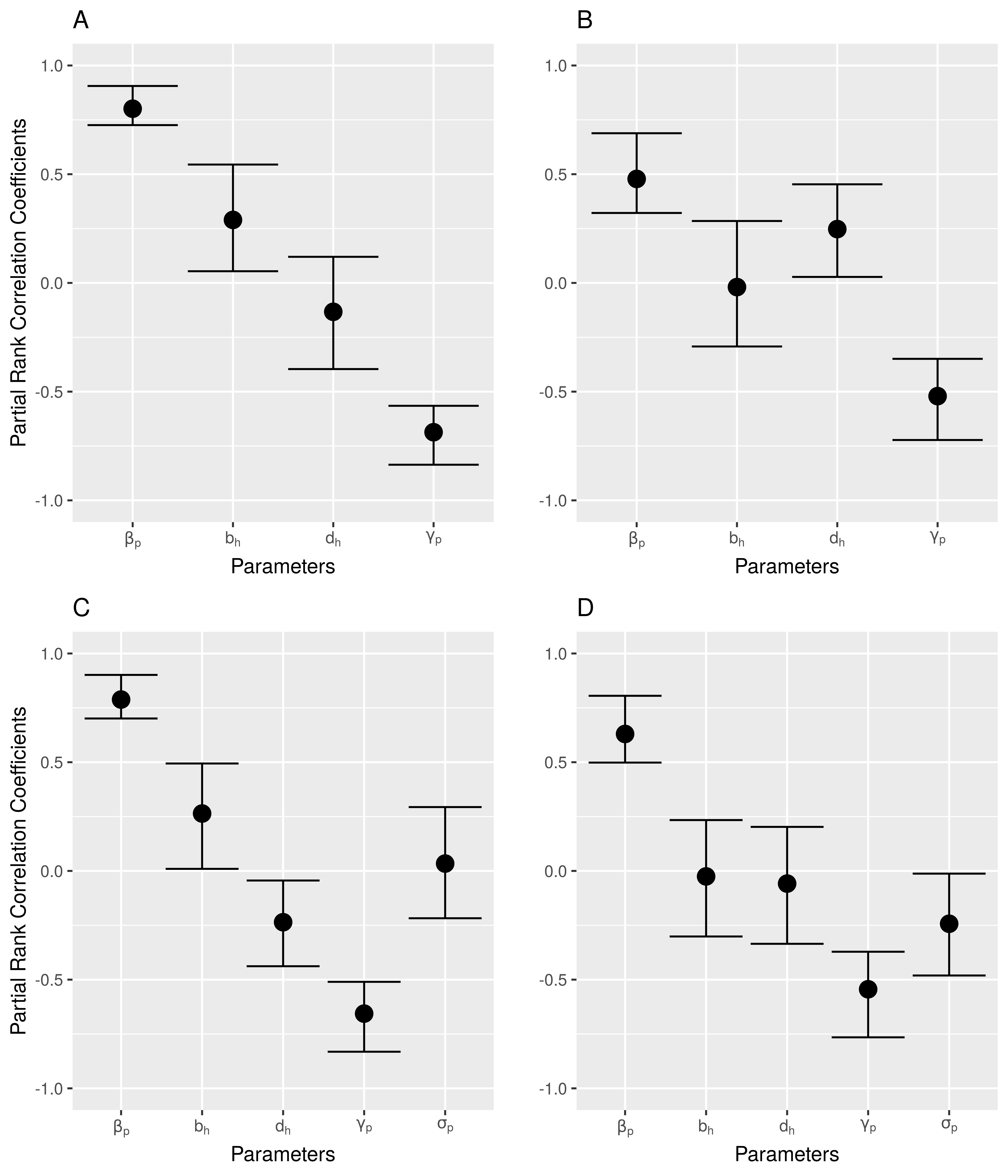

Supplement: S4 Fig — (A) Total mortality for SIR; (B) detectable outbreak duration (days) for SIR; (C) total mortality for SEIR; and (D) detectable outbreak duration (days) for SEIR. (TIF) [file pone.0231256.s004.tif]

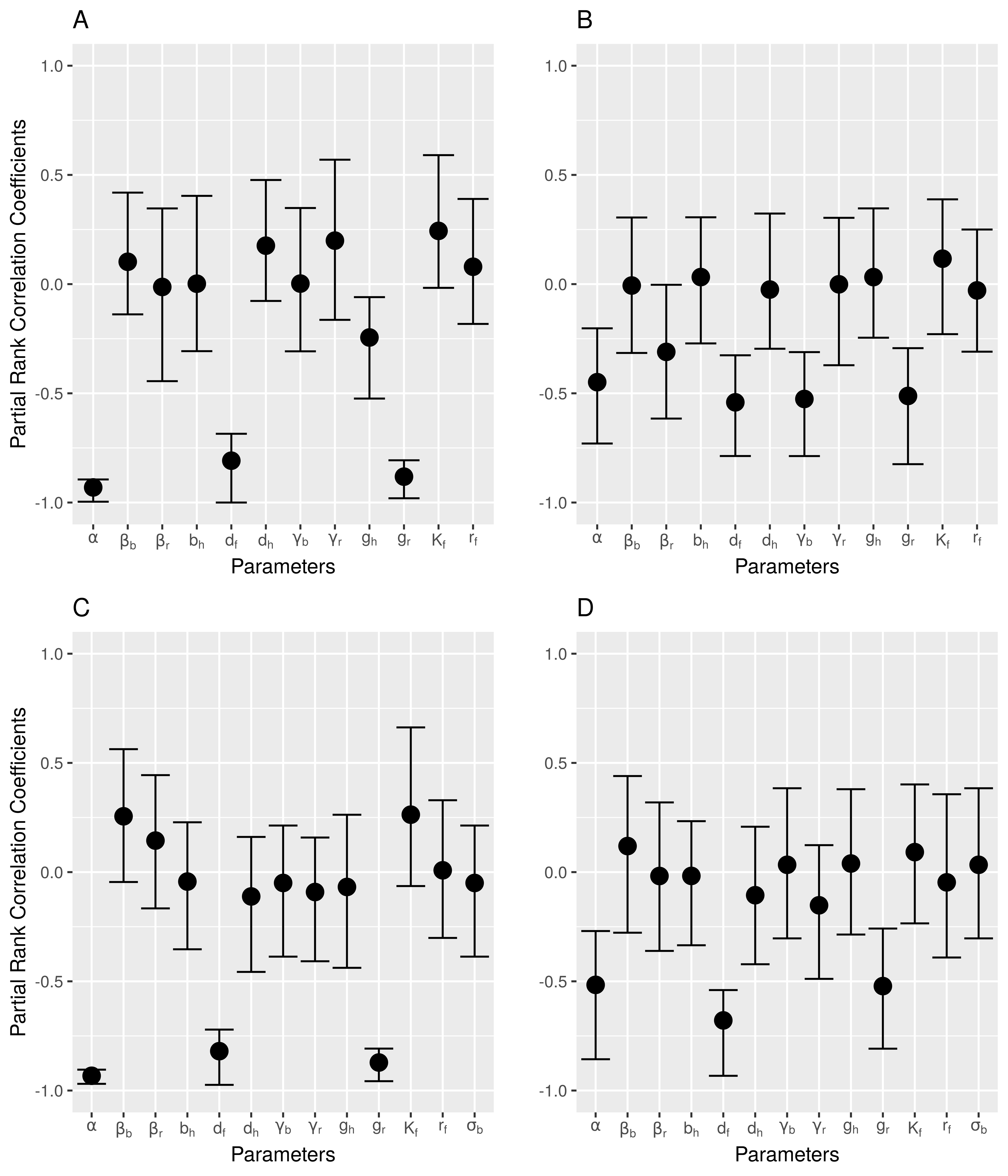

Supplement: S5 Fig — (A) Total mortality for SIR; (B) detectable outbreak duration (days) for SIR; (C) total mortality for SEIR; and (D) detectable outbreak duration (days) for SEIR. (TIF) [file pone.0231256.s005.tif]

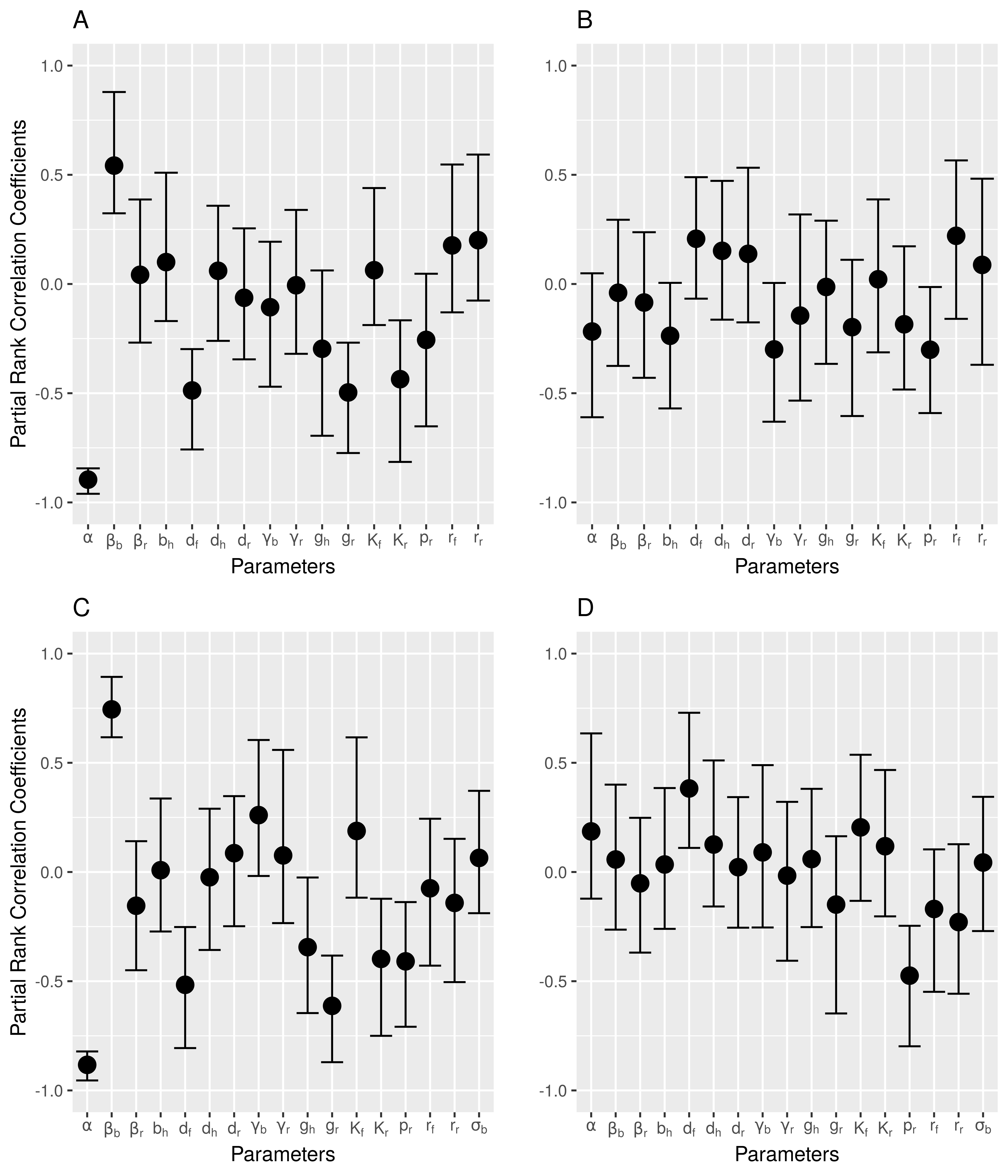

Supplement: S6 Fig — (A) Total mortality for SIR; (B) detectable outbreak duration (days) for SIR; (C) total mortality for SEIR; and (D) detectable outbreak duration (days) for SEIR. (TIF) [file pone.0231256.s006.tif]

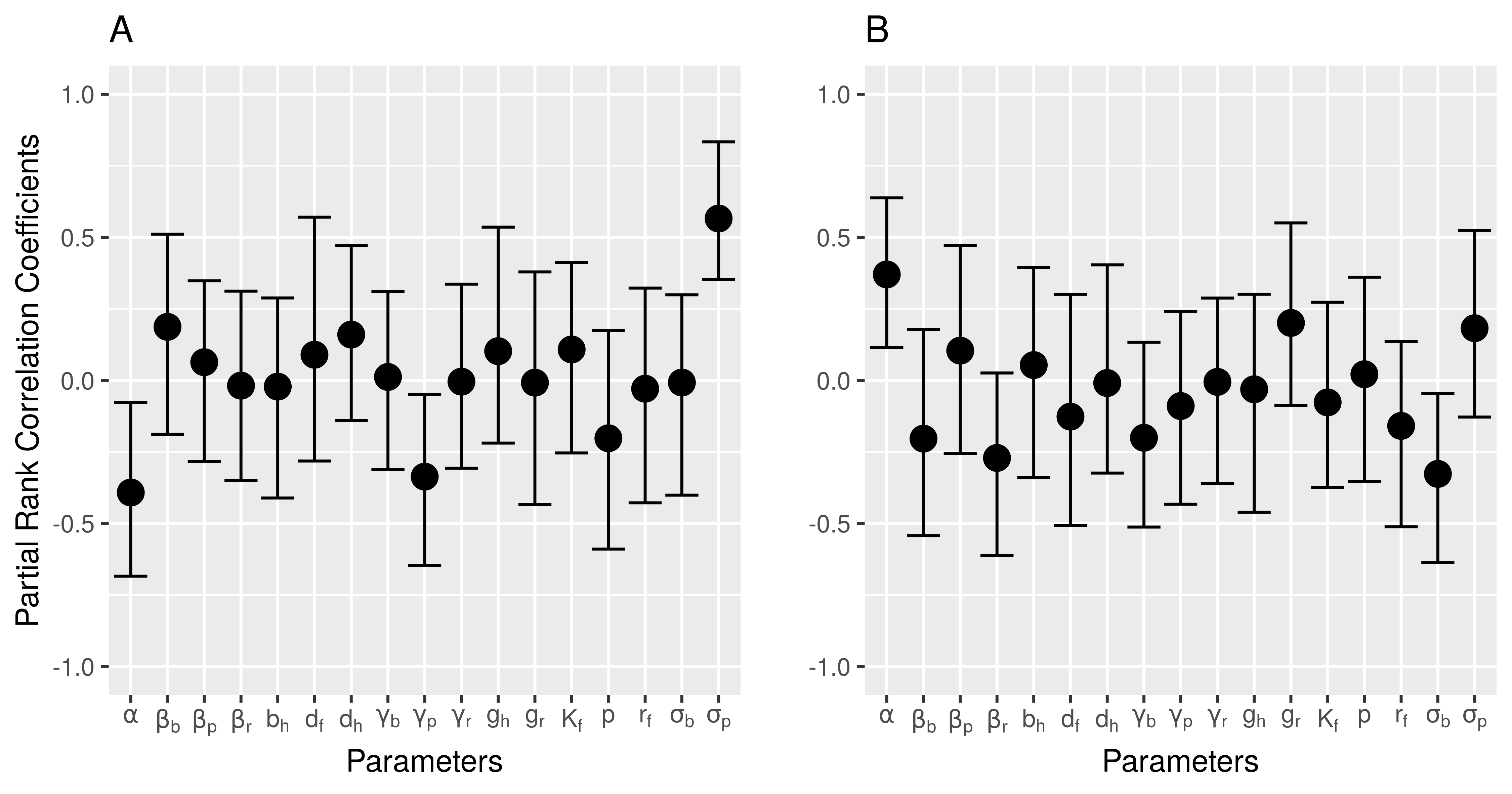

Supplement: S7 Fig — (A) Total mortality for SIR and (B) detectable outbreak duration (days). (TIF) [file pone.0231256.s007.tif]
