# Supplementary material for: Modeling the Justinianic Plague: Comparing hypothesized transmission routes
Source: PLoS One. 2020 Apr 30;15(4):e0231256. doi: 10.1371/journal.pone.0231256 (PMC7192389; doi:10.1371/journal.pone.0231256)
Supplement: S2 Table — Outcomes include detectable outbreak duration, maximum mortality rate per day, and total mortality. Initial conditions: number of susceptible humans, Sh(t = 0) = 500,000, number of susceptible rats, Sr(t = 0) = 999,999, and number of infected rats, Ir(t = 0) = 1. (DOCX) [file pone.0231256.s009.docx]

**S2 Table. Summary of model output for each model type with rat to human ratio of 2:1.**

| Model | Detectable outbreak duration (deaths/day) | | Maximum mortality rate/day | Total mortality (humans) |
| --- | --- | --- | --- | --- |
|  | > 100 | > 250 |  |  |
| Pneumonic SIR | 0 | 0 | 0.34 | 1.25 |
| Pneumonic SEIR | 0 | 0 | 0.33 | 1.25 |
| Bubonic SIR | 67 | 57 | 18862 | 329993 |
| Bubonic SEIR | 70 | 60 | 15370 | 329687 |
| Bubonic SIR (Rat dynamics) | 67 | 57 | 18840 | 341018 |
| Bubonic SEIR (Rat dynamics) | 70 | 60 | 15356 | 340912 |
| Bubonic/Pneumonic SEIR | 71 | 61 | 15097 | 329834 |

Outcomes include detectable outbreak duration, maximum mortality rate per day, and total mortality. Initial conditions: number of susceptible humans, S_h_(t=0)=500,000, number of susceptible rats, S_r_(t=0)=999,999, and number of infected rats, I_r_(t=0)=1.
