# Supplementary material for: Modeling the Justinianic Plague: Comparing hypothesized transmission routes
Source: PLoS One. 2020 Apr 30;15(4):e0231256. doi: 10.1371/journal.pone.0231256 (PMC7192389; doi:10.1371/journal.pone.0231256)
Supplement: S1 Appendix — Includes individual scatter plots of parameters vs. model outcomes and PRCC plots. (DOCX) [file pone.0231256.s010.docx]

S1 Appendix: Uniform Sensitivity Analysis

Lauren White

9/1/2019

# Sensitivity Analysis with Uniform Parameter Distributions

- Begin by creating LHS sampling space with LHSnonuniform.R

#' Adapted from: https://daphnia.ecology.uga.edu/drakelab/wp-content/uploads/2015/07/sensitivity-ebola.pdf
#' load ode functions for each of the models
source('~/JustinianPlague/Plague_model_functions.R')

require(lhs) #add the lhs library
library(sensitivity)
require(ggplot2)
library(tidyverse)
library(ggforce)
require(deSolve)

set.seed(2718) #set random seed
times <- seq(0, 5000, by= 1) #time sequence to integrate over for all models
h <- 100 #choose number of parameter sets/subdivisions to sample to sample
N_r0<-500000 #initial conditions for ODE model- number of rats
niter<-500 #number of times to bootstrap CI for PRCC

#' load uniform and non uniform LHS distributions
source('~/JustinianPlague/LHSnonuniform.R')
uniform<-TRUE #choose uniform (TRUE) or non-uniform (FALSE) distributions

# Latin Hypercube Sampling and Partial Ranked Correlation Coefficients (LHS-PRCC)

- Use GlobalSensitivityAnalysis.R to run ODE models on LHS parameter sets produced in LHSnonuniform.R
- Plot scatter plots of each parameter vs. outbreak size and dectable outbreak duration (days)
- Calculate and plot PRCC values for each parameter for outbreak size and detectable outbreak duration (days)

source('~/JustinianPlague/GlobalSensitivityAnalysis.R')

## Define multiplot function

Use to produce multipaneled ggplot2 figures

#' Multiplot function
# ggplot objects can be passed in ..., or to plotlist (as a list of ggplot objects)
# - cols: Number of columns in layout
# - layout: A matrix specifying the layout. If present, 'cols' is ignored.
#
# If the layout is something like matrix(c(1,2,3,3), nrow=2, byrow=TRUE),
# then plot 1 will go in the upper left, 2 will go in the upper right, and
# 3 will go all the way across the bottom.
#
multiplot <- function(..., plotlist=NULL, file, cols=1, layout=NULL) {
 library(grid)

 # Make a list from the ... arguments and plotlist
 plots <- c(list(...), plotlist)

 numPlots = length(plots)

 # If layout is NULL, then use 'cols' to determine layout
 if (is.null(layout)) {
 # Make the panel
 # ncol: Number of columns of plots
 # nrow: Number of rows needed, calculated from # of cols
 layout <- matrix(seq(1, cols * ceiling(numPlots/cols)),
 ncol = cols, nrow = ceiling(numPlots/cols), byrow=TRUE)
 }

 if (numPlots==1) {
 print(plots[[1]])

 } else {
 # Set up the page
 grid.newpage()
 pushViewport(viewport(layout = grid.layout(nrow(layout), ncol(layout))))

 # Make each plot, in the correct location
 for (i in 1:numPlots) {
 # Get the i,j matrix positions of the regions that contain this subplot
 matchidx <- as.data.frame(which(layout == i, arr.ind = TRUE))

 print(plots[[i]], vp = viewport(layout.pos.row = matchidx$row,
 layout.pos.col = matchidx$col))
 }
 }
}

## Comparative Figure Across Models

# Comparative Figure for All Models ---------------------------------------

comp_size<-data.frame(pSIR=pSIR$MaxInf, pSEIR= pSEIR$MaxInf, bSIR=bSIR$MaxInf, bSEIR= bSEIR$MaxInf, bSIRrK=bSIRrK$MaxInf, bSEIRrK=bSEIRrK$MaxInf, bpSEIR= bpSEIR$MaxInf) #, eSIR=eSIR$MaxInf)
comp_dur<- data.frame(pSIR=pSIR$Thresh100, pSEIR= pSEIR$Thresh100, bSIR=bSIR$Thresh100, bSEIR= bSEIR$Thresh100, bSIRrK=bSIRrK$Thresh100, bSEIRrK=bSEIRrK$Thresh100, bpSEIR= bpSEIR$Thresh100) #, eSIR=eSIR$Thresh100)
comp_dur250<- data.frame(pSIR=pSIR$Thresh250, pSEIR= pSEIR$Thresh250, bSIR=bSIR$Thresh250, bSEIR= bSEIR$Thresh250, bSIRrK=bSIRrK$Thresh250, bSEIRrK=bSEIRrK$Thresh250, bpSEIR= bpSEIR$Thresh250) #, eSIR=eSIR$Thresh250)


long_DFsize <- comp_size %>% gather(Model, NumberDead, c(pSIR, pSEIR, bSIR, bSEIR, bSIRrK, bSEIRrK, bpSEIR))

long_DFdur <- comp_dur %>% gather(Model, Duration, c(pSIR, pSEIR, bSIR, bSEIR, bSIRrK, bSEIRrK, bpSEIR))

long_DFdur250 <- comp_dur250 %>% gather(Model, Duration, c(pSIR, pSEIR, bSIR, bSEIR, bSIRrK, bSEIRrK, bpSEIR))

long_DFsize$Model <- as.character(long_DFsize$Model)
long_DFsize$Model <- factor(long_DFsize$Model, levels=c("pSIR", "pSEIR", "bSIR", "bSEIR", "bSIRrK", "bSEIRrK", "bpSEIR"))

long_DFdur$Model <- as.character(long_DFdur$Model)
long_DFdur$Model <- factor(long_DFdur$Model, levels=c("pSIR", "pSEIR", "bSIR", "bSEIR", "bSIRrK", "bSEIRrK", "bpSEIR"))

long_DFdur250$Model <- as.character(long_DFdur250$Model)
long_DFdur250$Model <- factor(long_DFdur250$Model, levels=c("pSIR", "pSEIR", "bSIR", "bSEIR", "bSIRrK", "bSEIRrK", "bpSEIR"))

A<- ggplot(long_DFsize, aes(Model, NumberDead)) + geom_boxplot()+ geom_jitter(alpha=0.5) +
 ylab("Number of Human\nMortalities")+ xlab("")+
 scale_x_discrete(labels = c(pSIR="Pneumonic\nSIR", pSEIR="Pneumonic\nSEIR", bSIR="Bubonic\nSIR", bSEIR="Bubonic\nSEIR", bSIRrK="Bubonic SIR\n(Rat Dyn.)", bpSEIR="Bubonic &\nPneumonic\nSEIR", bSEIRrK="Bubonic SEIR\n(Rat Dyn.)"))+
 geom_hline(yintercept = 250000, color="red")+
 ggtitle("A")+
 theme_bw() +
 theme(panel.border = element_blank(), panel.grid.major = element_blank(),panel.grid.minor = element_blank(), axis.line = element_line(colour = "black"), axis.text.x = element_text(angle = 90, hjust = 1, vjust=0.5))

B<- ggplot(long_DFdur, aes(Model, Duration)) + geom_boxplot()+ geom_jitter(alpha=0.5) +
 ylab("Detectable Duration\n (>100 Deaths/Day) (Days)")+ xlab("")+
 scale_x_discrete(labels = c(pSIR="Pneumonic\nSIR", pSEIR="Pneumonic\nSEIR", bSIR="Bubonic\nSIR", bSEIR="Bubonic\nSEIR", bSIRrK="Bubonic SIR\n(Rat Dyn.)", bpSEIR="Bubonic &\nPneumonic\nSEIR", bSEIRrK="Bubonic SEIR\n(Rat Dyn.)"))+
 theme(axis.text.x = element_text(angle = 90, hjust = 1))+
 geom_hline(yintercept = 120, color="red")+
 ggtitle("B")+
 theme_bw() +
 theme(panel.border = element_blank(), panel.grid.major = element_blank(),panel.grid.minor = element_blank(), axis.line = element_line(colour = "black"), axis.text.x = element_text(angle = 90, hjust = 1, vjust=0.5))+
 facet_zoom(ylim = c(0, 300))

C<- ggplot(long_DFdur250, aes(Model, Duration)) + geom_boxplot()+ geom_jitter(alpha=0.5) +
 ylab("Detectable Duration\n (>250 Deaths/Day) (Days)")+ xlab("")+
 scale_x_discrete(labels = c(pSIR="Pneumonic\nSIR", pSEIR="Pneumonic\nSEIR", bSIR="Bubonic\nSIR", bSEIR="Bubonic\nSEIR", bSIRrK="Bubonic SIR\n(Rat Dyn.)", bpSEIR="Bubonic &\nPneumonic\nSEIR", bSEIRrK="Bubonic SEIR\n(Rat Dyn.)"))+
 theme(axis.text.x = element_text(angle = 90, hjust = 1))+
 geom_hline(yintercept = 90, color="red")+
 ggtitle("C")+
 theme_bw() +
 theme(panel.border = element_blank(), panel.grid.major = element_blank(),panel.grid.minor = element_blank(), axis.line = element_line(colour = "black"), axis.text.x = element_text(angle = 90, hjust = 1, vjust=0.5))+
 facet_zoom(ylim = c(0, 250))

# multiplot(A, B, C, cols=1)

tiff("S3_Fig.tiff", height =22.23 , width =19.05, units = "cm", compression = "lzw", res = 600)
multiplot(A, B, C, cols=1)
dev.off()

## png
## 2

## Pneumonic Plague SIR

parameters <- c(beta_p = 0.45, gamma_p = 1/2.5, b_h=1/(25*365), d_h=1/(25*365))

#plot scatterplots
par(mfrow=c(1,2))
plot(pSIR$MaxInf~pSIR$beta_p, main= expression(paste("Effect of ", beta[p], " on Size")), xlab=expression(beta[p]), ylab= "Outbreak Size")
plot(pSIR$Thresh100~pSIR$beta_p, main= expression(paste("Effect of ", beta[p], " on Duration")), xlab=expression(beta[p]), ylab= "Observable Duration (days)")


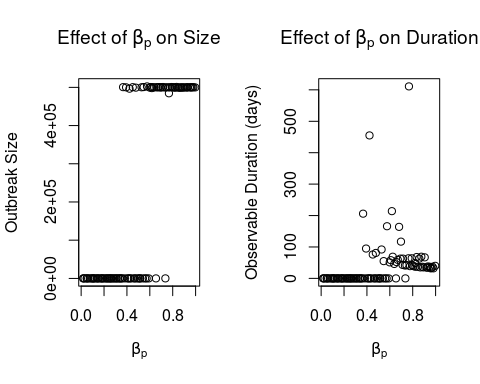


plot(pSIR$MaxInf~pSIR$gamma_p, main= expression(paste("Effect of ", gamma[p], " on Size")), xlab=expression(gamma[p]), ylab= "Outbreak Size")
plot(pSIR$Thresh100~pSIR$gamma_p, main= expression(paste("Effect of ", gamma[p], " on Duration")), xlab=expression(gamma[p]), ylab= "Observable Duration (days)")


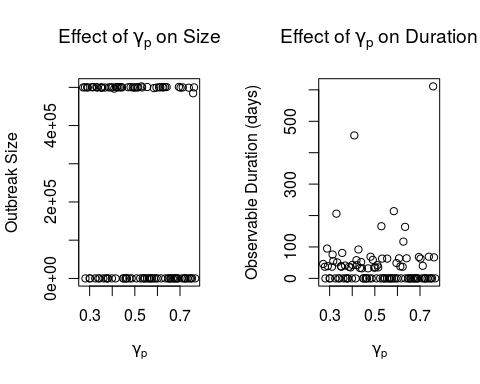


plot(pSIR$MaxInf~pSIR$b_h, main= expression(paste("Effect of ", b[h], " on Size")), xlab=expression(b[h]), ylab= "Outbreak Size")
plot(pSIR$Thresh100~pSIR$b_h, main= expression(paste("Effect of ", b[h], " on Duration")), xlab=expression(b[h]), ylab= "Observable Duration (days)")


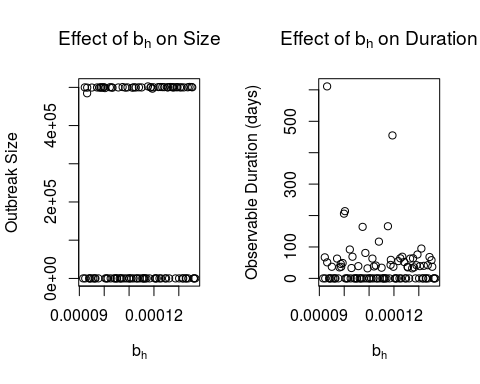


plot(pSIR$MaxInf~pSIR$d_h, main= expression(paste("Effect of ", d[h], " on Size")), xlab=expression(b[h]), ylab= "Outbreak Size")
plot(pSIR$Thresh100~pSIR$d_h, main= expression(paste("Effect of ", d[h], " on Duration")), xlab=expression(d[h]), ylab= "Observable Duration (days)")


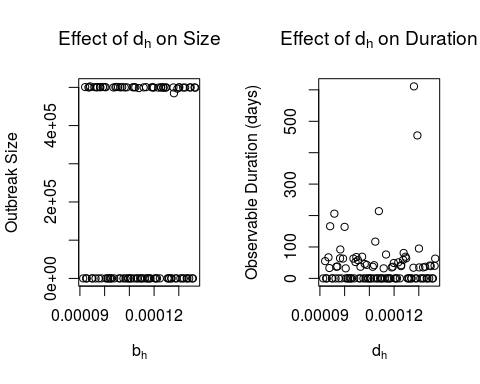


par(mfrow=c(1,2))
boxplot(pSIR$MaxInf, main= "Outbreak Size", ylab= "Number of Dead Humans", ylim=c(0,500000))
boxplot(pSIR$Thresh100, main= "Observable Outbreak Duration", ylab="Time (Days)")


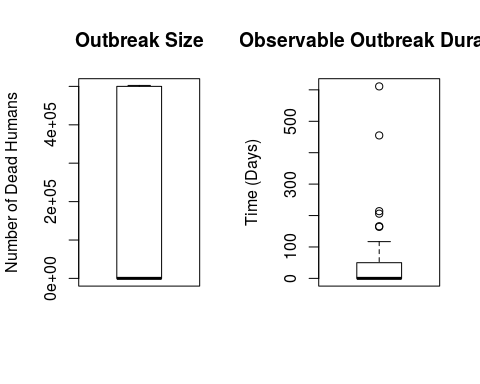


bonferroni.alpha <- 0.05/length(parameters)
prcc_size <- pcc(pSIR[,1:length(parameters)], pSIR$MaxInf, nboot = niter, rank=TRUE, conf=1-bonferroni.alpha)
prcc_duration <- pcc(pSIR[,1:length(parameters)], pSIR$Thresh100, nboot = niter, rank=TRUE, conf=1-bonferroni.alpha)

#plot correlation coefficients and confidence intervals for epidemic size and duration
size<-prcc_size$PRCC
size$param<-rownames(size)
colnames(size)[4:5] <- c("maxCI", "minCI")
size$maxCI[which(size$maxCI>1)]<-1
size$maxCI[which(size$maxCI< -1)]<- -1
size$minCI[which(size$minCI>1)]<-1
size$minCI[which(size$minCI< -1)]<- -1

duration<-prcc_duration$PRCC
duration$param<-rownames(duration)
colnames(duration)[4:5] <- c("maxCI", "minCI")
duration$maxCI[which(duration$maxCI>1)]<-1
duration$maxCI[which(duration$maxCI< -1)]<- -1
duration$minCI[which(duration$minCI>1)]<-1
duration$minCI[which(duration$minCI< -1)]<- -1


A<- ggplot(size, aes(x=param, y = original)) +
 geom_point(size = 4)+
 geom_errorbar(aes(ymax = maxCI, ymin = minCI))+
 ggtitle("A")+
 xlab("Parameters")+
 ylab ("Partial Rank Correlation Coefficients")+
 scale_x_discrete(labels = c("beta_p" = expression(beta[p]),
 "b_h" = expression(b[h]),"d_h" = expression(d[h]), "gamma_p" = expression(gamma[p])))+
 ylim(-1,1)


B<-ggplot(duration, aes(x=param, y = original)) +
 geom_point(size = 4)+
 geom_errorbar(aes(ymax = maxCI, ymin = minCI))+
 ggtitle("B")+
 xlab("Parameters")+
 ylab (" ")+
 scale_x_discrete(labels = c("beta_p" = expression(beta[p]),
 "b_h" = expression(b[h]),"d_h" = expression(d[h]), "gamma_p" = expression(gamma[p])))+
 ylim(-1,1)

multiplot(A, B, cols=2)


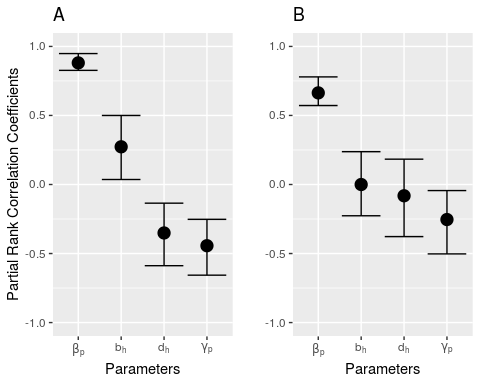


# tiff("FigureS11PneumonicSIR_PRCCuniform.tiff", height =8.7 , width =10, units = "cm", compression = "lzw", res = 1200)
# multiplot(A, B, cols=2)
# dev.off()

## Pneumonic Plague SEIR

parameters <- c(beta_p = 0.45, sigma_p= 1/4.3, gamma_p = 1/2.5, b_h=1/(25*365), d_h=1/(25*365))

par(mfrow=c(1,2))
plot(pSEIR$MaxInf~pSEIR$beta_p, main= expression(paste("Effect of ", beta[p], " on Size")), xlab=expression(beta[p]), ylab="Outbreak Size")
plot(pSEIR$Thresh100~pSEIR$beta_p, main= expression(paste("Effect of ", beta[p], " on Duration")), xlab=expression(beta[p]), ylab="Detectable Outbreak Duration (days)")


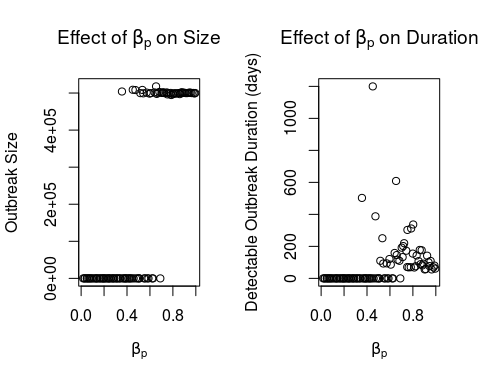


plot(pSEIR$MaxInf~pSEIR$sigma_p, main= expression(paste("Effect of ", sigma[p], " on Size")), xlab=expression(sigma[p]), ylab="Outbreak Size")
plot(pSEIR$Thresh100~pSEIR$sigma_p, main= expression(paste("Effect of ", sigma[p], " on Duration")), xlab=expression(sigma[p]), ylab="Detectable Outbreak Duration (days)")


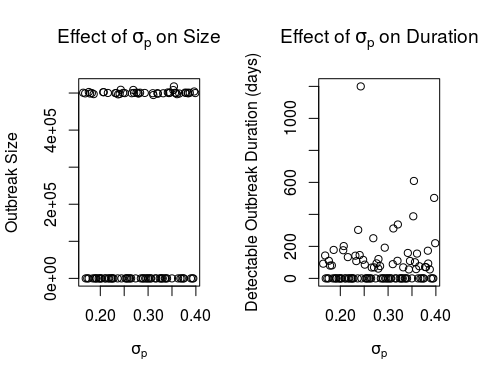


plot(pSEIR$MaxInf~pSEIR$gamma_p, main= expression(paste("Effect of ", gamma[p], " on Size")), xlab=expression(gamma[p]), ylab="Outbreak Size")
plot(pSEIR$Thresh100~pSEIR$gamma_p, main= expression(paste("Effect of ", gamma[p], " on Duration")), xlab=expression(gamma[p]), ylab="Detectable Outbreak Duration (days)")


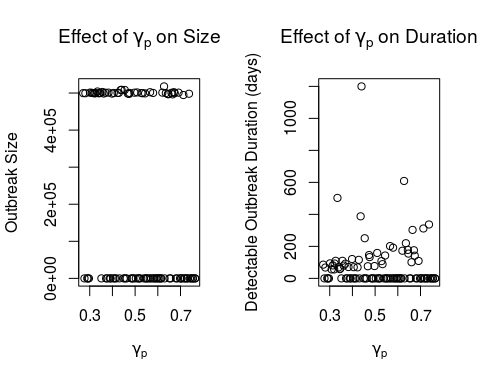


plot(pSEIR$MaxInf~pSEIR$b_h, main= expression(paste("Effect of ", b[h], " on Size")), xlab=expression(b[h]), ylab="Outbreak Size")
plot(pSEIR$Thresh100~pSEIR$b_h, main= expression(paste("Effect of ", b[h], " on Duration")), xlab=expression(b[h]), ylab="Detectable Outbreak Duration (days)")


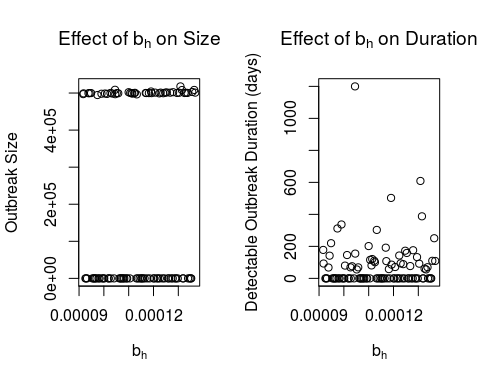


plot(pSEIR$MaxInf~pSEIR$d_h, main= expression(paste("Effect of ", d[h], " on Size")), xlab=expression(d[h]), ylab="Outbreak Size")
plot(pSEIR$Thresh100~pSEIR$d_h, main= expression(paste("Effect of ", d[h], " on Duration")), xlab=expression(d[h]), ylab="Detectable Outbreak Duration (days)")


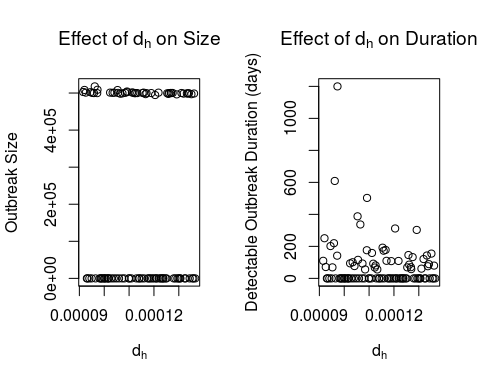


par(mfrow=c(1,2))
boxplot(pSEIR$MaxInf, main= "Outbreak Size", ylab= "Number of Dead Humans", ylim=c(0,500000))
boxplot(pSEIR$Thresh100, main= "Outbreak Duration", ylab="Time (Days)")


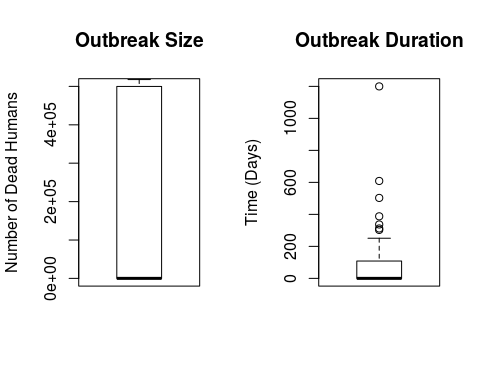


bonferroni.alpha <- 0.05/length(parameters)
prcc_size <- pcc(pSEIR[,1:length(parameters)], pSEIR$MaxInf, nboot = niter, rank=TRUE, conf=1-bonferroni.alpha)
prcc_duration <- pcc(pSEIR[,1:length(parameters)], pSEIR$Thresh100, nboot = niter, rank=TRUE, conf=1-bonferroni.alpha)

#plot correlation coefficients and confidence intervals for epidemic size and duration
size<-prcc_size$PRCC
size$param<-rownames(size)
colnames(size)[4:5] <- c("maxCI", "minCI")
size$maxCI[which(size$maxCI>1)]<-1
size$maxCI[which(size$maxCI< -1)]<- -1
size$minCI[which(size$minCI>1)]<-1
size$minCI[which(size$minCI< -1)]<- -1

duration<-prcc_duration$PRCC
duration$param<-rownames(duration)
colnames(duration)[4:5] <- c("maxCI", "minCI")
duration$maxCI[which(duration$maxCI>1)]<-1
duration$maxCI[which(duration$maxCI< -1)]<- -1
duration$minCI[which(duration$minCI>1)]<-1
duration$minCI[which(duration$minCI< -1)]<- -1

A<- ggplot(size, aes(x=param, y = original)) +
 geom_point(size = 4)+
 geom_errorbar(aes(ymax = maxCI, ymin = minCI))+
 ggtitle("A")+
 xlab("Parameters")+
 ylab ("Partial Rank Correlation Coefficients")+
 scale_x_discrete(labels = c("beta_p" = expression(beta[p]),
 "b_h" = expression(b[h]),"d_h" = expression(d[h]), "gamma_p" = expression(gamma[p]), "sigma_p"=expression(sigma[p])))+
 ylim(-1,1)

B<-ggplot(duration, aes(x=param, y = original)) +
 geom_point(size = 4)+
 geom_errorbar(aes(ymax = maxCI, ymin = minCI))+
 ggtitle("B")+
 xlab("Parameters")+
 ylab (" ")+
 scale_x_discrete(labels = c("beta_p" = expression(beta[p]),
 "b_h" = expression(b[h]),"d_h" = expression(d[h]), "gamma_p" = expression(gamma[p]), "sigma_p"=expression(sigma[p])))+
 ylim(-1,1)

multiplot(A, B, cols=2)


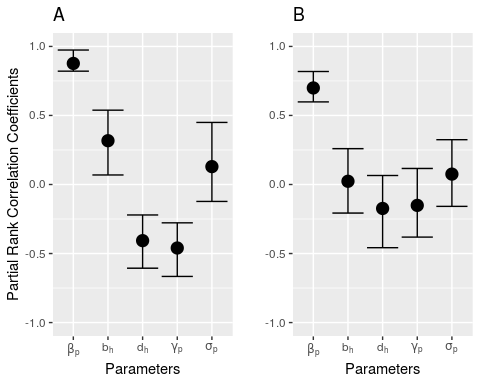


# tiff("FigureS12PneumonicSEIR_PRCCuniform.tiff", height =8.7 , width =10, units = "cm", compression = "lzw", res = 1200)
# multiplot(A, B, cols=2)
# dev.off()

## Bubonic SIR model

parameters <- c(beta_r = 0.09, alpha=3/500000, gamma_r = 1/5.15, g_r=0.1, r_f=0.0084, K_f=6, d_f=1/5, beta_h=0.19, gamma_h=1/10, g_h=0.34, b_h=1/(25*365), d_h=1/(25*365)) #you can play with transmission and recovery rates here

par(mfrow=c(1,2))
plot(bSIR$MaxInf~bSIR$beta_r, main= expression(paste("Effect of ", beta[r], " on Size")), xlab=expression(beta[r]), ylab="Outbreak Size")
plot(bSIR$Thresh100~bSIR$beta_r, main= expression(paste("Effect of ", beta[r], " on Duration")), xlab=expression(beta[r]), ylab="Detectable Duration (days)")


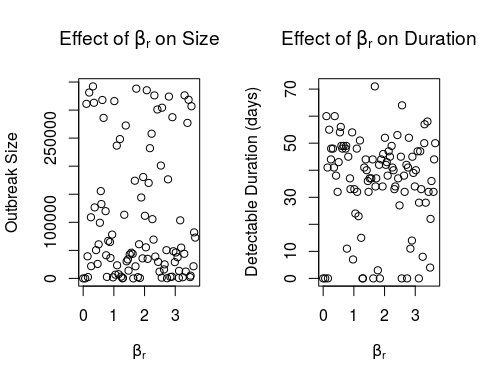


plot(bSIR$MaxInf~bSIR$alpha, main= expression(paste("Effect of ", alpha, " on Size")), xlab=expression(alpha), ylab="Outbreak Size")
plot(bSIR$Thresh100~bSIR$alpha, main= expression(paste("Effect of ", alpha, " on Duration")), xlab=expression(alhpa), ylab="Detectable Duration (days)")


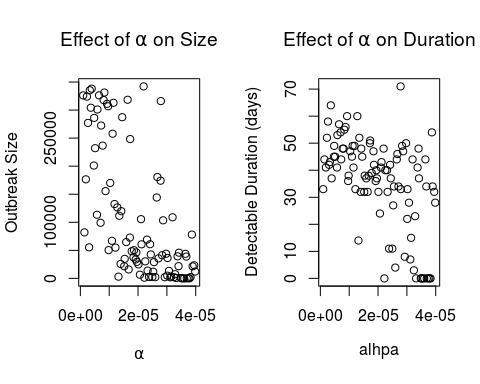


plot(bSIR$MaxInf~bSIR$gamma_r, main= expression(paste("Effect of ", gamma[r], " on Size")), xlab=expression(gamma[r]), ylab="Outbreak Size")
plot(bSIR$Thresh100~bSIR$gamma_r, main= expression(paste("Effect of ", gamma[r], " on Duration")), xlab=expression(gamma[r]), ylab="Detectable Duration (days)")


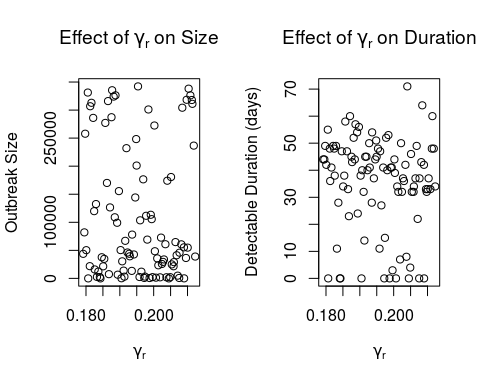


plot(bSIR$MaxInf~bSIR$g_r, main= expression(paste("Effect of ", g[r], " on Size")), xlab=expression(g[r]), ylab="Outbreak Size")
plot(bSIR$Thresh100~bSIR$g_r, main= expression(paste("Effect of ", g[r], " on Duration")), xlab=expression(g[r]), ylab="Detectable Duration (days)")


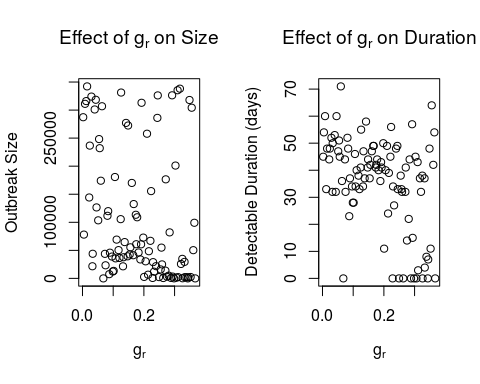


plot(bSIR$MaxInf~bSIR$r_f, main= expression(paste("Effect of ", r[f], " on Size")), xlab=expression(r[f]), ylab="Outbreak Size")
plot(bSIR$Thresh100~bSIR$r_f, main= expression(paste("Effect of ", r[f], " on Duration")), xlab=expression(r[f]), ylab="Detectable Duration (days)")


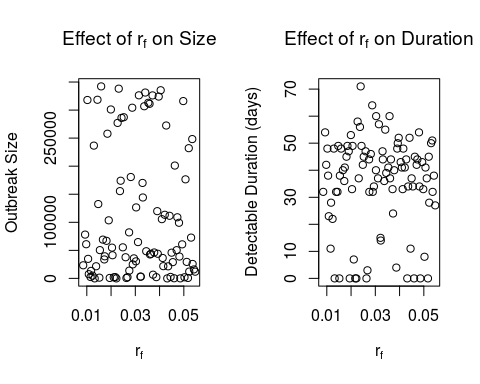


plot(bSIR$MaxInf~bSIR$K_f, main= expression(paste("Effect of ", K[f], " on Size")), xlab=expression(K[f]), ylab="Outbreak Size")
plot(bSIR$Thresh100~bSIR$K_f, main= expression(paste("Effect of ", K[f], " on Duration")), xlab=expression(K[f]), ylab="Detectable Duration (days)")


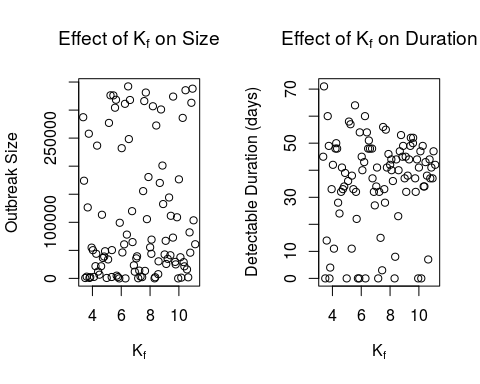


plot(bSIR$MaxInf~bSIR$d_f, main= expression(paste("Effect of ", d[f], " on Size")), xlab=expression(d[f]), ylab="Outbreak Size")
plot(bSIR$Thresh100~bSIR$d_f, main= expression(paste("Effect of ", d[f], " on Duration")), xlab=expression(d[f]), ylab="Detectable Duration (days)")


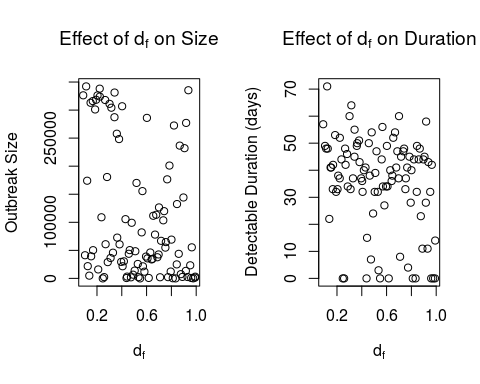


plot(bSIR$MaxInf~bSIR$beta_h, main= expression(paste("Effect of ", beta[b], " on Size")), xlab=expression(beta[b]), ylab="Outbreak Size")
plot(bSIR$Thresh100~bSIR$beta_h, main= expression(paste("Effect of ", beta[b], " on Duration")), xlab=expression(beta[b]), ylab="Detectable Duration (days)")


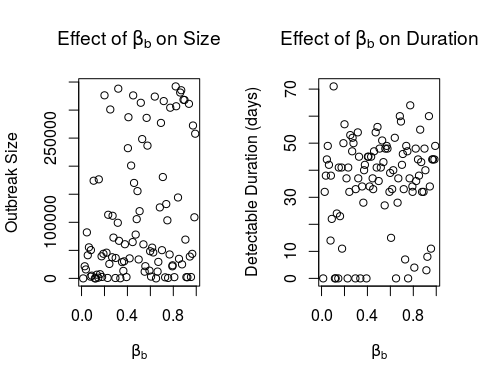


plot(bSIR$MaxInf~bSIR$gamma_h, main= expression(paste("Effect of ", gamma[b], " on Size")), xlab=expression(gamma[b]), ylab="Outbreak Size")
plot(bSIR$Thresh100~bSIR$gamma_h, main= expression(paste("Effect of ", gamma[b], " on Duration")), xlab=expression(gamma[b]), ylab="Detectable Duration (days)")


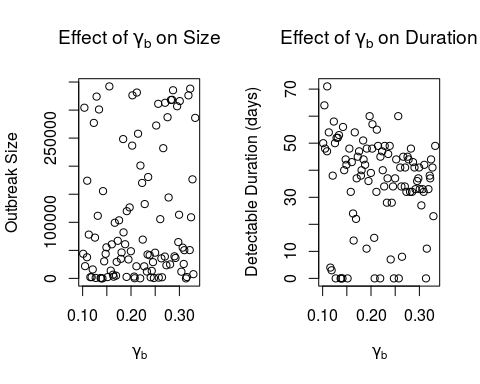


plot(bSIR$MaxInf~bSIR$g_h, main= expression(paste("Effect of ", g[h], " on Size")), xlab=expression(g[h]), ylab="Outbreak Size")
plot(bSIR$Thresh100~bSIR$g_h, main= expression(paste("Effect of ", g[h], " on Duration")), xlab=expression(g[h]), ylab="Detectable Duration (days)")


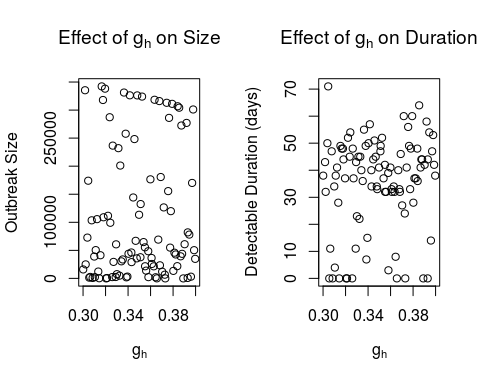


plot(bSIR$MaxInf~bSIR$b_h, main= expression(paste("Effect of ", b[h], " on Size")), xlab=expression(b[h]), ylab="Outbreak Size")
plot(bSIR$Thresh100~bSIR$b_h, main= expression(paste("Effect of ", b[h], " on Duration")), xlab=expression(b[h]), ylab="Detectable Duration (days)")


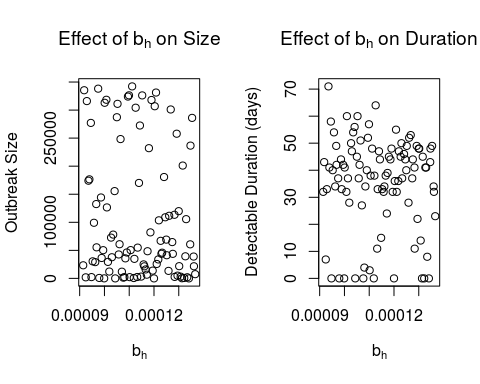


plot(bSIR$MaxInf~bSIR$d_h, main= expression(paste("Effect of ", d[h], " on Size")), xlab=expression(d[h]), ylab="Outbreak Size")
plot(bSIR$Thresh100~bSIR$d_h, main= expression(paste("Effect of ", d[h], " on Duration")), xlab=expression(d[h]), ylab="Detectable Duration (days)")


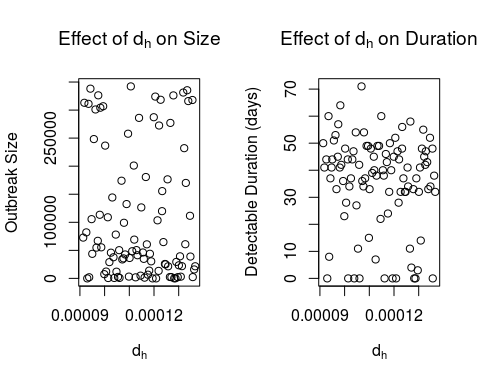


par(mfrow=c(1,2))
boxplot(bSIR$MaxInf, main= "Outbreak Size", ylab= "Number of Dead Humans", ylim=c(0,500000))
boxplot(bSIR$Thresh100, main= "Outbreak Duration", ylab="Time (Days)")


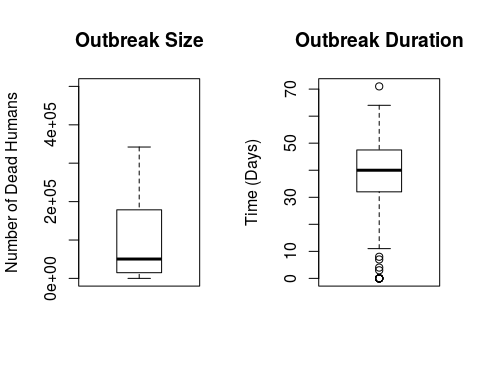


bonferroni.alpha <- 0.05/length(parameters)
prcc_size <- pcc(bSIR[,1:length(parameters)], bSIR$MaxInf, nboot = niter, rank=TRUE, conf=1-bonferroni.alpha)
prcc_duration <- pcc(bSIR[,1:length(parameters)], bSIR$Thresh100, nboot = niter, rank=TRUE, conf=1-bonferroni.alpha)

#plot correlation coefficients and confidence intervals for epidemic size and duration
size<-prcc_size$PRCC
size$param<-rownames(size)
colnames(size)[4:5] <- c("maxCI", "minCI")
size$maxCI[which(size$maxCI>1)]<-1
size$maxCI[which(size$maxCI< -1)]<- -1
size$minCI[which(size$minCI>1)]<-1
size$minCI[which(size$minCI< -1)]<- -1

duration<-prcc_duration$PRCC
duration$param<-rownames(duration)
colnames(duration)[4:5] <- c("maxCI", "minCI")
duration$maxCI[which(duration$maxCI>1)]<-1
duration$maxCI[which(duration$maxCI< -1)]<- -1
duration$minCI[which(duration$minCI>1)]<-1
duration$minCI[which(duration$minCI< -1)]<- -1


A<- ggplot(size, aes(x=param, y = original)) +
 geom_point(size = 4)+
 geom_errorbar(aes(ymax = maxCI, ymin = minCI))+
 ggtitle("A")+
 xlab("Parameters")+
 ylab ("Partial Rank Correlation Coefficients")+
 scale_x_discrete(labels = c("alpha"=expression(alpha),"beta_h" = expression(beta[b]), "beta_r"=expression(beta[r]),
 "b_h" = expression(b[h]),"d_h" = expression(d[h]), "d_f"=expression(d[f]), "gamma_h" = expression(gamma[b]), "gamma_r" = expression(gamma[r]), "g_h"=expression(g[h]), "g_r"=expression(g[r]), "K_f"=expression(K[f]), "r_f"=expression(r[f])))+
 ylim(-1,1)

B<-ggplot(duration, aes(x=param, y = original)) +
 geom_point(size = 4)+
 geom_errorbar(aes(ymax = maxCI, ymin = minCI))+
 ggtitle("B")+
 xlab("Parameters")+
 ylab (" ")+
 scale_x_discrete(labels = c("alpha"=expression(alpha),"beta_h" = expression(beta[b]), "beta_r"=expression(beta[r]),
 "b_h" = expression(b[h]),"d_h" = expression(d[h]), "d_f"=expression(d[f]), "gamma_h" = expression(gamma[b]), "gamma_r" = expression(gamma[r]), "g_h"=expression(g[h]), "g_r"=expression(g[r]), "K_f"=expression(K[f]), "r_f"=expression(r[f])))+
 ylim(-1,1)

multiplot(A, B, cols=2)


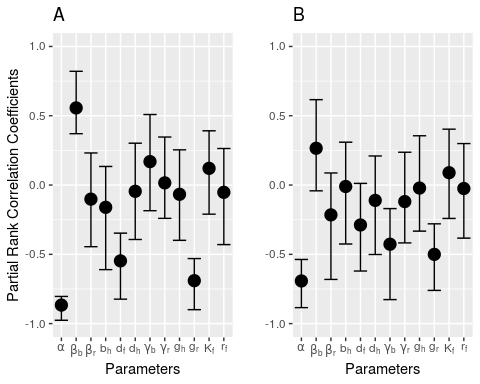


# tiff("FigurS13BubonicSIR_PRCCuniform.tiff", height =8.7 , width =10, units = "cm", compression = "lzw", res = 1200)
# multiplot(A, B, cols=2)
# dev.off()

## Bubonic SEIR

parameters <- c(beta_r = 0.09, alpha=3/500000, gamma_r = 1/5.15, g_r=0.1, r_f=0.0084, K_f=6, d_f=1/5, beta_h=0.19, sigma_h= 1/4, gamma_h=1/10, g_h=0.34, b_h=1/(25*365), d_h=1/(25*365)) #you can play with transmission and recovery rates here

par(mfrow=c(1,2))
plot(bSEIR$MaxInf~bSEIR$beta_r, main= expression(paste("Effect of ", beta[r], " on Size")), xlab=expression(beta[r]), ylab="Outbreak Size")
plot(bSEIR$Thresh100~bSEIR$beta_r, main= expression(paste("Effect of ", beta[r], " on Duration")), xlab=expression(beta[r]), ylab="Detectable Duration (days)")


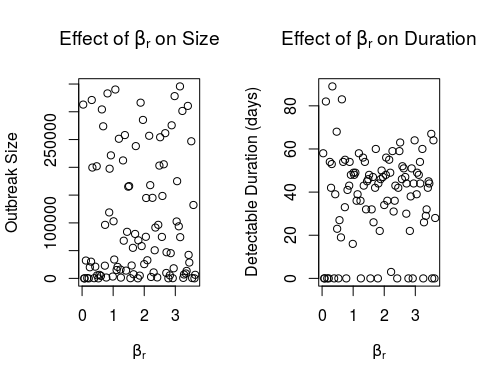


plot(bSEIR$MaxInf~bSEIR$alpha, main= expression(paste("Effect of ", alpha, " on Size")), xlab=expression(alpha), ylab="Outbreak Size")
plot(bSEIR$Thresh100~bSEIR$alpha, main= expression(paste("Effect of ", alpha, " on Duration")), xlab=expression(alpha), ylab="Detectable Duration (days)")


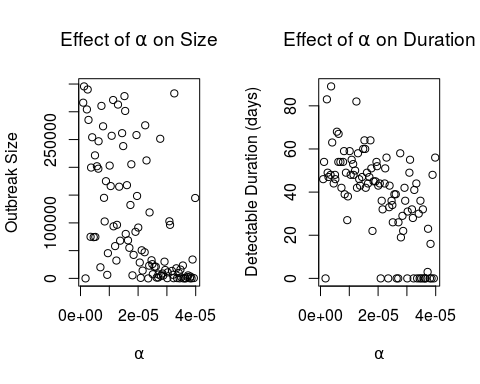


plot(bSEIR$MaxInf~bSEIR$gamma_r, main= expression(paste("Effect of ", gamma[r], " on Size")), xlab=expression(gamma[r]), ylab="Outbreak Size")
plot(bSEIR$Thresh100~bSEIR$gamma_r, main= expression(paste("Effect of ", gamma[r], " on Duration")), xlab=expression(gamma[r]), ylab="Detectable Duration (days)")


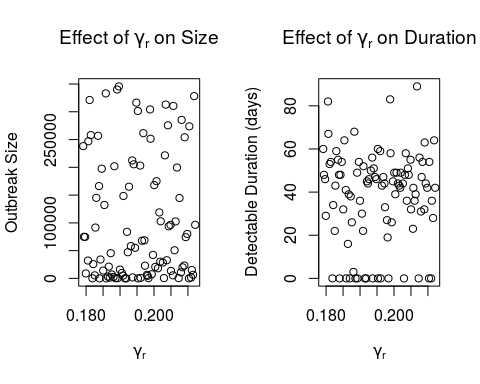


plot(bSEIR$MaxInf~bSEIR$g_r, main= expression(paste("Effect of ", g[r], " on Size")), xlab=expression(g[r]), ylab="Outbreak Size")
plot(bSEIR$Thresh100~bSEIR$g_r, main= expression(paste("Effect of ", g[r], " on Duration")), xlab=expression(g[r]), ylab="Detectable Duration (days)")


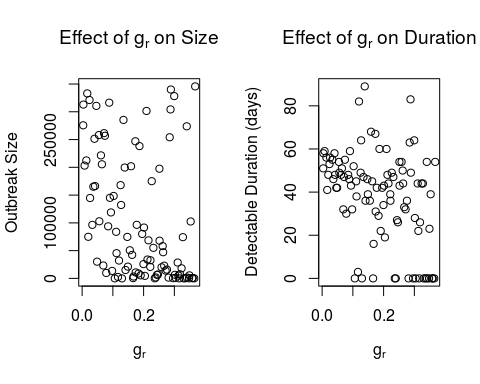


plot(bSEIR$MaxInf~bSEIR$r_f, main= expression(paste("Effect of ", r[f], " on Size")), xlab=expression(r[f]), ylab="Outbreak Size")
plot(bSEIR$Thresh100~bSEIR$r_f, main= expression(paste("Effect of ", r[f], " on Duration")), xlab=expression(r[f]), ylab="Detectable Duration (days)")


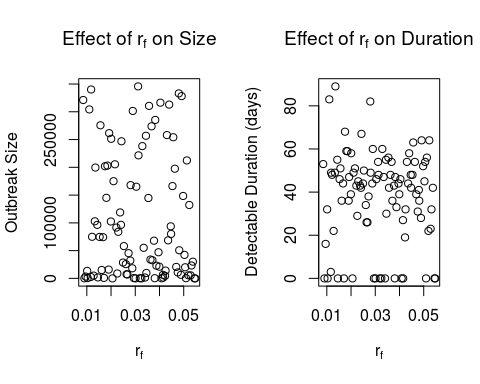


plot(bSEIR$MaxInf~bSEIR$K_f, main= expression(paste("Effect of ", K[f], " on Size")), xlab=expression(K[f]), ylab="Outbreak Size")
plot(bSEIR$Thresh100~bSEIR$K_f, main= expression(paste("Effect of ", K[f], " on Duration")), xlab=expression(K[f]), ylab="Detectable Duration (days)")


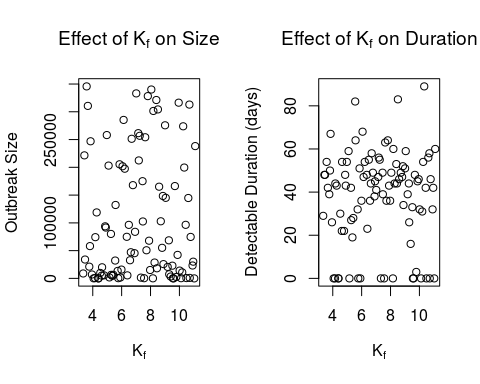


plot(bSEIR$MaxInf~bSEIR$d_f, main= expression(paste("Effect of ", d[f], " on Size")), xlab=expression(d[f]), ylab="Outbreak Size")
plot(bSEIR$Thresh100~bSEIR$d_f, main= expression(paste("Effect of ", d[f], " on Duration")), xlab=expression(d[f]), ylab="Detectable Duration (days)")


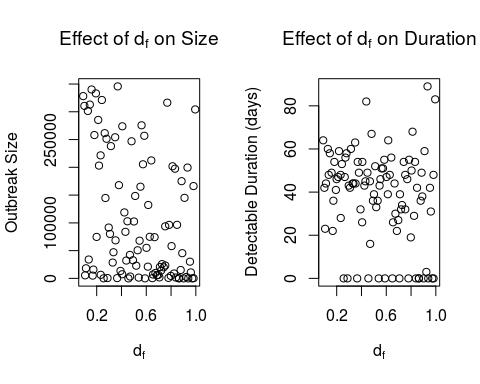


plot(bSEIR$MaxInf~bSEIR$beta_h, main= expression(paste("Effect of ", beta[b], " on Size")), xlab=expression(beta[b]), ylab="Outbreak Size")
plot(bSEIR$Thresh100~bSEIR$beta_h, main= expression(paste("Effect of ", beta[b], " on Duration")), xlab=expression(beta[b]), ylab="Detectable Duration (days)")


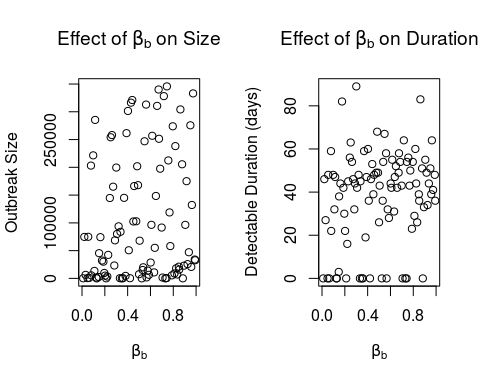


plot(bSEIR$MaxInf~bSEIR$gamma_h, main= expression(paste("Effect of ", gamma[b], " on Size")), xlab=expression(gamma[b]), ylab="Outbreak Size")
plot(bSEIR$Thresh100~bSEIR$gamma_h, main= expression(paste("Effect of ", gamma[b], " on Duration")), xlab=expression(gamma[b]), ylab="Detectable Duration (days)")


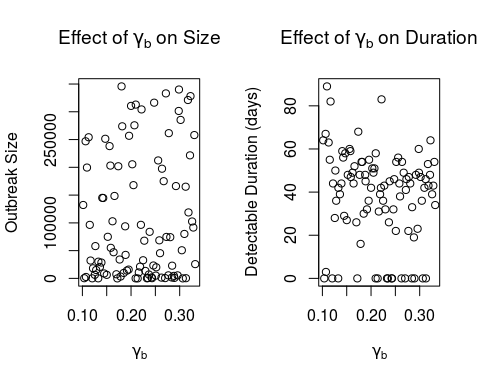


plot(bSEIR$MaxInf~bSEIR$g_h, main= expression(paste("Effect of ", g[h], " on Size")), xlab=expression(g[h]), ylab="Outbreak Size")
plot(bSEIR$Thresh100~bSEIR$g_h, main= expression(paste("Effect of ", g[h], " on Duration")), xlab=expression(g[h]), ylab="Detectable Duration (days)")


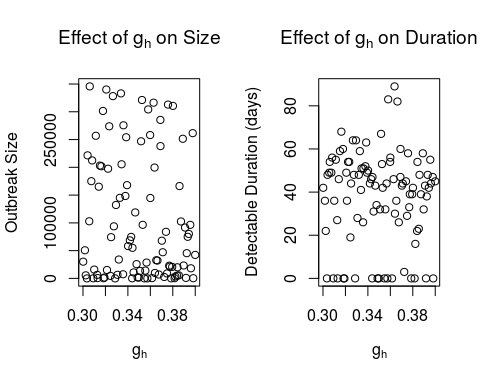


plot(bSEIR$MaxInf~bSEIR$b_h, main= expression(paste("Effect of ", b[h], " on Size")), xlab=expression(b[h]), ylab="Outbreak Size")
plot(bSEIR$Thresh100~bSEIR$b_h, main= expression(paste("Effect of ", b[h], " on Duration")), xlab=expression(b[h]), ylab="Detectable Duration (days)")


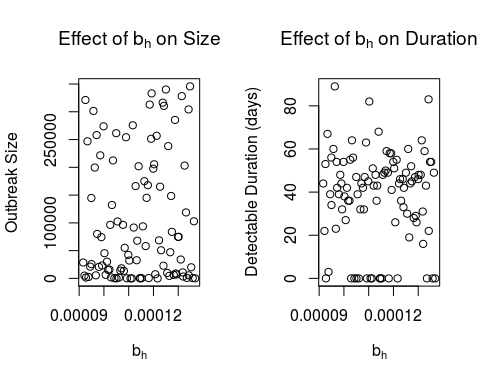


plot(bSEIR$MaxInf~bSEIR$d_h, main= expression(paste("Effect of ", d[h], " on Size")), xlab=expression(d[h]), ylab="Outbreak Size")
plot(bSEIR$Thresh100~bSEIR$d_h, main= expression(paste("Effect of ", d[h], " on Duration")), xlab=expression(d[h]), ylab="Detectable Duration (days)")


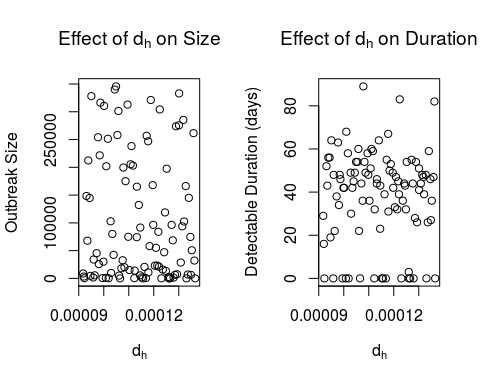


plot(bSEIR$MaxInf~bSEIR$sigma_h, main= expression(paste("Effect of ", sigma[b], " on Size")), xlab=expression(sigma_h[h]), ylab="Outbreak Size")
plot(bSEIR$Thresh100~bSEIR$sigma_h, main= expression(paste("Effect of ", sigma[b], " on Duration")), xlab=expression(sigma[b]), ylab="Detectable Duration (days)")


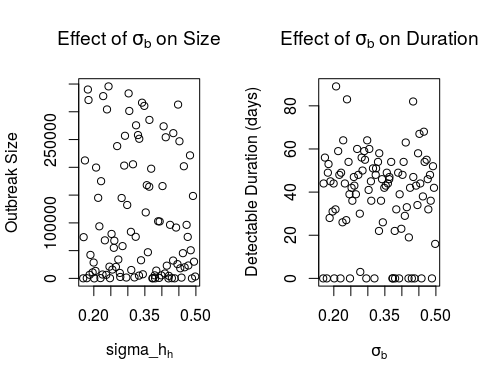


par(mfrow=c(1,2))
boxplot(bSEIR$MaxInf, main= "Outbreak Size", ylab= "Number of Dead Humans", ylim=c(0,500000))
boxplot(bSEIR$Thresh100, main= "Outbreak Duration", ylab="Time (Days)")


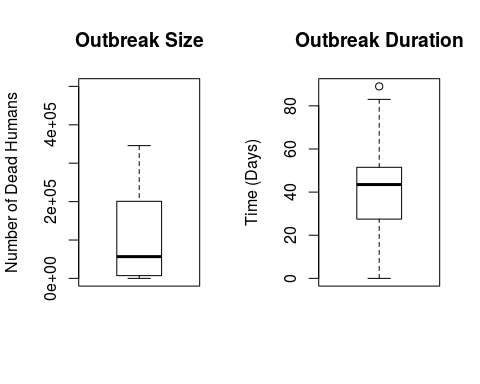


bonferroni.alpha <- 0.05/length(parameters)
prcc_size <- pcc(bSEIR[,1:length(parameters)], bSEIR$MaxInf, nboot = niter, rank=TRUE, conf=1-bonferroni.alpha)
prcc_duration <- pcc(bSEIR[,1:length(parameters)], bSEIR$Thresh100, nboot = niter, rank=TRUE, conf=1-bonferroni.alpha)

#plot correlation coefficients and confidence intervals for epidemic size and duration
size<-prcc_size$PRCC
size$param<-rownames(size)
colnames(size)[4:5] <- c("maxCI", "minCI")
size$maxCI[which(size$maxCI>1)]<-1
size$maxCI[which(size$maxCI< -1)]<- -1
size$minCI[which(size$minCI>1)]<-1
size$minCI[which(size$minCI< -1)]<- -1

duration<-prcc_duration$PRCC
duration$param<-rownames(duration)
colnames(duration)[4:5] <- c("maxCI", "minCI")
duration$maxCI[which(duration$maxCI>1)]<-1
duration$maxCI[which(duration$maxCI< -1)]<- -1
duration$minCI[which(duration$minCI>1)]<-1
duration$minCI[which(duration$minCI< -1)]<- -1


A<- ggplot(size, aes(x=param, y = original)) +
 geom_point(size = 4)+
 geom_errorbar(aes(ymax = maxCI, ymin = minCI))+
 ggtitle("A")+
 xlab("Parameters")+
 ylab ("Partial Rank Correlation Coefficients")+
 scale_x_discrete(labels = c("alpha"=expression(alpha),"beta_h" = expression(beta[b]), "beta_r"=expression(beta[r]),
 "b_h" = expression(b[h]),"d_h" = expression(d[h]), "d_f"=expression(d[f]), "sigma_h"=expression(sigma[b]), "gamma_h" = expression(gamma[b]), "gamma_r" = expression(gamma[r]), "g_h"=expression(g[h]), "g_r"=expression(g[r]), "K_f"=expression(K[f]), "r_f"=expression(r[f])))+
 ylim(-1,1)

B<-ggplot(duration, aes(x=param, y = original)) +
 geom_point(size = 4)+
 geom_errorbar(aes(ymax = maxCI, ymin = minCI))+
 ggtitle("B")+
 xlab("Parameters")+
 ylab (" ")+
 scale_x_discrete(labels = c("alpha"=expression(alpha),"beta_h" = expression(beta[b]), "beta_r"=expression(beta[r]),
 "b_h" = expression(b[h]),"d_h" = expression(d[h]), "d_f"=expression(d[f]), "sigma_h"=expression(sigma[b]), "gamma_h" = expression(gamma[b]), "gamma_r" = expression(gamma[r]), "g_h"=expression(g[h]), "g_r"=expression(g[r]), "K_f"=expression(K[f]), "r_f"=expression(r[f])))+
 ylim(-1,1)

multiplot(A, B, cols=2)


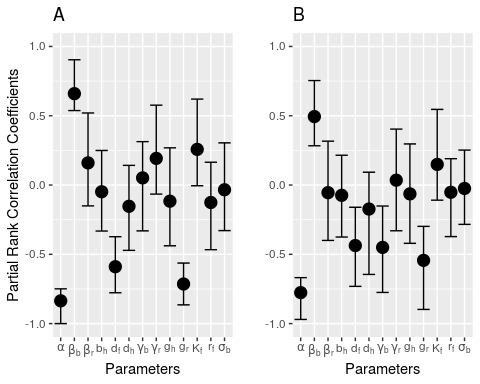


# tiff("FigureS14BubonicSEIR_PRCCuniform.tiff", height =8.7 , width =10, units = "cm", compression = "lzw", res = 1200)
# multiplot(A, B, cols=2)
# dev.off()

## Bubonic SIR with rat carrying capacity and resistance

parameters <- c(r_r=0.014, K_r=499999, p_r=0.975, d_r=0.00055, beta_r = 0.09, alpha=3/500000, gamma_r = 1/5.15, g_r=0.1, r_f=0.0084, K_f=6, d_f=1/5, beta_h=0.19, gamma_h=1/10, g_h=0.34, b_h=1/(25*365), d_h=1/(25*365)) #you can play with transmission and recovery rates here

par(mfrow=c(1,2))
plot(bSIRrK$MaxInf~bSIRrK$r_r, main= expression(paste("Effect of ", r[r], " on Size")), xlab=expression(r[r]), ylab="Outbreak Size")
plot(bSIRrK$Thresh100~bSIRrK$r_r, main= expression(paste("Effect of ", r[r], " on Duration")), xlab=expression(r[r]), ylab="Detectable Duration (days)")


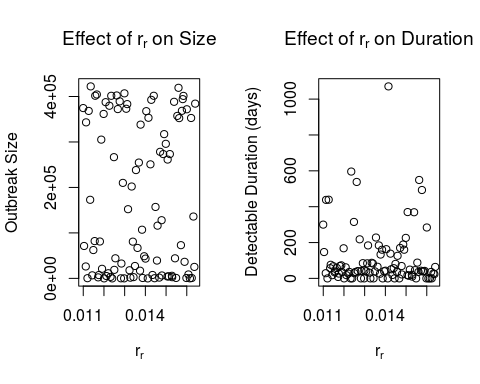


plot(bSIRrK$MaxInf~bSIRrK$K_r, main= expression(paste("Effect of ", K[r], " on Size")), xlab=expression(K[r]), ylab="Outbreak Size")
plot(bSIRrK$Thresh100~bSIRrK$K_r, main= expression(paste("Effect of ", K[r], " on Duration")), xlab=expression(K[r]), ylab="Detectable Duration (days)")


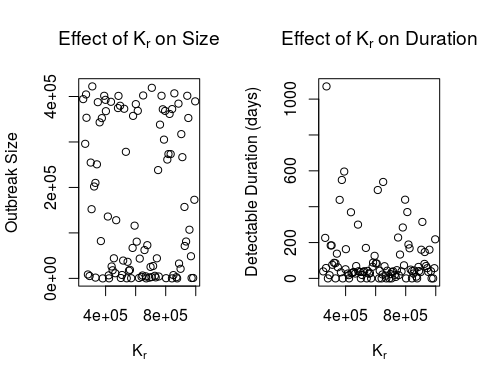


plot(bSIRrK$MaxInf~bSIRrK$p_r, main= expression(paste("Effect of ", p[r], " on Size")), xlab=expression(p[r]), ylab="Outbreak Size")
plot(bSIRrK$Thresh100~bSIRrK$p_r, main= expression(paste("Effect of ", p[r], " on Duration")), xlab=expression(p[r]), ylab="Detectable Duration (days)")


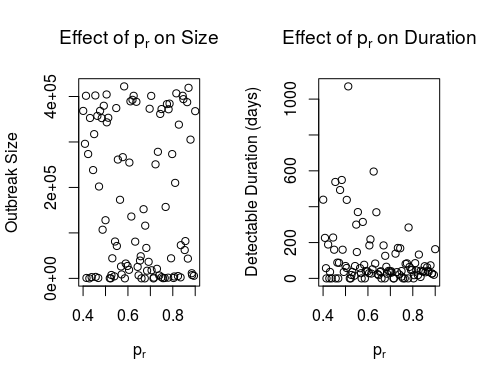


plot(bSIRrK$MaxInf~bSIRrK$d_r, main= expression(paste("Effect of ", d[r], " on Size")), xlab=expression(d[r]), ylab="Outbreak Size")
plot(bSIRrK$Thresh100~bSIRrK$d_r, main= expression(paste("Effect of ", d[r], " on Duration")), xlab=expression(d[r]), ylab="Detectable Duration (days)")


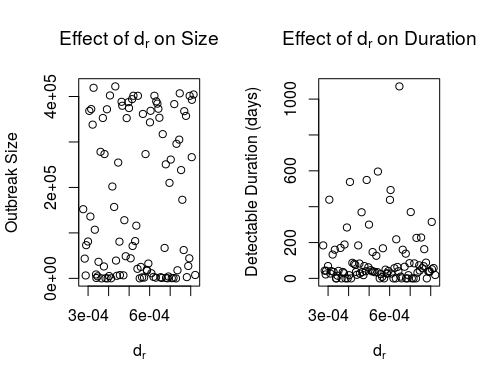


plot(bSIRrK$MaxInf~bSIRrK$beta_r, main= expression(paste("Effect of ", beta[r], " on Size")), xlab=expression(beta[r]), ylab="Outbreak Size")
plot(bSIRrK$Thresh100~bSIRrK$beta_r, main= expression(paste("Effect of ", beta[r], " on Duration")), xlab=expression(beta[r]), ylab="Detectable Duration (days)")


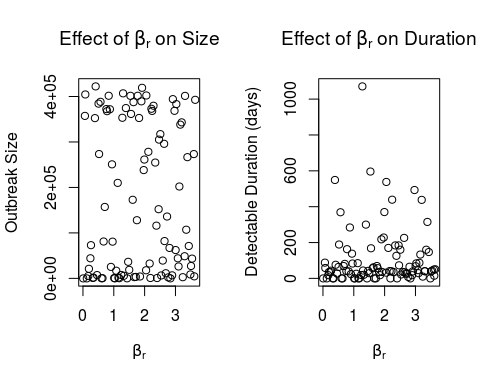


plot(bSIRrK$MaxInf~bSIRrK$alpha, main= expression(paste("Effect of ", alpha, " on Size")), xlab=expression(alpha), ylab="Outbreak Size")
plot(bSIRrK$Thresh100~bSIRrK$alpha, main= expression(paste("Effect of ", alpha, " on Duration")), xlab=expression(alpha), ylab="Detectable Duration (days)")


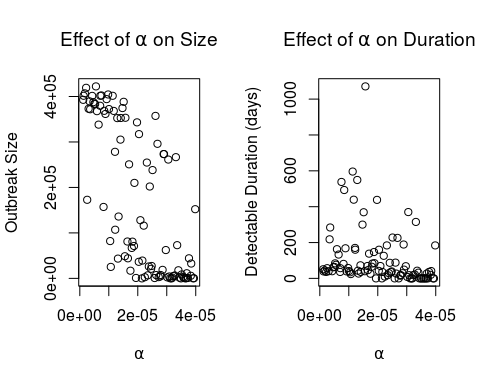


plot(bSIRrK$MaxInf~bSIRrK$gamma_r, main= expression(paste("Effect of ", gamma[r], " on Size")), xlab=expression(gamma[r]), ylab="Outbreak Size")
plot(bSIRrK$Thresh100~bSIRrK$gamma_r, main= expression(paste("Effect of ", gamma[r], " on Duration")), xlab=expression(gamma[r]), ylab="Detectable Duration (days)")


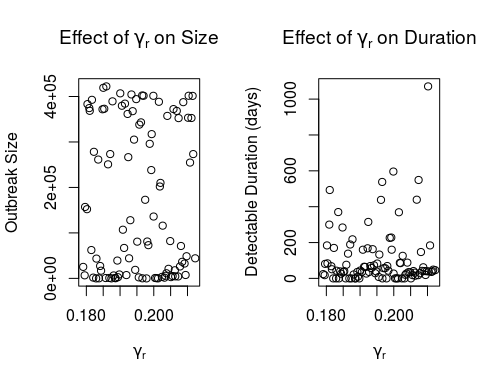


plot(bSIRrK$MaxInf~bSIRrK$g_r, main= expression(paste("Effect of ", g[r], " on Size")), xlab=expression(g[r]), ylab="Outbreak Size")
plot(bSIRrK$Thresh100~bSIRrK$g_r, main= expression(paste("Effect of ", g[r], " on Duration")), xlab=expression(g[r]), ylab="Detectable Duration (days)")


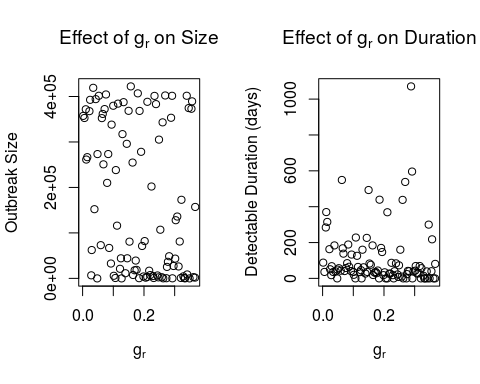


plot(bSIRrK$MaxInf~bSIRrK$r_f, main= expression(paste("Effect of ", r[f], " on Size")), xlab=expression(r[f]), ylab="Outbreak Size")
plot(bSIRrK$Thresh100~bSIRrK$r_f, main= expression(paste("Effect of ", r[f], " on Duration")), xlab=expression(r[f]), ylab="Detectable Duration (days)")


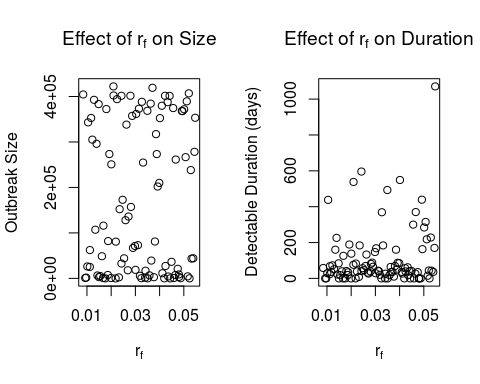


plot(bSIRrK$MaxInf~bSIRrK$K_f, main= expression(paste("Effect of ", K[f], " on Size")), xlab=expression(K[f]), ylab="Outbreak Size")
plot(bSIRrK$Thresh100~bSIRrK$K_f, main= expression(paste("Effect of ", K[f], " on Duration")), xlab=expression(K[f]), ylab="Detectable Duration (days)")


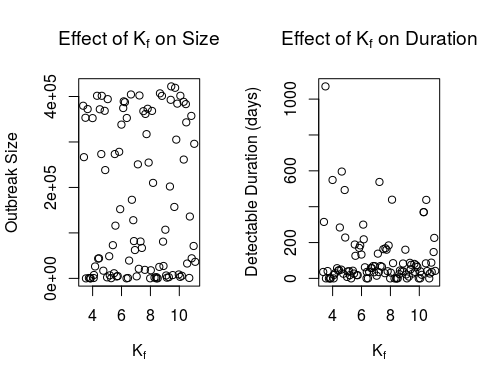


plot(bSIRrK$MaxInf~bSIRrK$d_f, main= expression(paste("Effect of ", d[f], " on Size")), xlab=expression(d[f]), ylab="Outbreak Size")
plot(bSIRrK$Thresh100~bSIRrK$d_f, main= expression(paste("Effect of ", d[f], " on Duration")), xlab=expression(d[f]), ylab="Detectable Duration (days)")


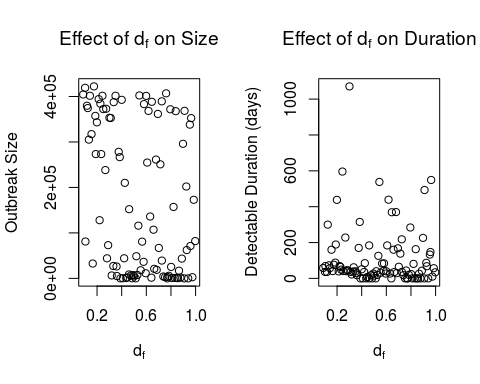


plot(bSIRrK$MaxInf~bSIRrK$beta_h, main= expression(paste("Effect of ", beta[b], " on Size")), xlab=expression(beta[b]), ylab="Outbreak Size")
plot(bSIRrK$Thresh100~bSIRrK$beta_h, main= expression(paste("Effect of ", beta[b], " on Duration")), xlab=expression(beta[b]), ylab="Detectable Duration (days)")


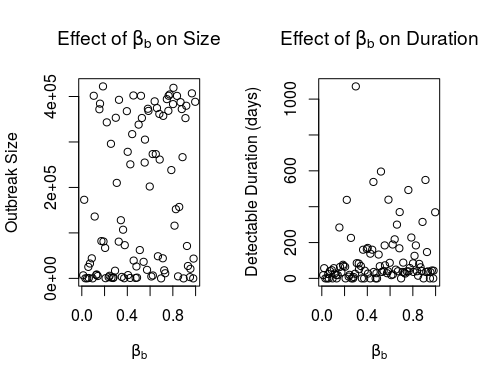


plot(bSIRrK$MaxInf~bSIRrK$gamma_h, main= expression(paste("Effect of ", gamma[b], " on Size")), xlab=expression(gamma[b]), ylab="Outbreak Size")
plot(bSIRrK$Thresh100~bSIRrK$gamma_h, main= expression(paste("Effect of ", gamma[b], " on Duration")), xlab=expression(gamma[b]), ylab="Detectable Duration (days)")


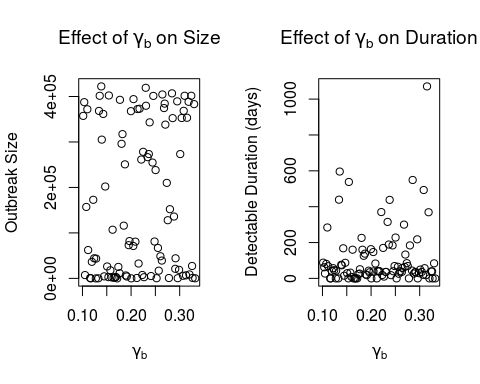


plot(bSIRrK$MaxInf~bSIRrK$g_h, main= expression(paste("Effect of ", g[h], " on Size")), xlab=expression(g[h]), ylab="Outbreak Size")
plot(bSIRrK$Thresh100~bSIRrK$g_h, main= expression(paste("Effect of ", g[h], " on Duration")), xlab=expression(g[h]), ylab="Detectable Duration (days)")


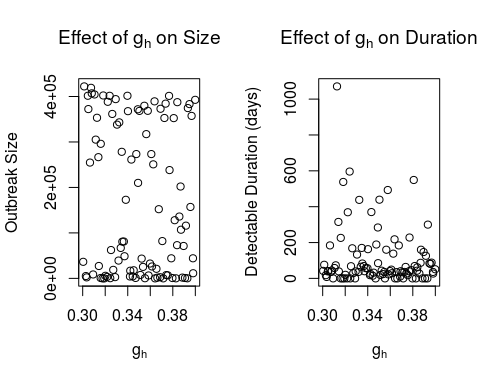


plot(bSIRrK$MaxInf~bSIRrK$b_h, main= expression(paste("Effect of ", b[h], " on Size")), xlab=expression(b[h]), ylab="Outbreak Size")
plot(bSIRrK$Thresh100~bSIRrK$b_h, main= expression(paste("Effect of ", b[h], " on Duration")), xlab=expression(r[r]), ylab="Detectable Duration (days)")


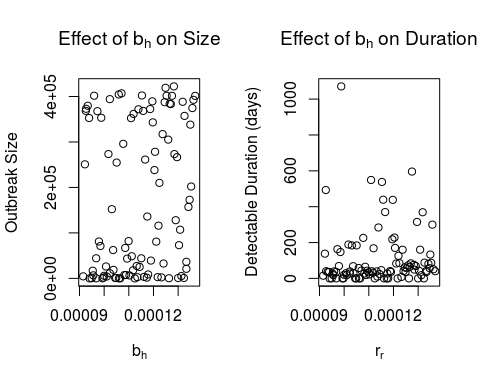


plot(bSIRrK$MaxInf~bSIRrK$d_h, main= expression(paste("Effect of ", d[h], " on Size")), xlab=expression(d[h]), ylab="Outbreak Size")
plot(bSIRrK$Thresh100~bSIRrK$d_h, main= expression(paste("Effect of ", d[h], " on Duration")))


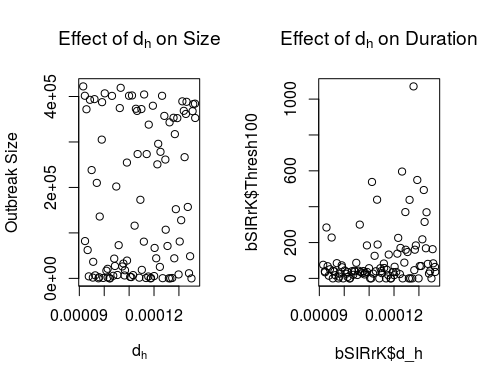


par(mfrow=c(1,2))
boxplot(bSIRrK$MaxInf, main= "Outbreak Size", ylab= "Number of Dead Humans", ylim=c(0,500000))
boxplot(bSIRrK$Thresh100, main= "Outbreak Duration", ylab="Time (Days)")


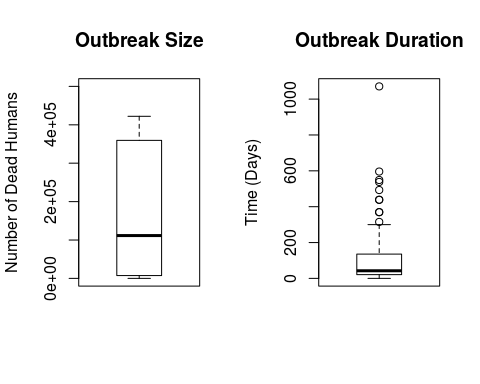


bonferroni.alpha <- 0.05/length(parameters)
prcc_size <- pcc(bSIRrK[,1:length(parameters)], bSIRrK$MaxInf, nboot = niter, rank=TRUE, conf=1-bonferroni.alpha)
prcc_duration <- pcc(bSIRrK[,1:length(parameters)], bSIRrK$Thresh100, nboot = niter, rank=TRUE, conf=1-bonferroni.alpha)

#plot correlation coefficients and confidence intervals for epidemic size and duration
#plot correlation coefficients and confidence intervals for epidemic size and duration
size<-prcc_size$PRCC
size$param<-rownames(size)
colnames(size)[4:5] <- c("maxCI", "minCI")
size$maxCI[which(size$maxCI>1)]<-1
size$maxCI[which(size$maxCI< -1)]<- -1
size$minCI[which(size$minCI>1)]<-1
size$minCI[which(size$minCI< -1)]<- -1

duration<-prcc_duration$PRCC
duration$param<-rownames(duration)
colnames(duration)[4:5] <- c("maxCI", "minCI")
duration$maxCI[which(duration$maxCI>1)]<-1
duration$maxCI[which(duration$maxCI< -1)]<- -1
duration$minCI[which(duration$minCI>1)]<-1
duration$minCI[which(duration$minCI< -1)]<- -1


A<- ggplot(size, aes(x=param, y = original)) +
 geom_point(size = 4)+
 geom_errorbar(aes(ymax = maxCI, ymin = minCI))+
 ggtitle("A")+
 xlab("Parameters")+
 ylab ("Partial Rank Correlation Coefficients")+
 scale_x_discrete(labels = c("r_r"=expression(r[r]), "d_r"=expression(d[r]), "K_r"=expression(K[r]), "p_r"=expression(p[r]), "alpha"=expression(alpha),"beta_h" = expression(beta[b]), "beta_r"=expression(beta[r]),
 "b_h" = expression(b[h]),"d_h" = expression(d[h]), "d_f"=expression(d[f]), "gamma_h" = expression(gamma[b]), "gamma_r" = expression(gamma[r]), "g_h"=expression(g[h]), "g_r"=expression(g[r]), "K_f"=expression(K[f]), "r_f"=expression(r[f])))+
 ylim(-1,1)

B<-ggplot(duration, aes(x=param, y = original)) +
 geom_point(size = 4)+
 geom_errorbar(aes(ymax = maxCI, ymin = minCI))+
 ggtitle("B")+
 xlab("Parameters")+
 ylab (" ")+
 scale_x_discrete(labels = c("r_r"=expression(r[r]), "d_r"=expression(d[r]), "K_r"=expression(K[r]), "p_r"=expression(p[r]), "alpha"=expression(alpha),"beta_h" = expression(beta[b]), "beta_r"=expression(beta[r]),
 "b_h" = expression(b[h]),"d_h" = expression(d[h]), "d_f"=expression(d[f]), "gamma_h" = expression(gamma[b]), "gamma_r" = expression(gamma[r]), "g_h"=expression(g[h]), "g_r"=expression(g[r]), "K_f"=expression(K[f]), "r_f"=expression(r[f])))+
 ylim(-1,1)

multiplot(A, B, cols=2)


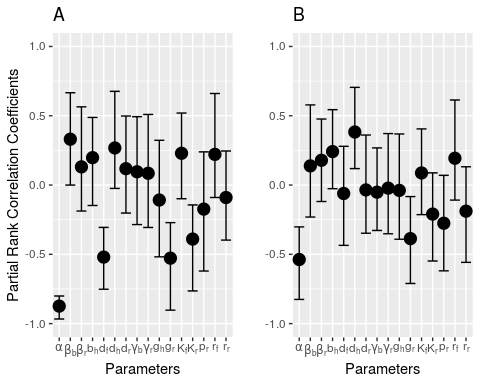


# tiff("FigureS15BubonicSIRrK_PRCCuniform.tiff", height =8.7 , width =10, units = "cm", compression = "lzw", res = 1200)
# multiplot(A, B, cols=2)
# dev.off()

## Bubonic SEIR with rat carrying capacity and resistance

parameters <- c(r_r=0.014, K_r=499999, p_r=0.975, d_r=0.00055, beta_r = 0.09, alpha=3/500000, gamma_r = 1/5.15, g_r=0.1, r_f=0.0084, K_f=6, d_f=1/5, beta_h=0.19, sigma_h= 1/4, gamma_h=1/10, g_h=0.34, b_h=1/(25*365), d_h=1/(25*365)) #you can play with transmission and recovery rates here

par(mfrow=c(1,2))
plot(bSEIRrK$MaxInf~bSEIRrK$r_r, main= expression(paste("Effect of ", r[r], " on Size")), xlab=expression(r[r]), ylab="Outbreak Size")
plot(bSEIRrK$Thresh100~bSEIRrK$r_r, main= expression(paste("Effect of ", r[r], " on Duration")), xlab=expression(r[r]), ylab="Detectable Duration (days)")


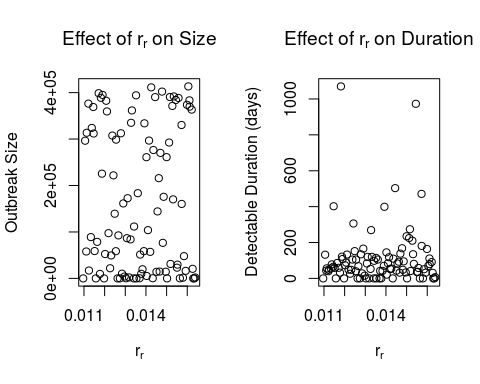


plot(bSEIRrK$MaxInf~bSEIRrK$K_r, main= expression(paste("Effect of ", K[r], " on Size")), xlab=expression(K[r]), ylab="Outbreak Size")
plot(bSEIRrK$Thresh100~bSEIRrK$K_r, main= expression(paste("Effect of ", K[r], " on Duration")), xlab=expression(K[r]), ylab="Detectable Duration (days)")


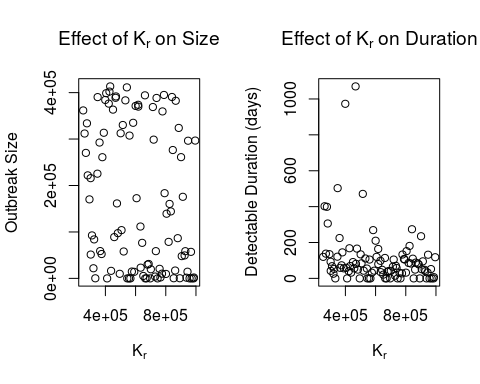


plot(bSEIRrK$MaxInf~bSEIRrK$p_r, main= expression(paste("Effect of ", p[r], " on Size")), xlab=expression(p[r]), ylab="Outbreak Size")
plot(bSEIRrK$Thresh100~bSEIRrK$p_r, main= expression(paste("Effect of ", p[r], " on Duration")), xlab=expression(p[r]), ylab="Detectable Duration (days)")


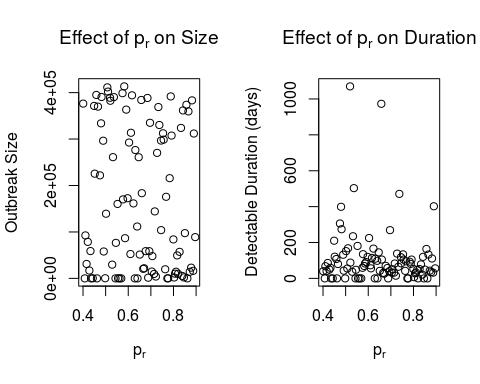


plot(bSEIRrK$MaxInf~bSEIRrK$d_r, main= expression(paste("Effect of ", d[r], " on Size")), xlab=expression(d[r]), ylab="Outbreak Size")
plot(bSEIRrK$Thresh100~bSEIRrK$d_r, main= expression(paste("Effect of ", d[r], " on Duration")), xlab=expression(d[r]), ylab="Detectable Duration (days)")


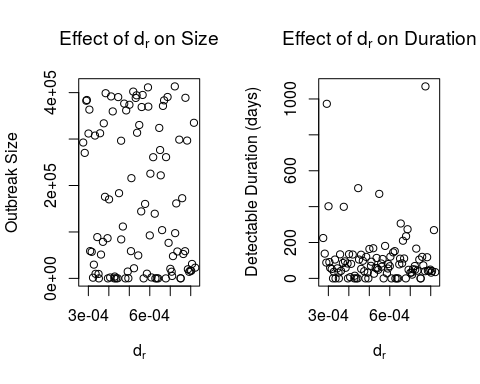


plot(bSEIRrK$MaxInf~bSEIRrK$beta_r, main= expression(paste("Effect of ", beta[r], " on Size")), xlab=expression(beta[r]), ylab="Outbreak Size")
plot(bSEIRrK$Thresh100~bSEIRrK$beta_r, main= expression(paste("Effect of ", beta[r], " on Duration")), xlab=expression(beta[r]), ylab="Detectable Duration (days)")


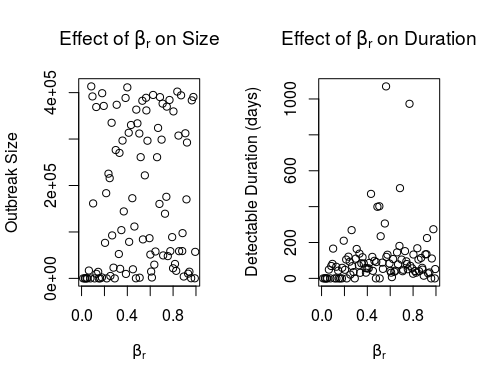


plot(bSEIRrK$MaxInf~bSEIRrK$alpha, main= expression(paste("Effect of ", alpha, " on Size")), xlab=expression(alpha), ylab="Outbreak Size")
plot(bSEIRrK$Thresh100~bSEIRrK$alpha, main= expression(paste("Effect of ", alpha, " on Duration")), xlab=expression(alpha), ylab="Detectable Duration (days)")


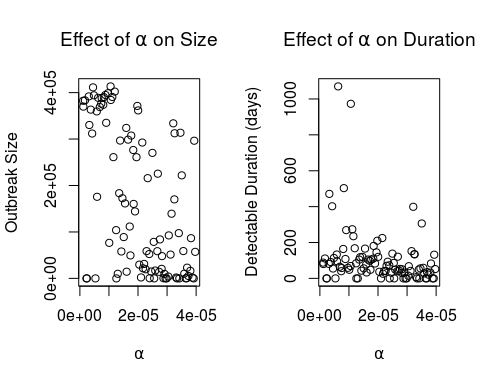


plot(bSEIRrK$MaxInf~bSEIRrK$gamma_r, main= expression(paste("Effect of ", gamma[r], " on Size")), xlab=expression(gamma[r]), ylab="Outbreak Size")
plot(bSEIRrK$Thresh100~bSEIRrK$gamma_r, main= expression(paste("Effect of ", gamma[r], " on Duration")), xlab=expression(gamma[r]), ylab="Detectable Duration (days)")


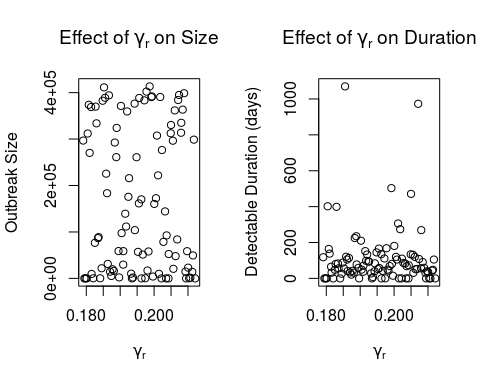


plot(bSEIRrK$MaxInf~bSEIRrK$g_r, main= expression(paste("Effect of ", g[r], " on Size")), xlab=expression(g[r]), ylab="Outbreak Size")
plot(bSEIRrK$Thresh100~bSEIRrK$g_r, main= expression(paste("Effect of ", g[r], " on Duration")), xlab=expression(g[r]), ylab="Detectable Duration (days)")


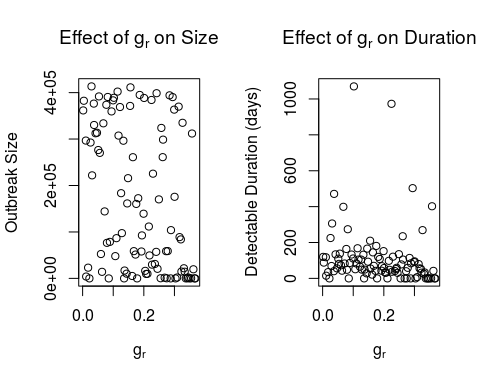


plot(bSEIRrK$MaxInf~bSEIRrK$r_f, main= expression(paste("Effect of ", r[f], " on Size")), xlab=expression(r[f]), ylab="Outbreak Size")
plot(bSEIRrK$Thresh100~bSEIRrK$r_f, main= expression(paste("Effect of ", r[f], " on Duration")), xlab=expression(r[f]), ylab="Detectable Duration (days)")


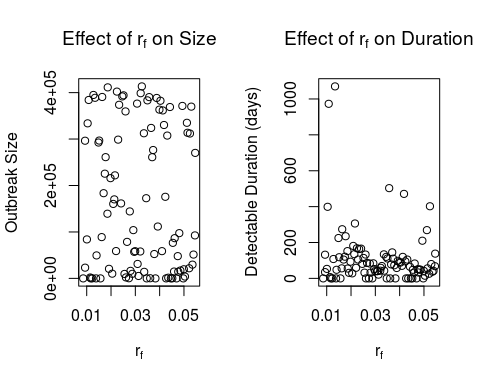


plot(bSEIRrK$MaxInf~bSEIRrK$K_f, main= expression(paste("Effect of ", K[f], " on Size")), xlab=expression(K[f]), ylab="Outbreak Size")
plot(bSEIRrK$Thresh100~bSEIRrK$K_f, main= expression(paste("Effect of ", K[f], " on Duration")), xlab=expression(K[f]), ylab="Detectable Duration (days)")


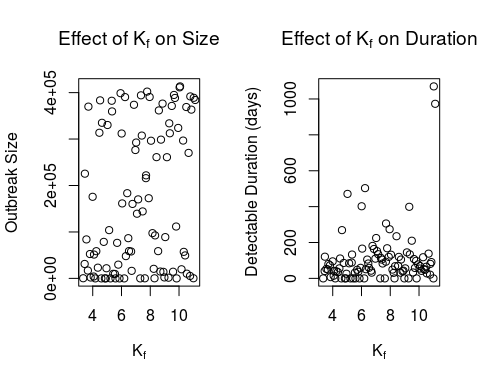


plot(bSEIRrK$MaxInf~bSEIRrK$d_f, main= expression(paste("Effect of ", d[f], " on Size")), xlab=expression(d[f]), ylab="Outbreak Size")
plot(bSEIRrK$Thresh100~bSEIRrK$d_f, main= expression(paste("Effect of ", d[f], " on Duration")), xlab=expression(d[f]), ylab="Detectable Duration (days)")


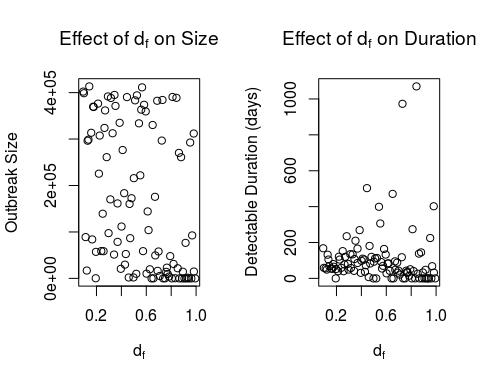


plot(bSEIRrK$MaxInf~bSEIRrK$beta_h, main= expression(paste("Effect of ", beta[b], " on Size")), xlab=expression(beta[b]), ylab="Outbreak Size")
plot(bSEIRrK$Thresh100~bSEIRrK$beta_h, main= expression(paste("Effect of ", beta[b], " on Duration")), xlab=expression(beta[b]), ylab="Detectable Duration (days)")


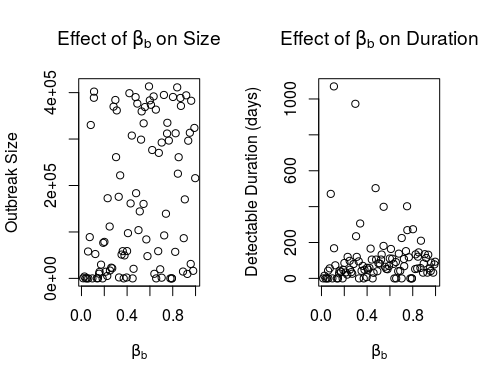


plot(bSEIRrK$MaxInf~bSEIRrK$beta_h, main= expression(paste("Effect of ", beta[b], " on Size")), xlab=expression(sigma[b]), ylab="Outbreak Size")
plot(bSEIRrK$Thresh100~bSEIRrK$beta_h, main= expression(paste("Effect of ", beta[b], " on Duration")), xlab=expression(sigma[b]), ylab="Detectable Duration (days)")


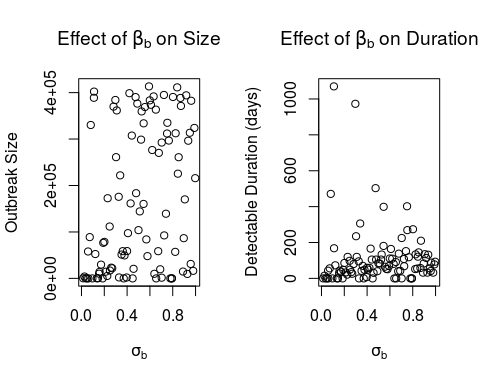


plot(bSEIRrK$MaxInf~bSEIRrK$gamma_h, main= expression(paste("Effect of ", gamma[b], " on Size")), xlab=expression(gamma[b]), ylab="Outbreak Size")
plot(bSEIRrK$Thresh100~bSEIRrK$gamma_h, main= expression(paste("Effect of ", gamma[b], " on Duration")), xlab=expression(gamma[b]), ylab="Detectable Duration (days)")


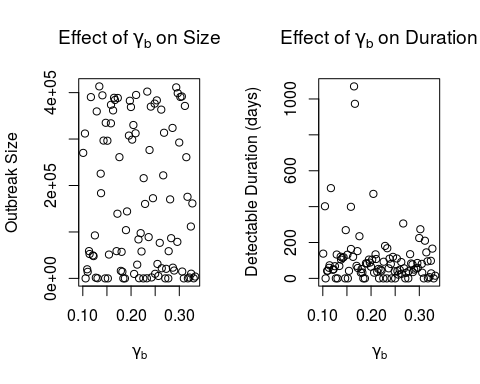


plot(bSEIRrK$MaxInf~bSEIRrK$g_h, main= expression(paste("Effect of ", g[h], " on Size")), xlab=expression(g[h]), ylab="Outbreak Size")
plot(bSEIRrK$Thresh100~bSEIRrK$g_h, main= expression(paste("Effect of ", g[h], " on Duration")), xlab=expression(g[h]), ylab="Detectable Duration (days)")


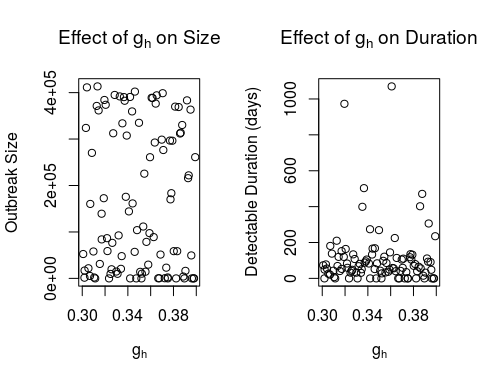


plot(bSEIRrK$MaxInf~bSEIRrK$b_h, main= expression(paste("Effect of ", b[h], " on Size")), xlab=expression(b[h]), ylab="Outbreak Size")
plot(bSEIRrK$Thresh100~bSEIRrK$b_h, main= expression(paste("Effect of ", b[h], " on Duration")), xlab=expression(r[r]), ylab="Detectable Duration (days)")


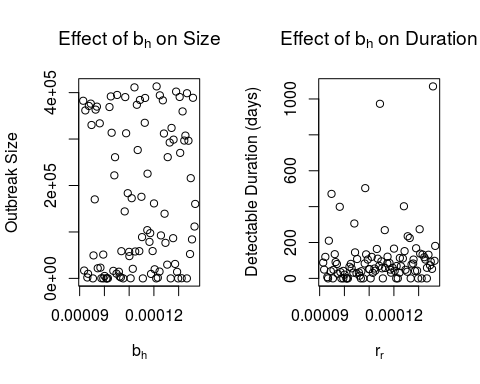


plot(bSEIRrK$MaxInf~bSEIRrK$d_h, main= expression(paste("Effect of ", d[h], " on Size")), xlab=expression(d[h]), ylab="Outbreak Size")
plot(bSEIRrK$Thresh100~bSEIRrK$d_h, main= expression(paste("Effect of ", d[h], " on Duration")), xlab=expression(d[h]), ylab="Detectable Duration (days)")


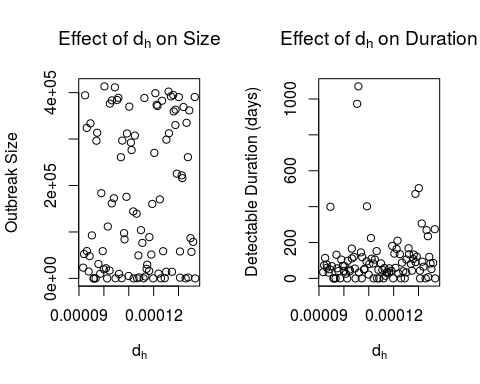


par(mfrow=c(1,2))
boxplot(bSEIRrK$MaxInf, main= "Outbreak Size", ylab= "Number of Dead Humans", ylim=c(0,500000))
boxplot(bSEIRrK$Thresh100, main= "Outbreak Duration", ylab="Time (Days)")


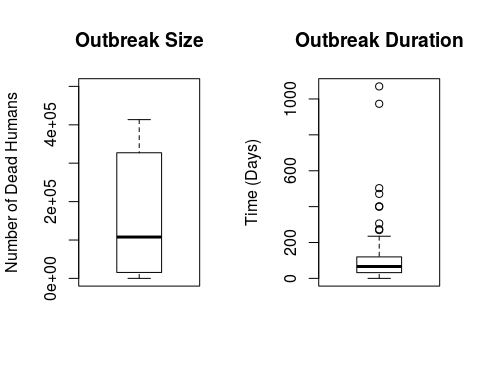


bonferroni.alpha <- 0.05/length(parameters)
prcc_size <- pcc(bSEIRrK[,1:length(parameters)], bSEIRrK$MaxInf, nboot = niter, rank=TRUE, conf=1-bonferroni.alpha)
prcc_duration <- pcc(bSEIRrK[,1:length(parameters)], bSEIRrK$Thresh100, nboot = niter, rank=TRUE, conf=1-bonferroni.alpha)

#plot correlation coefficients and confidence intervals for epidemic size and duration
size<-prcc_size$PRCC
size$param<-rownames(size)
colnames(size)[4:5] <- c("maxCI", "minCI")
size$maxCI[which(size$maxCI>1)]<-1
size$maxCI[which(size$maxCI< -1)]<- -1
size$minCI[which(size$minCI>1)]<-1
size$minCI[which(size$minCI< -1)]<- -1

duration<-prcc_duration$PRCC
duration$param<-rownames(duration)
colnames(duration)[4:5] <- c("maxCI", "minCI")
duration$maxCI[which(duration$maxCI>1)]<-1
duration$maxCI[which(duration$maxCI< -1)]<- -1
duration$minCI[which(duration$minCI>1)]<-1
duration$minCI[which(duration$minCI< -1)]<- -1


A<- ggplot(size, aes(x=param, y = original)) +
 geom_point(size = 4)+
 geom_errorbar(aes(ymax = maxCI, ymin = minCI))+
 ggtitle("A")+
 xlab("Parameters")+
 ylab ("Partial Rank Correlation Coefficients")+
 scale_x_discrete(labels = c("r_r"=expression(r[r]), "d_r"=expression(d[r]), "K_r"=expression(K[r]), "p_r"=expression(p[r]), "alpha"=expression(alpha),"beta_h" = expression(beta[b]), "beta_r"=expression(beta[r]), "sigma_h"=expression(sigma[b]),
 "b_h" = expression(b[h]),"d_h" = expression(d[h]), "d_f"=expression(d[f]), "gamma_h" = expression(gamma[b]), "gamma_r" = expression(gamma[r]), "g_h"=expression(g[h]), "g_r"=expression(g[r]), "K_f"=expression(K[f]), "r_f"=expression(r[f])))+
 ylim(-1,1)

B<-ggplot(duration, aes(x=param, y = original)) +
 geom_point(size = 4)+
 geom_errorbar(aes(ymax = maxCI, ymin = minCI))+
 ggtitle("B")+
 xlab("Parameters")+
 ylab (" ")+
 scale_x_discrete(labels = c("r_r"=expression(r[r]), "d_r"=expression(d[r]), "K_r"=expression(K[r]), "p_r"=expression(p[r]), "alpha"=expression(alpha),"beta_h" = expression(beta[b]), "beta_r"=expression(beta[r]), "sigma_h"=expression(sigma[b]),
 "b_h" = expression(b[h]),"d_h" = expression(d[h]), "d_f"=expression(d[f]), "gamma_h" = expression(gamma[b]), "gamma_r" = expression(gamma[r]), "g_h"=expression(g[h]), "g_r"=expression(g[r]), "K_f"=expression(K[f]), "r_f"=expression(r[f])))+
 ylim(-1,1)

multiplot(A, B, cols=2)


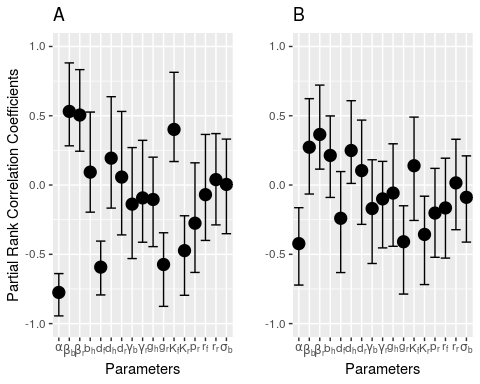


# tiff("FigureS16BubonicSEIRrK_PRCCuniform.tiff", height =8.7 , width =10, units = "cm", compression = "lzw", res = 1200)
# multiplot(A, B, cols=2)
# dev.off()

## Bubonic/Pneumonic SEIR

parameters <- c(beta_r = 0.09, alpha=3/500000, gamma_r = 1/5.15, g_r=0.1, r_f=0.0084, K_f=6, d_f=1/5, beta_b=0.19, beta_p = 0.45, sigma_b= 1/6, sigma_p=1/4.3, gamma_b=1/10, gamma_p=1/2.5, p=0.2, g_h=0.34, b_h=1/(25*365), d_h=1/(25*365)) #you can play with transmission and recovery rates here

par(mfrow=c(1,2))
plot(bpSEIR$MaxInf~bpSEIR$beta_r, main= expression(paste("Effect of ", beta[r], " on Size")), xlab=expression(beta[r]), ylab="Outbreak Size")
plot(bpSEIR$Thresh100~bpSEIR$beta_r, main= expression(paste("Effect of ", beta[r], " on Duration")), xlab=expression(beta[r]), ylab="Detectable Duration (days)")


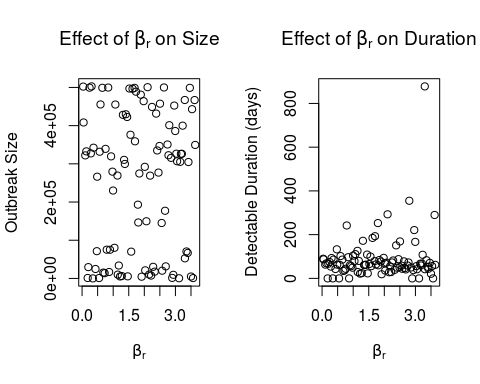


plot(bpSEIR$MaxInf~bpSEIR$alpha, main= expression(paste("Effect of ", alpha, " on Size")), xlab=expression(alpha), ylab="Outbreak Size")
plot(bpSEIR$Thresh100~bpSEIR$alpha, main= expression(paste("Effect of ", alpha, " on Duration")), xlab=expression(alpha), ylab="Detectable Duration (days)")


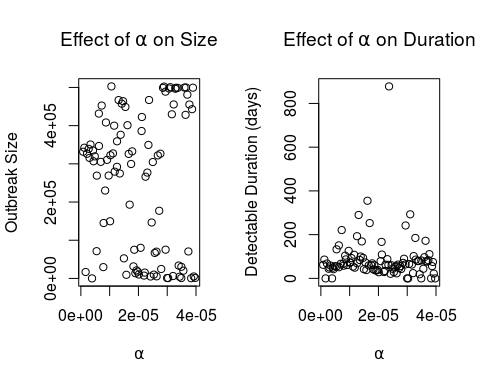


plot(bpSEIR$MaxInf~bpSEIR$gamma_r, main= expression(paste("Effect of ", gamma[r], " on Size")), xlab=expression(gamma[r]), ylab="Outbreak Size")
plot(bpSEIR$Thresh100~bpSEIR$gamma_r, main= expression(paste("Effect of ", gamma[r], " on Duration")), xlab=expression(gamma[r]), ylab="Detectable Duration (days)")


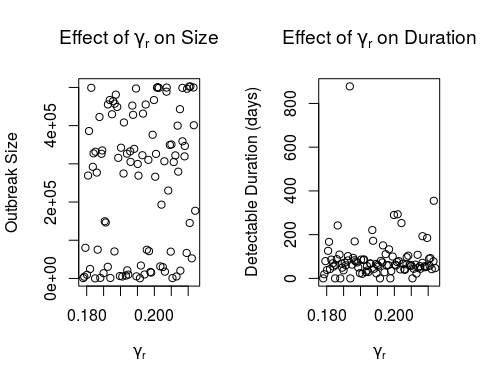


plot(bpSEIR$MaxInf~bpSEIR$g_r, main= expression(paste("Effect of ", g[r], " on Size")), xlab=expression(g[r]), ylab="Outbreak Size")
plot(bpSEIR$Thresh100~bpSEIR$g_r, main= expression(paste("Effect of ", g[r], " on Duration")), xlab=expression(g[r]), ylab="Detectable Duration (days)")


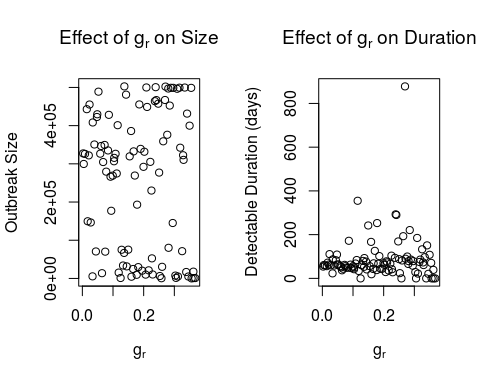


plot(bpSEIR$MaxInf~bpSEIR$r_f, main= expression(paste("Effect of ", r[f], " on Size")), xlab=expression(r[f]), ylab="Outbreak Size")
plot(bpSEIR$Thresh100~bpSEIR$r_f, main= expression(paste("Effect of ", r[f], " on Duration")), xlab=expression(r[f]), ylab="Detectable Duration (days)")


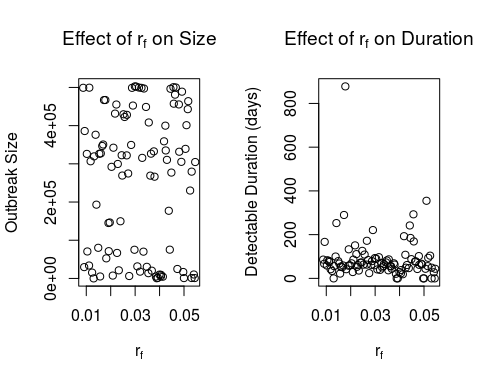


plot(bpSEIR$MaxInf~bpSEIR$K_f, main= expression(paste("Effect of ", K[f], " on Size")), xlab=expression(K[f]), ylab="Outbreak Size")
plot(bpSEIR$Thresh100~bpSEIR$K_f, main= expression(paste("Effect of ", K[f], " on Duration")), xlab=expression(K[f]), ylab="Detectable Duration (days)")


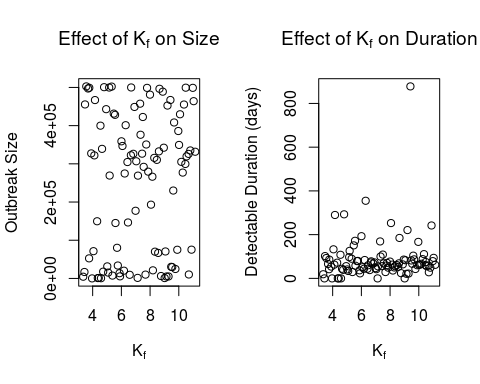


plot(bpSEIR$MaxInf~bpSEIR$d_f, main= expression(paste("Effect of ", d[f], " on Size")), xlab=expression(d[f]), ylab="Outbreak Size")
plot(bpSEIR$Thresh100~bpSEIR$d_f, main= expression(paste("Effect of ", d[f], " on Duration")), xlab=expression(d[f]), ylab="Detectable Duration (days)")


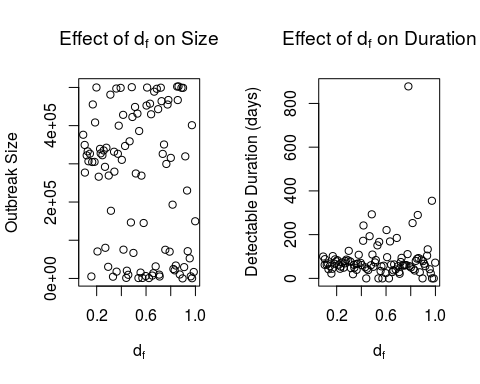


plot(bpSEIR$MaxInf~bpSEIR$beta_b, main= expression(paste("Effect of ", beta[b], " on Size")), xlab=expression(beta[b]), ylab="Outbreak Size")
plot(bpSEIR$Thresh100~bpSEIR$beta_b, main= expression(paste("Effect of ", beta[b], " on Duration")), xlab=expression(beta[b]), ylab="Detectable Duration (days)")


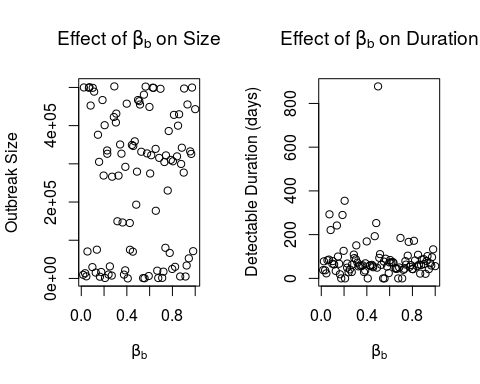


plot(bpSEIR$MaxInf~bpSEIR$sigma_b, main= expression(paste("Effect of ", sigma[b], " on Size")), xlab=expression(sigma[b]), ylab="Outbreak Size")
plot(bpSEIR$Thresh100~bpSEIR$sigma_b, main= expression(paste("Effect of ", sigma[b], " on Duration")), xlab=expression(sigma[b]), ylab="Detectable Duration (days)")


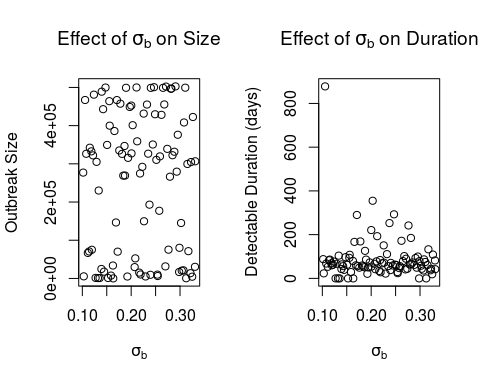


plot(bpSEIR$MaxInf~bpSEIR$gamma_b, main= expression(paste("Effect of ", gamma[b], " on Size")), xlab=expression(gamma[b]), ylab="Outbreak Size")
plot(bpSEIR$Thresh100~bpSEIR$gamma_b, main= expression(paste("Effect of ", gamma[b], " on Duration")), xlab=expression(gamma[b]), ylab="Detectable Duration (days)")


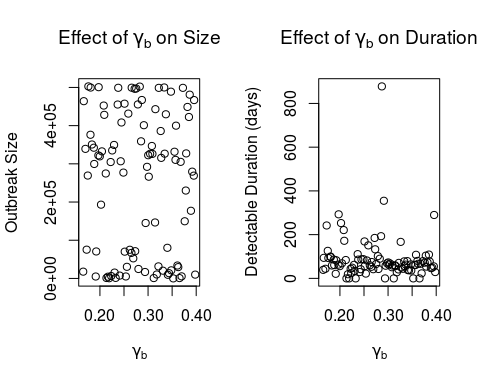


plot(bpSEIR$MaxInf~bpSEIR$beta_p, main= expression(paste("Effect of ", beta[p], " on Size")), xlab=expression(beta[p]), ylab="Outbreak Size")
plot(bpSEIR$Thresh100~bpSEIR$beta_p, main= expression(paste("Effect of ", beta[p], " on Duration")), xlab=expression(beta[p]), ylab="Detectable Duration (days)")


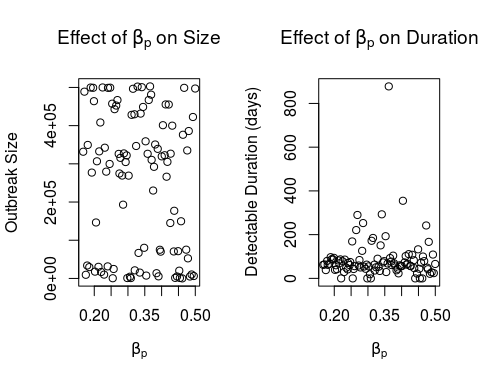


plot(bpSEIR$MaxInf~bpSEIR$sigma_p, main= expression(paste("Effect of ", sigma[p], " on Size")), xlab=expression(sigma[p]), ylab="Outbreak Size")
plot(bpSEIR$Thresh100~bpSEIR$sigma_p, main= expression(paste("Effect of ", sigma[p], " on Duration")), xlab=expression(sigma[p]), ylab="Detectable Duration (days)")


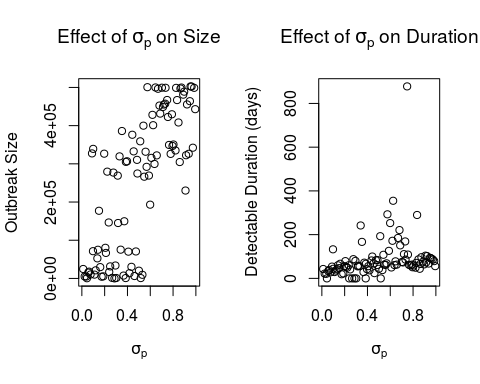


plot(bpSEIR$MaxInf~bpSEIR$gamma_p, main= expression(paste("Effect of ", gamma[p], " on Size")), xlab=expression(gamma[p]), ylab="Outbreak Size")
plot(bpSEIR$Thresh100~bpSEIR$gamma_p, main= expression(paste("Effect of ", gamma[p], " on Duration")), xlab=expression(gamma[p]), ylab="Detectable Duration (days)")


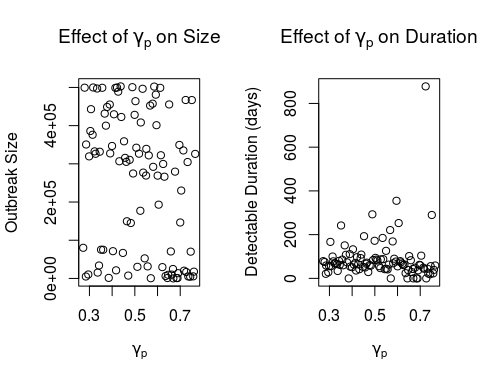


plot(bpSEIR$MaxInf~bpSEIR$g_h, main= expression(paste("Effect of ", g[h], " on Size")), xlab=expression(g[h]), ylab="Outbreak Size")
plot(bpSEIR$Thresh100~bpSEIR$g_h, main= expression(paste("Effect of ", g[h], " on Duration")), xlab=expression(g[h]), ylab="Detectable Duration (days)")


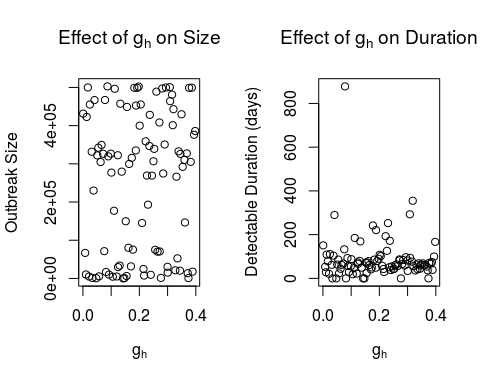


plot(bpSEIR$MaxInf~bpSEIR$p, main= expression(paste("Effect of ", p, " on Size")), xlab="p", ylab="Outbreak Size")
plot(bpSEIR$Thresh100~bpSEIR$p, main= expression(paste("Effect of ", p, " on Duration")), xlab="p", ylab="Detectable Duration (days)")


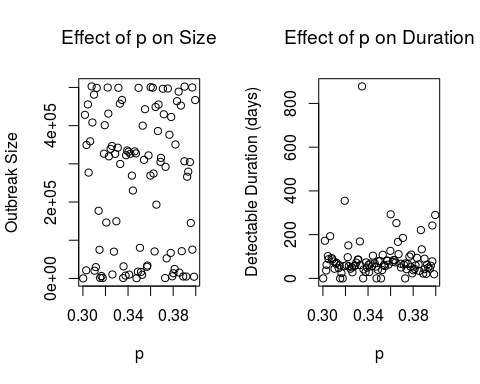


plot(bpSEIR$MaxInf~bpSEIR$b_h, main= expression(paste("Effect of ", b[h], " on Size")), xlab=expression(b[h]), ylab="Outbreak Size")
plot(bpSEIR$Thresh100~bpSEIR$b_h, main= expression(paste("Effect of ", b[h], " on Duration")), xlab=expression(b[h]), ylab="Detectable Duration (days)")


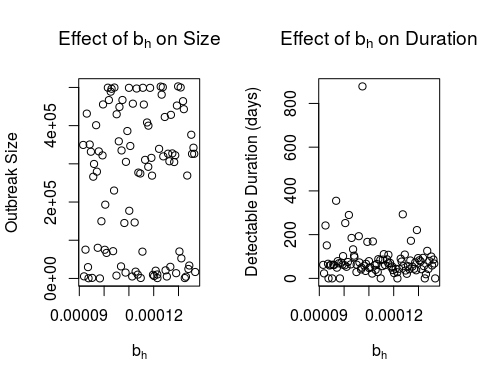


plot(bpSEIR$MaxInf~bpSEIR$d_h, main= expression(paste("Effect of ", d[h], " on Size")), xlab=expression(d[h]), ylab="Outbreak Size")
plot(bpSEIR$Thresh100~bpSEIR$d_h, main= expression(paste("Effect of ", d[h], " on Duration")), xlab=expression(d[h]), ylab="Detectable Duration (days)")


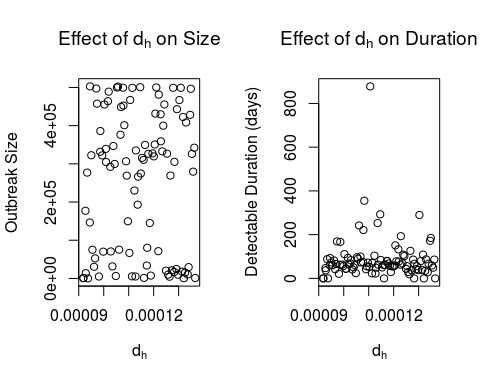


par(mfrow=c(1,2))
boxplot(bpSEIR$MaxInf, main= "Outbreak Size", ylab= "Number of Dead Humans", ylim=c(0,500000))
boxplot(bpSEIR$Thresh100, main= "Outbreak Duration", ylab="Time (Days)")


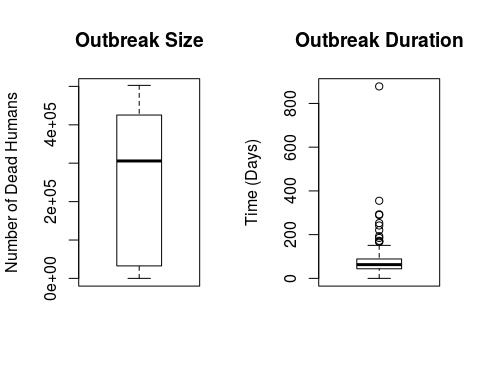


bonferroni.alpha <- 0.05/length(parameters)
prcc_size <- pcc(bpSEIR[,1:length(parameters)], bpSEIR[,length(parameters)+1], nboot = niter, rank=TRUE, conf=1-bonferroni.alpha)
prcc_duration <- pcc(bpSEIR[,1:length(parameters)], bpSEIR[,length(parameters)+2], nboot = niter, rank=TRUE, conf=1-bonferroni.alpha)

#plot correlation coefficients and confidence intervals for epidemic size and duration
size<-prcc_size$PRCC
size$param<-rownames(size)
colnames(size)[4:5] <- c("maxCI", "minCI")
size$maxCI[which(size$maxCI>1)]<-1
size$maxCI[which(size$maxCI< -1)]<- -1
size$minCI[which(size$minCI>1)]<-1
size$minCI[which(size$minCI< -1)]<- -1

duration<-prcc_duration$PRCC
duration$param<-rownames(duration)
colnames(duration)[4:5] <- c("maxCI", "minCI")
duration$maxCI[which(duration$maxCI>1)]<-1
duration$maxCI[which(duration$maxCI< -1)]<- -1
duration$minCI[which(duration$minCI>1)]<-1
duration$minCI[which(duration$minCI< -1)]<- -1


A<- ggplot(size, aes(x=param, y = original)) +
 geom_point(size = 4)+
 geom_errorbar(aes(ymax = maxCI, ymin = minCI))+
 ggtitle("A")+
 xlab("Parameters")+
 ylab ("Partial Rank Correlation Coefficients")+
 scale_x_discrete(labels = c("alpha"=expression(alpha),"beta_b" = expression(beta[b]), "beta_p" = expression(beta[p]),"beta_r"=expression(beta[r]),
 "b_h" = expression(b[h]),"d_h" = expression(d[h]), "d_f"=expression(d[f]), "sigma_b"=expression(sigma[b]), "gamma_b" = expression(gamma[b]), "sigma_p"=expression(sigma[p]), "gamma_p"=expression(gamma[p]), "gamma_r" = expression(gamma[r]), "g_h"=expression(g[h]), "g_r"=expression(g[r]), "K_f"=expression(K[f]), "r_f"=expression(r[f])))+
 ylim(-1 ,1)


B<-ggplot(duration, aes(x=param, y = original)) +
 geom_point(size = 4)+
 geom_errorbar(aes(ymax = maxCI, ymin = minCI))+
 ggtitle("B")+
 xlab("Parameters")+
 ylab (" ")+
 scale_x_discrete(labels = c("alpha"=expression(alpha),"beta_b" = expression(beta[b]), "beta_p" = expression(beta[p]),"beta_r"=expression(beta[r]),
 "b_h" = expression(b[h]),"d_h" = expression(d[h]), "d_f"=expression(d[f]), "sigma_b"=expression(sigma[b]), "gamma_b" = expression(gamma[b]), "sigma_p"=expression(sigma[p]), "gamma_p"=expression(gamma[p]), "gamma_r" = expression(gamma[r]), "g_h"=expression(g[h]), "g_r"=expression(g[r]), "K_f"=expression(K[f]), "r_f"=expression(r[f])))+
 ylim(-1 ,1)

multiplot(A, B, cols=2)


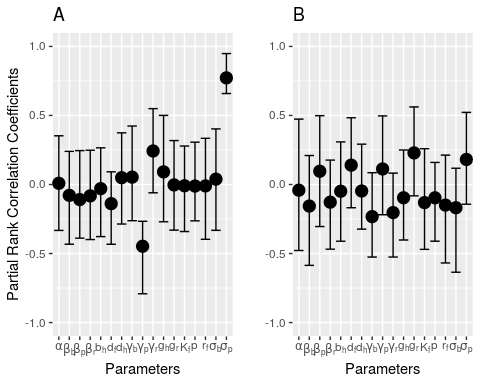


# tiff("FigureS17bpSEIR_PRCCuniform.tiff", height =8.7 , width =10, units = "cm", compression = "lzw", res = 1200)
# multiplot(A, B, cols=2)
# dev.off()
