# Supplementary material for: Modeling the Justinianic Plague: Comparing hypothesized transmission routes
Source: PLoS One. 2020 Apr 30;15(4):e0231256. doi: 10.1371/journal.pone.0231256 (PMC7192389; doi:10.1371/journal.pone.0231256)
Supplement: S2 Appendix — Includes individual scatter plots of parameters vs. model outcomes and PRCC plots. (DOCX) [file pone.0231256.s011.docx]

S2. File: Non Uniform Sensitivity Analysis

Lauren White

9/1/2019

# Sensitivity Analysis with Non-Uniform Parameter Distributions

- Begin by creating LHS sampling space with LHSnonuniform.R

#' Adapted from: https://daphnia.ecology.uga.edu/drakelab/wp-content/uploads/2015/07/sensitivity-ebola.pdf
#' load ode functions for each of the models
source('~/JustinianPlague/Plague_model_functions.R')

require(lhs) #add the lhs library
library(sensitivity)
require(ggplot2)
library(tidyverse)
library(ggforce)
require(deSolve)

set.seed(2718) #set random seed
times <- seq(0, 5000, by= 1) #time sequence to integrate over for all models
h <- 100 #choose number of parameter sets/subdivisions to sample to sample
N_r0<-500000 #initial conditions for ODE model- number of rats
niter<-500 #number of times to bootstrap CI for PRCC

#' load uniform and non uniform LHS distributions
source('~/JustinianPlague/LHSnonuniform.R')
uniform<-FALSE #choose uniform (TRUE) or non-uniform (FALSE) distributions

# Latin Hypercube Sampling and Partial Ranked Correlation Coefficients (LHS-PRCC)

- Use GlobalSensitivityAnalysis.R to run ODE models on LHS parameter sets produced in LHSnonuniform.R
- Plot scatter plots of each parameter vs. outbreak size and dectable outbreak duration (days)
- Calculate and plot PRCC values for each parameter for outbreak size and detectable outbreak duration (days)

source('~/JustinianPlague/GlobalSensitivityAnalysis.R')

## Define multiplot function

Use to produce multipaneled ggplot2 figures

#' Multiplot function
# ggplot objects can be passed in ..., or to plotlist (as a list of ggplot objects)
# - cols: Number of columns in layout
# - layout: A matrix specifying the layout. If present, 'cols' is ignored.
#
# If the layout is something like matrix(c(1,2,3,3), nrow=2, byrow=TRUE),
# then plot 1 will go in the upper left, 2 will go in the upper right, and
# 3 will go all the way across the bottom.
#
multiplot <- function(..., plotlist=NULL, file, cols=1, layout=NULL) {
 library(grid)

 # Make a list from the ... arguments and plotlist
 plots <- c(list(...), plotlist)

 numPlots = length(plots)

 # If layout is NULL, then use 'cols' to determine layout
 if (is.null(layout)) {
 # Make the panel
 # ncol: Number of columns of plots
 # nrow: Number of rows needed, calculated from # of cols
 layout <- matrix(seq(1, cols * ceiling(numPlots/cols)),
 ncol = cols, nrow = ceiling(numPlots/cols), byrow=TRUE)
 }

 if (numPlots==1) {
 print(plots[[1]])

 } else {
 # Set up the page
 grid.newpage()
 pushViewport(viewport(layout = grid.layout(nrow(layout), ncol(layout))))

 # Make each plot, in the correct location
 for (i in 1:numPlots) {
 # Get the i,j matrix positions of the regions that contain this subplot
 matchidx <- as.data.frame(which(layout == i, arr.ind = TRUE))

 print(plots[[i]], vp = viewport(layout.pos.row = matchidx$row,
 layout.pos.col = matchidx$col))
 }
 }
}

## Comparative Figure Across Models

# Comparative Figure for All Models ---------------------------------------

comp_size<-data.frame(pSIR=pSIR$MaxInf, pSEIR= pSEIR$MaxInf, bSIR=bSIR$MaxInf, bSEIR= bSEIR$MaxInf, bSIRrK=bSIRrK$MaxInf, bSEIRrK=bSEIRrK$MaxInf, bpSEIR= bpSEIR$MaxInf) #, eSIR=eSIR$MaxInf)
comp_dur<- data.frame(pSIR=pSIR$Thresh100, pSEIR= pSEIR$Thresh100, bSIR=bSIR$Thresh100, bSEIR= bSEIR$Thresh100, bSIRrK=bSIRrK$Thresh100, bSEIRrK=bSEIRrK$Thresh100, bpSEIR= bpSEIR$Thresh100) #, eSIR=eSIR$Thresh100)
comp_dur250<- data.frame(pSIR=pSIR$Thresh250, pSEIR= pSEIR$Thresh250, bSIR=bSIR$Thresh250, bSEIR= bSEIR$Thresh250, bSIRrK=bSIRrK$Thresh250, bSEIRrK=bSEIRrK$Thresh250, bpSEIR= bpSEIR$Thresh250) #, eSIR=eSIR$Thresh250)


long_DFsize <- comp_size %>% gather(Model, NumberDead, c(pSIR, pSEIR, bSIR, bSEIR, bSIRrK, bSEIRrK, bpSEIR))

long_DFdur <- comp_dur %>% gather(Model, Duration, c(pSIR, pSEIR, bSIR, bSEIR, bSIRrK, bSEIRrK, bpSEIR))

long_DFdur250 <- comp_dur250 %>% gather(Model, Duration, c(pSIR, pSEIR, bSIR, bSEIR, bSIRrK, bSEIRrK, bpSEIR))

long_DFsize$Model <- as.character(long_DFsize$Model)
long_DFsize$Model <- factor(long_DFsize$Model, levels=c("pSIR", "pSEIR", "bSIR", "bSEIR", "bSIRrK", "bSEIRrK", "bpSEIR"))

long_DFdur$Model <- as.character(long_DFdur$Model)
long_DFdur$Model <- factor(long_DFdur$Model, levels=c("pSIR", "pSEIR", "bSIR", "bSEIR", "bSIRrK", "bSEIRrK", "bpSEIR"))

long_DFdur250$Model <- as.character(long_DFdur250$Model)
long_DFdur250$Model <- factor(long_DFdur250$Model, levels=c("pSIR", "pSEIR", "bSIR", "bSEIR", "bSIRrK", "bSEIRrK", "bpSEIR"))

A<- ggplot(long_DFsize, aes(Model, NumberDead)) + geom_boxplot()+ geom_jitter(alpha=0.5) +
 ylab("Number of Human\nMortalities")+ xlab("")+
 scale_x_discrete(labels = c(pSIR="Pneumonic\nSIR", pSEIR="Pneumonic\nSEIR", bSIR="Bubonic\nSIR", bSEIR="Bubonic\nSEIR", bSIRrK="Bubonic SIR\n(Rat Dyn.)", bpSEIR="Bubonic &\nPneumonic\nSEIR", bSEIRrK="Bubonic SEIR\n(Rat Dyn.)"))+
 geom_hline(yintercept = 250000, color="red")+
 ggtitle("A")+
 theme_bw() +
 theme(panel.border = element_blank(), panel.grid.major = element_blank(),panel.grid.minor = element_blank(), axis.line = element_line(colour = "black"), axis.text.x = element_text(angle = 90, hjust = 1, vjust=0.5))

B<- ggplot(long_DFdur, aes(Model, Duration)) + geom_boxplot()+ geom_jitter(alpha=0.5) +
 ylab("Detectable Duration\n (>100 Deaths/Day) (Days)")+ xlab("")+
 scale_x_discrete(labels = c(pSIR="Pneumonic\nSIR", pSEIR="Pneumonic\nSEIR", bSIR="Bubonic\nSIR", bSEIR="Bubonic\nSEIR", bSIRrK="Bubonic SIR\n(Rat Dyn.)", bpSEIR="Bubonic &\nPneumonic\nSEIR", bSEIRrK="Bubonic SEIR\n(Rat Dyn.)"))+
 theme(axis.text.x = element_text(angle = 90, hjust = 1))+
 geom_hline(yintercept = 120, color="red")+
 ggtitle("B")+
 theme_bw() +
 theme(panel.border = element_blank(), panel.grid.major = element_blank(),panel.grid.minor = element_blank(), axis.line = element_line(colour = "black"), axis.text.x = element_text(angle = 90, hjust = 1, vjust=0.5))+
 facet_zoom(ylim = c(0, 300))

C<- ggplot(long_DFdur250, aes(Model, Duration)) + geom_boxplot()+ geom_jitter(alpha=0.5) +
 ylab("Detectable Duration\n (>250 Deaths/Day) (Days)")+ xlab("")+
 scale_x_discrete(labels = c(pSIR="Pneumonic\nSIR", pSEIR="Pneumonic\nSEIR", bSIR="Bubonic\nSIR", bSEIR="Bubonic\nSEIR", bSIRrK="Bubonic SIR\n(Rat Dyn.)", bpSEIR="Bubonic &\nPneumonic\nSEIR", bSEIRrK="Bubonic SEIR\n(Rat Dyn.)"))+
 theme(axis.text.x = element_text(angle = 90, hjust = 1))+
 geom_hline(yintercept = 90, color="red")+
 ggtitle("C")+
 theme_bw() +
 theme(panel.border = element_blank(), panel.grid.major = element_blank(),panel.grid.minor = element_blank(), axis.line = element_line(colour = "black"), axis.text.x = element_text(angle = 90, hjust = 1, vjust=0.5))+
 facet_zoom(ylim = c(0, 250))

# multiplot(A, B, C, cols=1)

tiff("Fig2.tiff", height =22.23 , width =19.05, units = "cm", compression = "lzw", res = 600)
multiplot(A, B, C, cols=1)
dev.off()

## png
## 2

## LHS-PRCC Results

For the pneumonic SIR model, transmission rate $(\beta_{p})$ and human birth $(b_{h})$ rate were positively correlated with outbreak size (Fig. S4A). Natural human death rate $(d_{h})$ was negatively correlated with outbreak size (Fig. S4B). A decreasing pneumonic plague infectious period $(\gamma_{p}^{-1})$ was correlated with decreased outbreak size (Fig. S4A) and detectable duration (Fig. S4B). For the pneumonic SEIR model, transmission rate $(\beta_{p})$ was positively correlated with total mortality and duration (Fig. S4 C & D). Human birth rate $(b_{h})$ was positively correlated with total mortality. A decreasing pneumonic plague infectious period $(\gamma_{p}^{-1})$ was correlated with decreased outbreak size (Fig. S4C) and detectable duration (Fig. S4D). For the bubonic SIR model, flea searching efficiency $(\alpha)$, flea death rate $(d_{f})$, human recovery probability $(g_{h})$ and rat recovery probability $(g_{r})$ were all negatively correlated with total mortality (Fig S5A). Transmission rate from fleas to rats $(\beta_{r})$, flea death rate $(d_{f})$, human recovery rate $(\gamma_{b})$, and probability of rat recovery $(p_{r})$ were negatively correlated with outbreak duration (Fig S5B). Similarly, for the bubonic SEIR model, flea searching efficiency $(\alpha)$, flea death rate $(d_{f})$, and rat recovery probability $(g_{r})$ were all negatively correlated with total mortality (Fig S5C) and detectable outbreak duration (Fig S5D). For the bubonic SIR model incorporating rat dynamics, transmission rate from fleas to humans $(\beta_{b})$ correlated positively with total mortality, while flea searching efficiency $(\alpha)$, flea death rate $(d_{f})$, rat recovery probability $(g_{r})$, and rat carrying capacity $(K_{r}$) had negative effects on total mortality $(FigS6A)$. There were no significant parameters for detectable outbreak duration (Fig. S6B). For the bubonic SEIR model the same parameters had significant correlations with total mortality with the addition of probability of rat resistance $(p_{r})$ having a negative correlation (Fig. S6C). Probability of rat resistance $(p_{r})$ also had a negative correlation with detectable outbreak duration (Fig. S6D). Finally, for the combined bubonic/pneumonic SEIR model, flea transmission efficiency $(\alpha)$, longer infectious periods of pneumonic plague in humans $(\gamma_{p}^{-1})$, and a shorter pneumonic plague incubation period $(\sigma_{p}^{-1})$ was also correlated with a higher total mortality (Fig. S7A). Flea searching efficiency $(\alpha)$ was positively correlated with outbreak duration, and a decreased bubonic incubation period in humans $(\sigma_{b}^{-1})$ was correlated with longer detectable outbreaks (Fig. S7B).

## Pneumonic Plague SIR- Figure S4 (Panels A & B)

parameters <- c(beta_p = 0.45, gamma_p = 1/2.5, b_h=1/(25*365), d_h=1/(25*365))

#plot scatterplots
par(mfrow=c(1,2))
plot(pSIR$MaxInf~pSIR$beta_p, main= expression(paste("Effect of ", beta[p], " on Size")), xlab=expression(beta[p]), ylab= "Outbreak Size")
plot(pSIR$Thresh100~pSIR$beta_p, main= expression(paste("Effect of ", beta[p], " on Duration")), xlab=expression(beta[p]), ylab= "Observable Duration (days)")


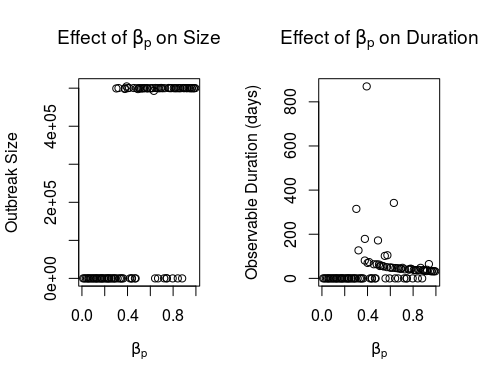


plot(pSIR$MaxInf~pSIR$gamma_p, main= expression(paste("Effect of ", gamma[p], " on Size")), xlab=expression(gamma[p]), ylab= "Outbreak Size")
plot(pSIR$Thresh100~pSIR$gamma_p, main= expression(paste("Effect of ", gamma[p], " on Duration")), xlab=expression(gamma[p]), ylab= "Observable Duration (days)")


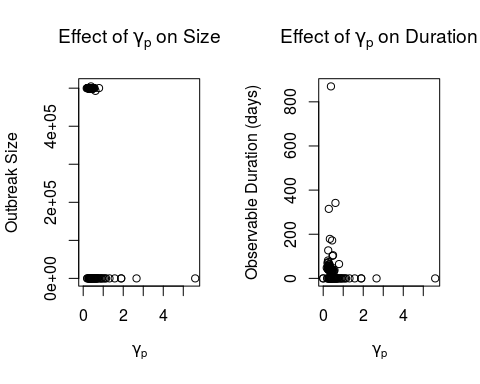


plot(pSIR$MaxInf~pSIR$b_h, main= expression(paste("Effect of ", b[h], " on Size")), xlab=expression(b[h]), ylab= "Outbreak Size")
plot(pSIR$Thresh100~pSIR$b_h, main= expression(paste("Effect of ", b[h], " on Duration")), xlab=expression(b[h]), ylab= "Observable Duration (days)")


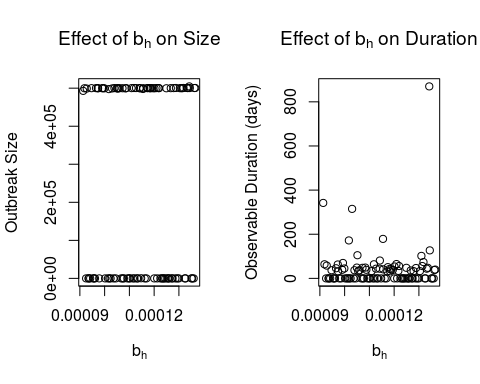


plot(pSIR$MaxInf~pSIR$d_h, main= expression(paste("Effect of ", d[h], " on Size")), xlab=expression(b[h]), ylab= "Outbreak Size")
plot(pSIR$Thresh100~pSIR$d_h, main= expression(paste("Effect of ", d[h], " on Duration")), xlab=expression(d[h]), ylab= "Observable Duration (days)")


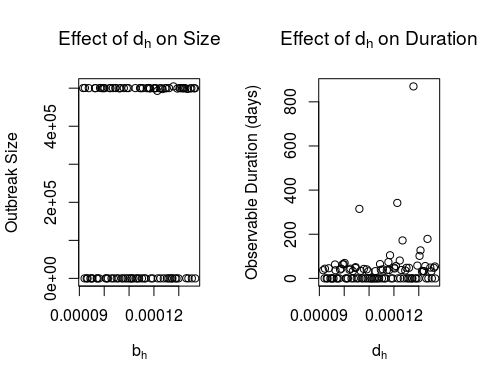


par(mfrow=c(1,2))
boxplot(pSIR$MaxInf, main= "Outbreak Size", ylab= "Number of Dead Humans", ylim=c(0,500000))
boxplot(pSIR$Thresh100, main= "Observable Outbreak Duration", ylab="Time (Days)")


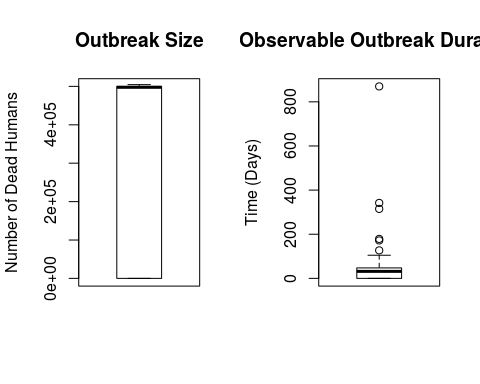


bonferroni.alpha <- 0.05/length(parameters)
prcc_size <- pcc(pSIR[,1:length(parameters)], pSIR$MaxInf, nboot = niter, rank=TRUE, conf=1-bonferroni.alpha)
prcc_duration <- pcc(pSIR[,1:length(parameters)], pSIR$Thresh100, nboot = niter, rank=TRUE, conf=1-bonferroni.alpha)

#plot correlation coefficients and confidence intervals for epidemic size and duration
size<-prcc_size$PRCC
size$param<-rownames(size)
colnames(size)[4:5] <- c("maxCI", "minCI")
size$maxCI[which(size$maxCI>1)]<-1
size$maxCI[which(size$maxCI< -1)]<- -1
size$minCI[which(size$minCI>1)]<-1
size$minCI[which(size$minCI< -1)]<- -1

duration<-prcc_duration$PRCC
duration$param<-rownames(duration)
colnames(duration)[4:5] <- c("maxCI", "minCI")
duration$maxCI[which(duration$maxCI>1)]<-1
duration$maxCI[which(duration$maxCI< -1)]<- -1
duration$minCI[which(duration$minCI>1)]<-1
duration$minCI[which(duration$minCI< -1)]<- -1


A<- ggplot(size, aes(x=param, y = original)) +
 geom_point(size = 4)+
 geom_errorbar(aes(ymax = maxCI, ymin = minCI))+
 ggtitle("A")+
 xlab("Parameters")+
 ylab ("Partial Rank Correlation Coefficients")+
 scale_x_discrete(labels = c("beta_p" = expression(beta[p]),
 "b_h" = expression(b[h]),"d_h" = expression(d[h]), "gamma_p" = expression(gamma[p])))+
 ylim(-1,1)


B<-ggplot(duration, aes(x=param, y = original)) +
 geom_point(size = 4)+
 geom_errorbar(aes(ymax = maxCI, ymin = minCI))+
 ggtitle("B")+
 xlab("Parameters")+
 ylab (" ")+
 scale_x_discrete(labels = c("beta_p" = expression(beta[p]),
 "b_h" = expression(b[h]),"d_h" = expression(d[h]), "gamma_p" = expression(gamma[p])))+
 ylim(-1,1)

## Pneumonic Plague SEIR- Figure S4 (Panels C & D)

parameters <- c(beta_p = 0.45, sigma_p= 1/4.3, gamma_p = 1/2.5, b_h=1/(25*365), d_h=1/(25*365))

par(mfrow=c(1,2))
plot(pSEIR$MaxInf~pSEIR$beta_p, main= expression(paste("Effect of ", beta[p], " on Size")), xlab=expression(beta[p]), ylab="Outbreak Size")
plot(pSEIR$Thresh100~pSEIR$beta_p, main= expression(paste("Effect of ", beta[p], " on Duration")), xlab=expression(beta[p]), ylab="Detectable Outbreak Duration (days)")


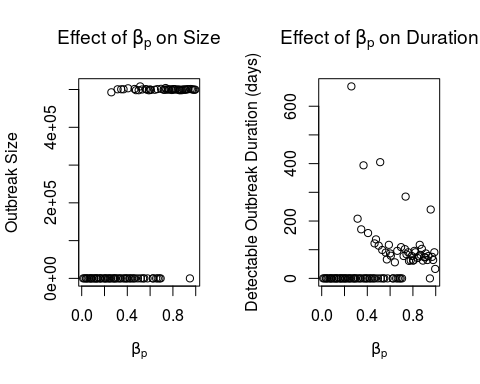


plot(pSEIR$MaxInf~pSEIR$sigma_p, main= expression(paste("Effect of ", sigma[p], " on Size")), xlab=expression(sigma[p]), ylab="Outbreak Size")
plot(pSEIR$Thresh100~pSEIR$sigma_p, main= expression(paste("Effect of ", sigma[p], " on Duration")), xlab=expression(sigma[p]), ylab="Detectable Outbreak Duration (days)")


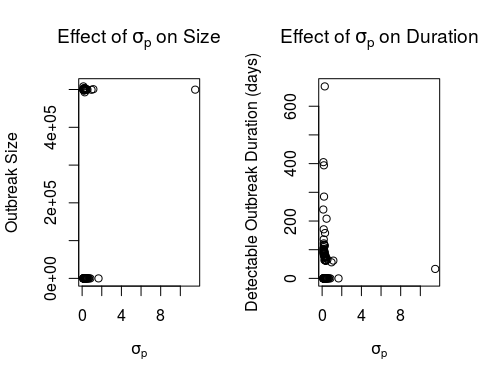


plot(pSEIR$MaxInf~pSEIR$gamma_p, main= expression(paste("Effect of ", gamma[p], " on Size")), xlab=expression(gamma[p]), ylab="Outbreak Size")
plot(pSEIR$Thresh100~pSEIR$gamma_p, main= expression(paste("Effect of ", gamma[p], " on Duration")), xlab=expression(gamma[p]), ylab="Detectable Outbreak Duration (days)")


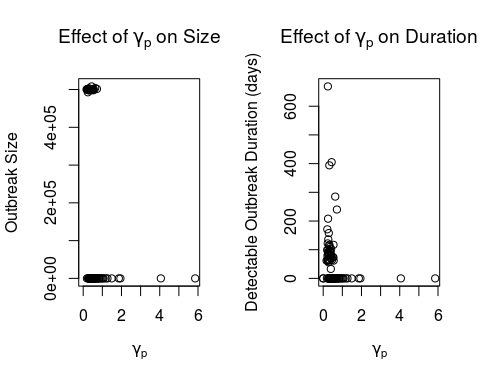


plot(pSEIR$MaxInf~pSEIR$b_h, main= expression(paste("Effect of ", b[h], " on Size")), xlab=expression(b[h]), ylab="Outbreak Size")
plot(pSEIR$Thresh100~pSEIR$b_h, main= expression(paste("Effect of ", b[h], " on Duration")), xlab=expression(b[h]), ylab="Detectable Outbreak Duration (days)")


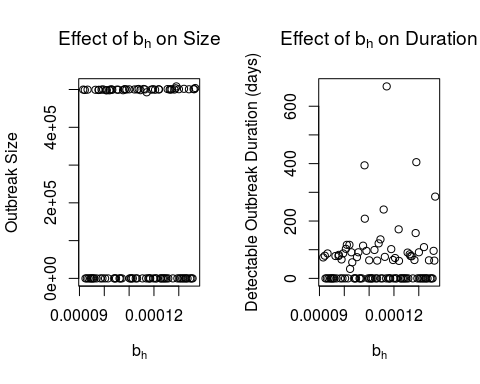


plot(pSEIR$MaxInf~pSEIR$d_h, main= expression(paste("Effect of ", d[h], " on Size")), xlab=expression(d[h]), ylab="Outbreak Size")
plot(pSEIR$Thresh100~pSEIR$d_h, main= expression(paste("Effect of ", d[h], " on Duration")), xlab=expression(d[h]), ylab="Detectable Outbreak Duration (days)")


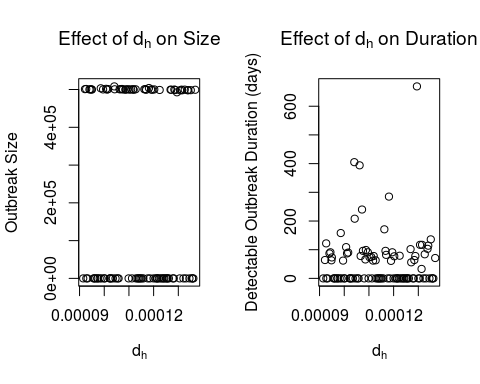


par(mfrow=c(1,2))
boxplot(pSEIR$MaxInf, main= "Outbreak Size", ylab= "Number of Dead Humans", ylim=c(0,500000))
boxplot(pSEIR$Thresh100, main= "Outbreak Duration", ylab="Time (Days)")


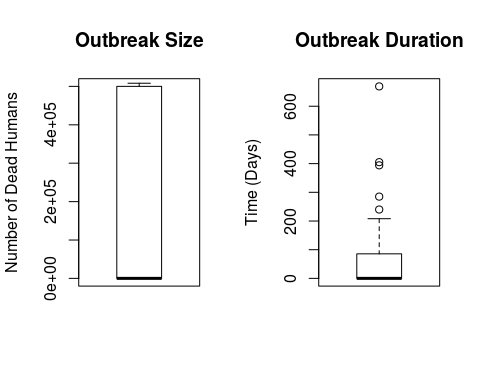


bonferroni.alpha <- 0.05/length(parameters)
prcc_size <- pcc(pSEIR[,1:length(parameters)], pSEIR$MaxInf, nboot = niter, rank=TRUE, conf=1-bonferroni.alpha)
prcc_duration <- pcc(pSEIR[,1:length(parameters)], pSEIR$Thresh100, nboot = niter, rank=TRUE, conf=1-bonferroni.alpha)

#plot correlation coefficients and confidence intervals for epidemic size and duration
size<-prcc_size$PRCC
size$param<-rownames(size)
colnames(size)[4:5] <- c("maxCI", "minCI")
size$maxCI[which(size$maxCI>1)]<-1
size$maxCI[which(size$maxCI< -1)]<- -1
size$minCI[which(size$minCI>1)]<-1
size$minCI[which(size$minCI< -1)]<- -1

duration<-prcc_duration$PRCC
duration$param<-rownames(duration)
colnames(duration)[4:5] <- c("maxCI", "minCI")
duration$maxCI[which(duration$maxCI>1)]<-1
duration$maxCI[which(duration$maxCI< -1)]<- -1
duration$minCI[which(duration$minCI>1)]<-1
duration$minCI[which(duration$minCI< -1)]<- -1

C<- ggplot(size, aes(x=param, y = original)) +
 geom_point(size = 4)+
 geom_errorbar(aes(ymax = maxCI, ymin = minCI))+
 ggtitle("C")+
 xlab("Parameters")+
 ylab ("Partial Rank Correlation Coefficients")+
 scale_x_discrete(labels = c("beta_p" = expression(beta[p]),
 "b_h" = expression(b[h]),"d_h" = expression(d[h]), "gamma_p" = expression(gamma[p]), "sigma_p"=expression(sigma[p])))+
 ylim(-1,1)

D<-ggplot(duration, aes(x=param, y = original)) +
 geom_point(size = 4)+
 geom_errorbar(aes(ymax = maxCI, ymin = minCI))+
 ggtitle("D")+
 xlab("Parameters")+
 ylab (" ")+
 scale_x_discrete(labels = c("beta_p" = expression(beta[p]),
 "b_h" = expression(b[h]),"d_h" = expression(d[h]), "gamma_p" = expression(gamma[p]), "sigma_p"=expression(sigma[p])))+
 ylim(-1,1)

multiplot(A, B, cols=2)


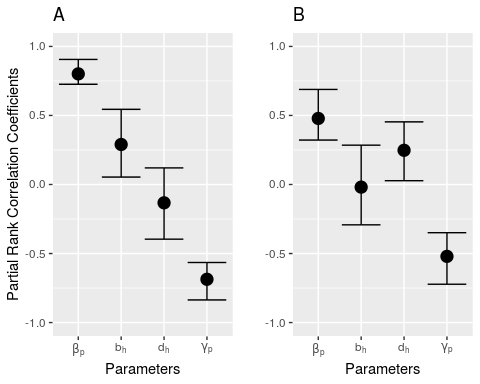


tiff("FigS4.tiff", height =22.23 , width =19.05, units = "cm", compression = "lzw", res = 600)
multiplot(A, B, C, D, cols=2)
dev.off()

## png
## 2

## Bubonic SIR model- Figure S5 (Panels A & B)

parameters <- c(beta_r = 0.09, alpha=3/500000, gamma_r = 1/5.15, g_r=0.1, r_f=0.0084, K_f=6, d_f=1/5, beta_h=0.19, gamma_h=1/10, g_h=0.34, b_h=1/(25*365), d_h=1/(25*365)) #you can play with transmission and recovery rates here

par(mfrow=c(1,2))
plot(bSIR$MaxInf~bSIR$beta_r, main= expression(paste("Effect of ", beta[r], " on Size")), xlab=expression(beta[r]), ylab="Outbreak Size")
plot(bSIR$Thresh100~bSIR$beta_r, main= expression(paste("Effect of ", beta[r], " on Duration")), xlab=expression(beta[r]), ylab="Detectable Duration (days)")


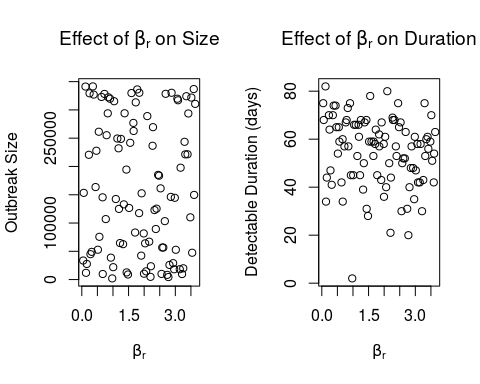


plot(bSIR$MaxInf~bSIR$alpha, main= expression(paste("Effect of ", alpha, " on Size")), xlab=expression(alpha), ylab="Outbreak Size")
plot(bSIR$Thresh100~bSIR$alpha, main= expression(paste("Effect of ", alpha, " on Duration")), xlab=expression(alhpa), ylab="Detectable Duration (days)")


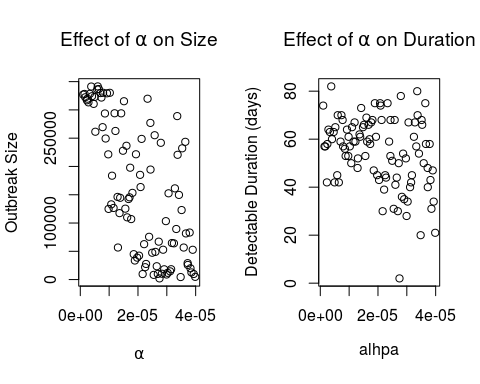


plot(bSIR$MaxInf~bSIR$gamma_r, main= expression(paste("Effect of ", gamma[r], " on Size")), xlab=expression(gamma[r]), ylab="Outbreak Size")
plot(bSIR$Thresh100~bSIR$gamma_r, main= expression(paste("Effect of ", gamma[r], " on Duration")), xlab=expression(gamma[r]), ylab="Detectable Duration (days)")


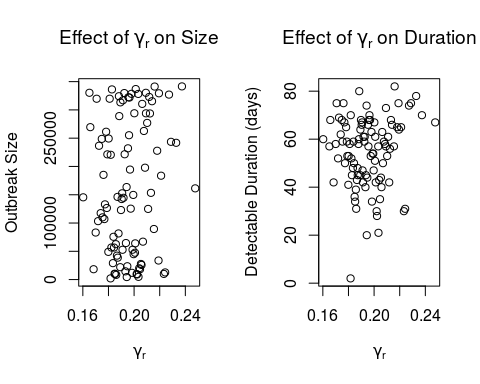


plot(bSIR$MaxInf~bSIR$g_r, main= expression(paste("Effect of ", g[r], " on Size")), xlab=expression(g[r]), ylab="Outbreak Size")
plot(bSIR$Thresh100~bSIR$g_r, main= expression(paste("Effect of ", g[r], " on Duration")), xlab=expression(g[r]), ylab="Detectable Duration (days)")


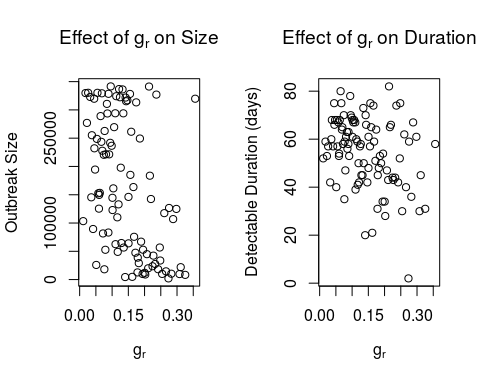


plot(bSIR$MaxInf~bSIR$r_f, main= expression(paste("Effect of ", r[f], " on Size")), xlab=expression(r[f]), ylab="Outbreak Size")
plot(bSIR$Thresh100~bSIR$r_f, main= expression(paste("Effect of ", r[f], " on Duration")), xlab=expression(r[f]), ylab="Detectable Duration (days)")


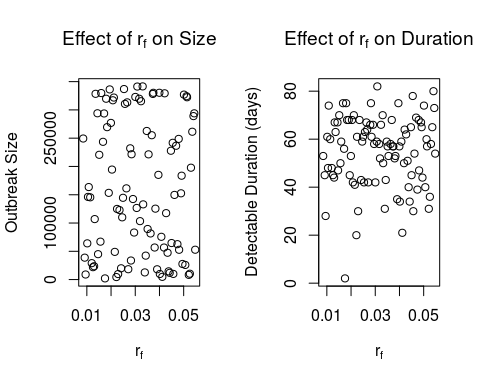


plot(bSIR$MaxInf~bSIR$K_f, main= expression(paste("Effect of ", K[f], " on Size")), xlab=expression(K[f]), ylab="Outbreak Size")
plot(bSIR$Thresh100~bSIR$K_f, main= expression(paste("Effect of ", K[f], " on Duration")), xlab=expression(K[f]), ylab="Detectable Duration (days)")


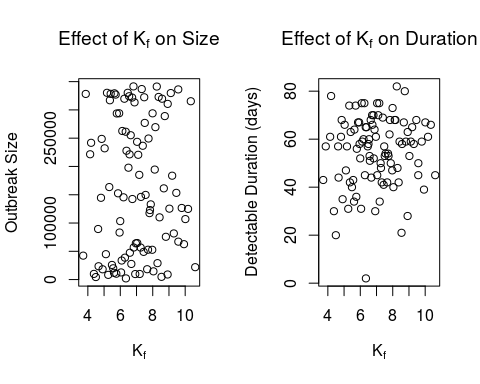


plot(bSIR$MaxInf~bSIR$d_f, main= expression(paste("Effect of ", d[f], " on Size")), xlab=expression(d[f]), ylab="Outbreak Size")
plot(bSIR$Thresh100~bSIR$d_f, main= expression(paste("Effect of ", d[f], " on Duration")), xlab=expression(d[f]), ylab="Detectable Duration (days)")


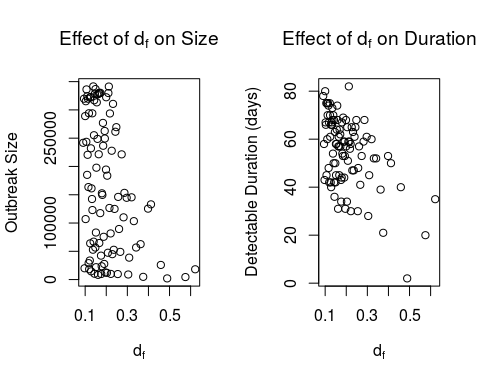


plot(bSIR$MaxInf~bSIR$beta_h, main= expression(paste("Effect of ", beta[b], " on Size")), xlab=expression(beta[b]), ylab="Outbreak Size")
plot(bSIR$Thresh100~bSIR$beta_h, main= expression(paste("Effect of ", beta[b], " on Duration")), xlab=expression(beta[b]), ylab="Detectable Duration (days)")


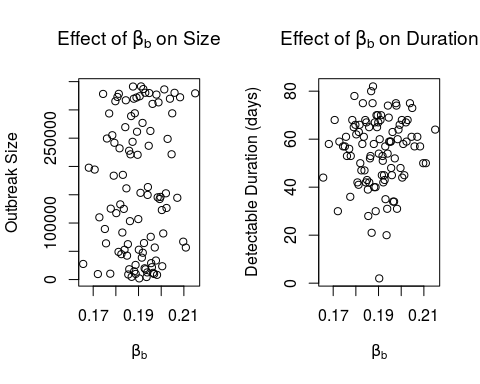


plot(bSIR$MaxInf~bSIR$gamma_h, main= expression(paste("Effect of ", gamma[b], " on Size")), xlab=expression(gamma[b]), ylab="Outbreak Size")
plot(bSIR$Thresh100~bSIR$gamma_h, main= expression(paste("Effect of ", gamma[b], " on Duration")), xlab=expression(gamma[b]), ylab="Detectable Duration (days)")


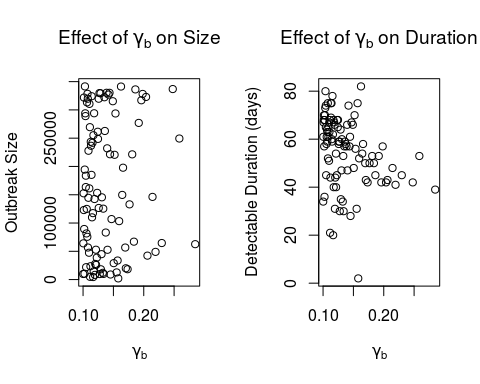


plot(bSIR$MaxInf~bSIR$g_h, main= expression(paste("Effect of ", g[h], " on Size")), xlab=expression(g[h]), ylab="Outbreak Size")
plot(bSIR$Thresh100~bSIR$g_h, main= expression(paste("Effect of ", g[h], " on Duration")), xlab=expression(g[h]), ylab="Detectable Duration (days)")


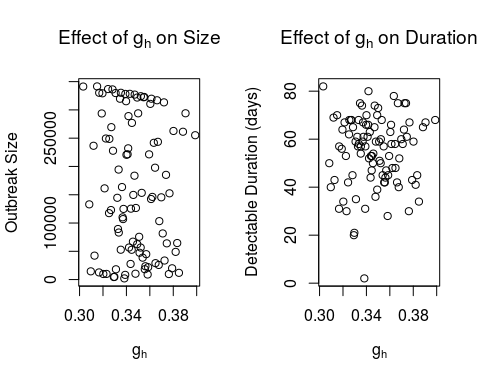


plot(bSIR$MaxInf~bSIR$b_h, main= expression(paste("Effect of ", b[h], " on Size")), xlab=expression(b[h]), ylab="Outbreak Size")
plot(bSIR$Thresh100~bSIR$b_h, main= expression(paste("Effect of ", b[h], " on Duration")), xlab=expression(b[h]), ylab="Detectable Duration (days)")


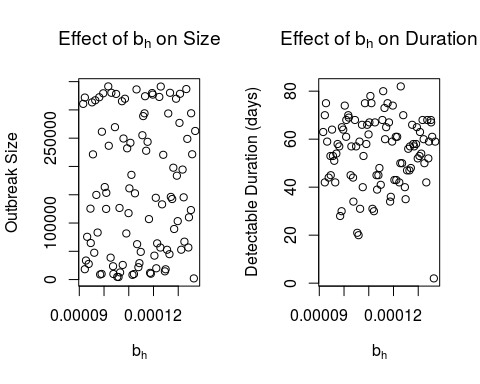


plot(bSIR$MaxInf~bSIR$d_h, main= expression(paste("Effect of ", d[h], " on Size")), xlab=expression(d[h]), ylab="Outbreak Size")
plot(bSIR$Thresh100~bSIR$d_h, main= expression(paste("Effect of ", d[h], " on Duration")), xlab=expression(d[h]), ylab="Detectable Duration (days)")


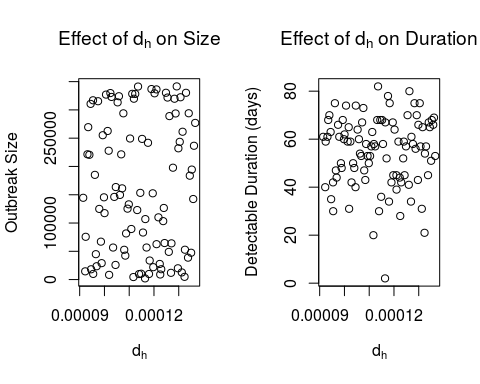


par(mfrow=c(1,2))
boxplot(bSIR$MaxInf, main= "Outbreak Size", ylab= "Number of Dead Humans", ylim=c(0,500000))
boxplot(bSIR$Thresh100, main= "Outbreak Duration", ylab="Time (Days)")


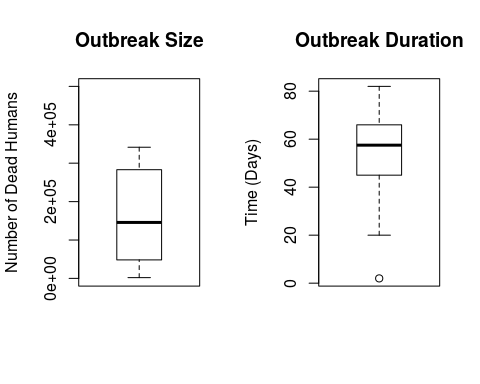


bonferroni.alpha <- 0.05/length(parameters)
prcc_size <- pcc(bSIR[,1:length(parameters)], bSIR$MaxInf, nboot = niter, rank=TRUE, conf=1-bonferroni.alpha)
prcc_duration <- pcc(bSIR[,1:length(parameters)], bSIR$Thresh100, nboot = niter, rank=TRUE, conf=1-bonferroni.alpha)

#plot correlation coefficients and confidence intervals for epidemic size and duration
size<-prcc_size$PRCC
size$param<-rownames(size)
colnames(size)[4:5] <- c("maxCI", "minCI")
size$maxCI[which(size$maxCI>1)]<-1
size$maxCI[which(size$maxCI< -1)]<- -1
size$minCI[which(size$minCI>1)]<-1
size$minCI[which(size$minCI< -1)]<- -1

duration<-prcc_duration$PRCC
duration$param<-rownames(duration)
colnames(duration)[4:5] <- c("maxCI", "minCI")
duration$maxCI[which(duration$maxCI>1)]<-1
duration$maxCI[which(duration$maxCI< -1)]<- -1
duration$minCI[which(duration$minCI>1)]<-1
duration$minCI[which(duration$minCI< -1)]<- -1


A<- ggplot(size, aes(x=param, y = original)) +
 geom_point(size = 4)+
 geom_errorbar(aes(ymax = maxCI, ymin = minCI))+
 ggtitle("A")+
 xlab("Parameters")+
 ylab ("Partial Rank Correlation Coefficients")+
 scale_x_discrete(labels = c("alpha"=expression(alpha),"beta_h" = expression(beta[b]), "beta_r"=expression(beta[r]),
 "b_h" = expression(b[h]),"d_h" = expression(d[h]), "d_f"=expression(d[f]), "gamma_h" = expression(gamma[b]), "gamma_r" = expression(gamma[r]), "g_h"=expression(g[h]), "g_r"=expression(g[r]), "K_f"=expression(K[f]), "r_f"=expression(r[f])))+
 ylim(-1,1)

B<-ggplot(duration, aes(x=param, y = original)) +
 geom_point(size = 4)+
 geom_errorbar(aes(ymax = maxCI, ymin = minCI))+
 ggtitle("B")+
 xlab("Parameters")+
 ylab (" ")+
 scale_x_discrete(labels = c("alpha"=expression(alpha),"beta_h" = expression(beta[b]), "beta_r"=expression(beta[r]),
 "b_h" = expression(b[h]),"d_h" = expression(d[h]), "d_f"=expression(d[f]), "gamma_h" = expression(gamma[b]), "gamma_r" = expression(gamma[r]), "g_h"=expression(g[h]), "g_r"=expression(g[r]), "K_f"=expression(K[f]), "r_f"=expression(r[f])))+
 ylim(-1,1)

## Bubonic SEIR- Figure S5 (Panels C & D)

parameters <- c(beta_r = 0.09, alpha=3/500000, gamma_r = 1/5.15, g_r=0.1, r_f=0.0084, K_f=6, d_f=1/5, beta_h=0.19, sigma_h= 1/4, gamma_h=1/10, g_h=0.34, b_h=1/(25*365), d_h=1/(25*365)) #you can play with transmission and recovery rates here

par(mfrow=c(1,2))
plot(bSEIR$MaxInf~bSEIR$beta_r, main= expression(paste("Effect of ", beta[r], " on Size")), xlab=expression(beta[r]), ylab="Outbreak Size")
plot(bSEIR$Thresh100~bSEIR$beta_r, main= expression(paste("Effect of ", beta[r], " on Duration")), xlab=expression(beta[r]), ylab="Detectable Duration (days)")


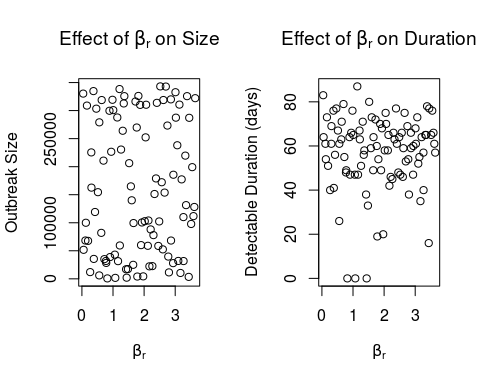


plot(bSEIR$MaxInf~bSEIR$alpha, main= expression(paste("Effect of ", alpha, " on Size")), xlab=expression(alpha), ylab="Outbreak Size")
plot(bSEIR$Thresh100~bSEIR$alpha, main= expression(paste("Effect of ", alpha, " on Duration")), xlab=expression(alpha), ylab="Detectable Duration (days)")


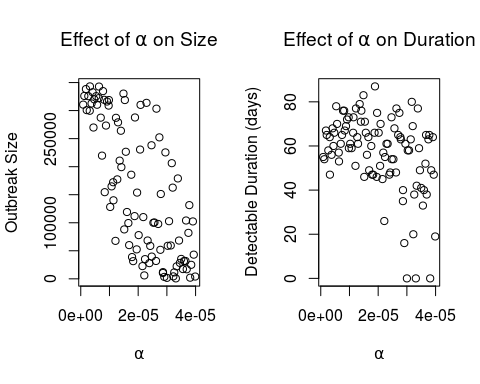


plot(bSEIR$MaxInf~bSEIR$gamma_r, main= expression(paste("Effect of ", gamma[r], " on Size")), xlab=expression(gamma[r]), ylab="Outbreak Size")
plot(bSEIR$Thresh100~bSEIR$gamma_r, main= expression(paste("Effect of ", gamma[r], " on Duration")), xlab=expression(gamma[r]), ylab="Detectable Duration (days)")


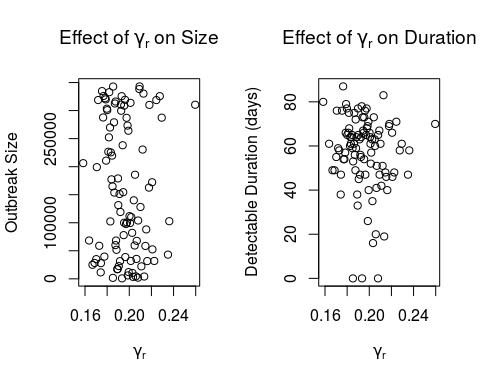


plot(bSEIR$MaxInf~bSEIR$g_r, main= expression(paste("Effect of ", g[r], " on Size")), xlab=expression(g[r]), ylab="Outbreak Size")
plot(bSEIR$Thresh100~bSEIR$g_r, main= expression(paste("Effect of ", g[r], " on Duration")), xlab=expression(g[r]), ylab="Detectable Duration (days)")


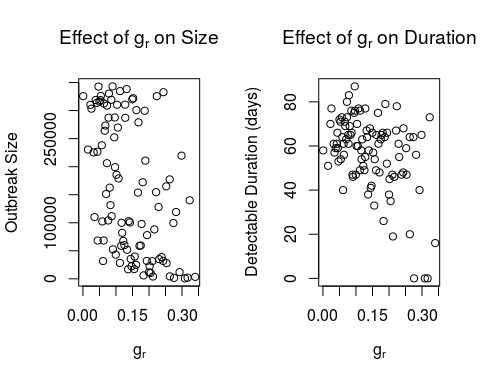


plot(bSEIR$MaxInf~bSEIR$r_f, main= expression(paste("Effect of ", r[f], " on Size")), xlab=expression(r[f]), ylab="Outbreak Size")
plot(bSEIR$Thresh100~bSEIR$r_f, main= expression(paste("Effect of ", r[f], " on Duration")), xlab=expression(r[f]), ylab="Detectable Duration (days)")


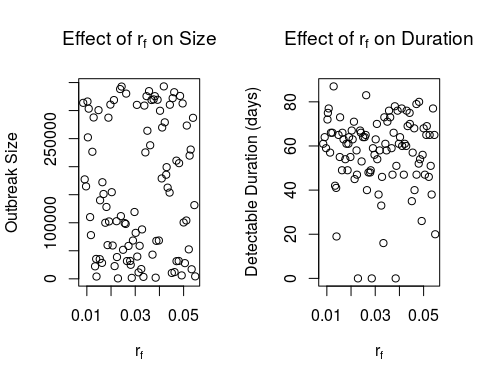


plot(bSEIR$MaxInf~bSEIR$K_f, main= expression(paste("Effect of ", K[f], " on Size")), xlab=expression(K[f]), ylab="Outbreak Size")
plot(bSEIR$Thresh100~bSEIR$K_f, main= expression(paste("Effect of ", K[f], " on Duration")), xlab=expression(K[f]), ylab="Detectable Duration (days)")


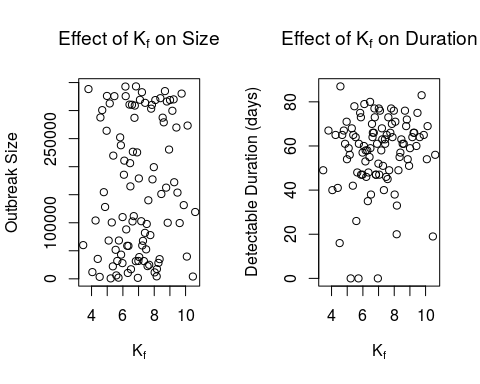


plot(bSEIR$MaxInf~bSEIR$d_f, main= expression(paste("Effect of ", d[f], " on Size")), xlab=expression(d[f]), ylab="Outbreak Size")
plot(bSEIR$Thresh100~bSEIR$d_f, main= expression(paste("Effect of ", d[f], " on Duration")), xlab=expression(d[f]), ylab="Detectable Duration (days)")


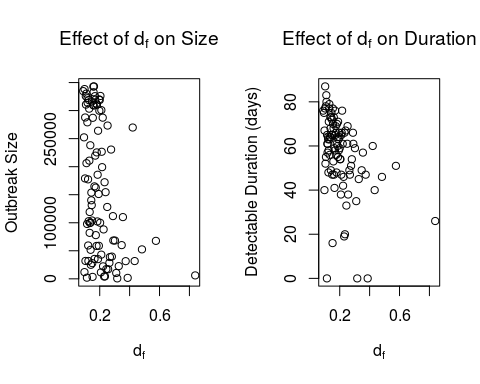


plot(bSEIR$MaxInf~bSEIR$beta_h, main= expression(paste("Effect of ", beta[b], " on Size")), xlab=expression(beta[b]), ylab="Outbreak Size")
plot(bSEIR$Thresh100~bSEIR$beta_h, main= expression(paste("Effect of ", beta[b], " on Duration")), xlab=expression(beta[b]), ylab="Detectable Duration (days)")


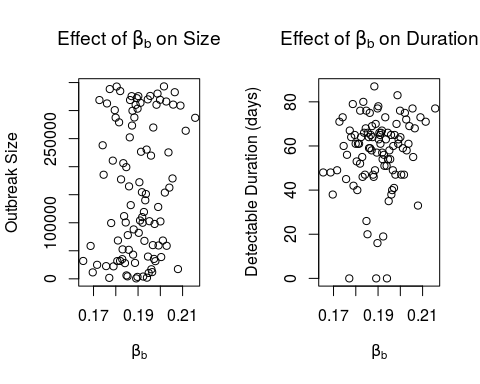


plot(bSEIR$MaxInf~bSEIR$gamma_h, main= expression(paste("Effect of ", gamma[b], " on Size")), xlab=expression(gamma[b]), ylab="Outbreak Size")
plot(bSEIR$Thresh100~bSEIR$gamma_h, main= expression(paste("Effect of ", gamma[b], " on Duration")), xlab=expression(gamma[b]), ylab="Detectable Duration (days)")


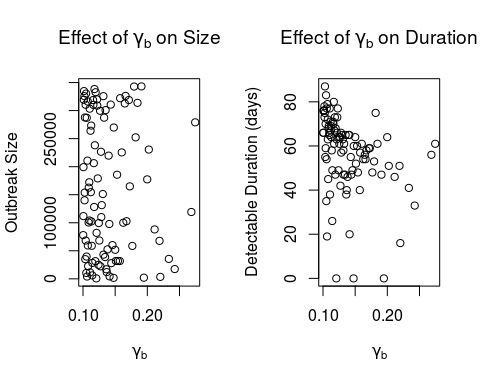


plot(bSEIR$MaxInf~bSEIR$g_h, main= expression(paste("Effect of ", g[h], " on Size")), xlab=expression(g[h]), ylab="Outbreak Size")
plot(bSEIR$Thresh100~bSEIR$g_h, main= expression(paste("Effect of ", g[h], " on Duration")), xlab=expression(g[h]), ylab="Detectable Duration (days)")


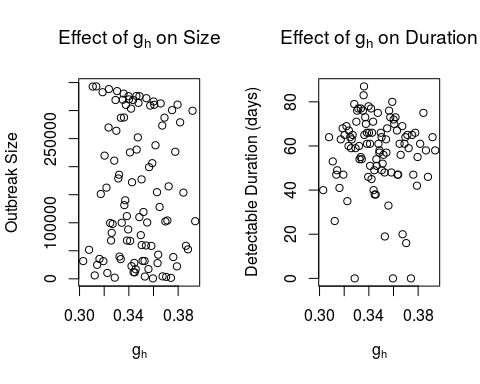


plot(bSEIR$MaxInf~bSEIR$b_h, main= expression(paste("Effect of ", b[h], " on Size")), xlab=expression(b[h]), ylab="Outbreak Size")
plot(bSEIR$Thresh100~bSEIR$b_h, main= expression(paste("Effect of ", b[h], " on Duration")), xlab=expression(b[h]), ylab="Detectable Duration (days)")


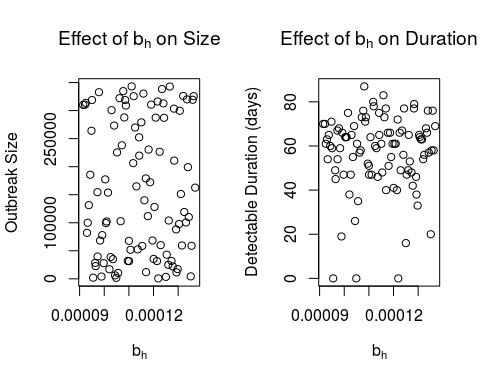


plot(bSEIR$MaxInf~bSEIR$d_h, main= expression(paste("Effect of ", d[h], " on Size")), xlab=expression(d[h]), ylab="Outbreak Size")
plot(bSEIR$Thresh100~bSEIR$d_h, main= expression(paste("Effect of ", d[h], " on Duration")), xlab=expression(d[h]), ylab="Detectable Duration (days)")


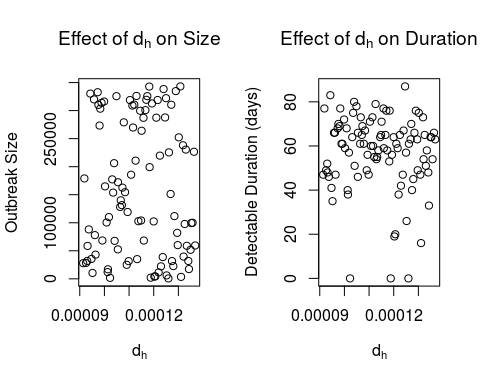


plot(bSEIR$MaxInf~bSEIR$sigma_h, main= expression(paste("Effect of ", sigma[b], " on Size")), xlab=expression(sigma_h[h]), ylab="Outbreak Size")
plot(bSEIR$Thresh100~bSEIR$sigma_h, main= expression(paste("Effect of ", sigma[b], " on Duration")), xlab=expression(sigma[b]), ylab="Detectable Duration (days)")


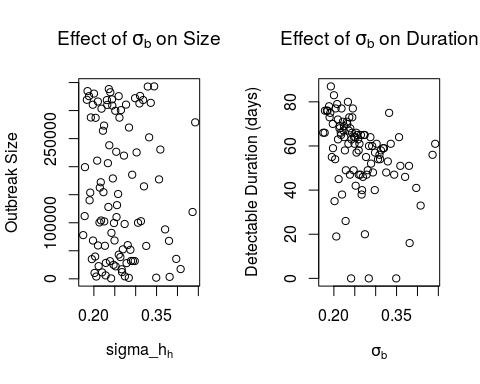


par(mfrow=c(1,2))
boxplot(bSEIR$MaxInf, main= "Outbreak Size", ylab= "Number of Dead Humans", ylim=c(0,500000))
boxplot(bSEIR$Thresh100, main= "Outbreak Duration", ylab="Time (Days)")


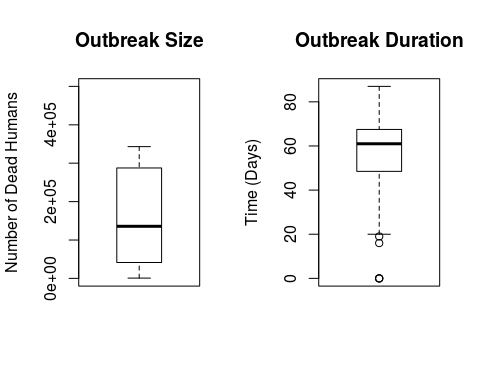


bonferroni.alpha <- 0.05/length(parameters)
prcc_size <- pcc(bSEIR[,1:length(parameters)], bSEIR$MaxInf, nboot = niter, rank=TRUE, conf=1-bonferroni.alpha)
prcc_duration <- pcc(bSEIR[,1:length(parameters)], bSEIR$Thresh100, nboot = niter, rank=TRUE, conf=1-bonferroni.alpha)

#plot correlation coefficients and confidence intervals for epidemic size and duration
size<-prcc_size$PRCC
size$param<-rownames(size)
colnames(size)[4:5] <- c("maxCI", "minCI")
size$maxCI[which(size$maxCI>1)]<-1
size$maxCI[which(size$maxCI< -1)]<- -1
size$minCI[which(size$minCI>1)]<-1
size$minCI[which(size$minCI< -1)]<- -1

duration<-prcc_duration$PRCC
duration$param<-rownames(duration)
colnames(duration)[4:5] <- c("maxCI", "minCI")
duration$maxCI[which(duration$maxCI>1)]<-1
duration$maxCI[which(duration$maxCI< -1)]<- -1
duration$minCI[which(duration$minCI>1)]<-1
duration$minCI[which(duration$minCI< -1)]<- -1


C<- ggplot(size, aes(x=param, y = original)) +
 geom_point(size = 4)+
 geom_errorbar(aes(ymax = maxCI, ymin = minCI))+
 ggtitle("C")+
 xlab("Parameters")+
 ylab ("Partial Rank Correlation Coefficients")+
 scale_x_discrete(labels = c("alpha"=expression(alpha),"beta_h" = expression(beta[b]), "beta_r"=expression(beta[r]),
 "b_h" = expression(b[h]),"d_h" = expression(d[h]), "d_f"=expression(d[f]), "sigma_h"=expression(sigma[b]), "gamma_h" = expression(gamma[b]), "gamma_r" = expression(gamma[r]), "g_h"=expression(g[h]), "g_r"=expression(g[r]), "K_f"=expression(K[f]), "r_f"=expression(r[f])))+
 ylim(-1,1)

D<-ggplot(duration, aes(x=param, y = original)) +
 geom_point(size = 4)+
 geom_errorbar(aes(ymax = maxCI, ymin = minCI))+
 ggtitle("D")+
 xlab("Parameters")+
 ylab (" ")+
 scale_x_discrete(labels = c("alpha"=expression(alpha),"beta_h" = expression(beta[b]), "beta_r"=expression(beta[r]),
 "b_h" = expression(b[h]),"d_h" = expression(d[h]), "d_f"=expression(d[f]), "sigma_h"=expression(sigma[b]), "gamma_h" = expression(gamma[b]), "gamma_r" = expression(gamma[r]), "g_h"=expression(g[h]), "g_r"=expression(g[r]), "K_f"=expression(K[f]), "r_f"=expression(r[f])))+
 ylim(-1,1)


tiff("FigS5.tiff", height =22.23 , width =19.05, units = "cm", compression = "lzw", res = 1200)
multiplot(A, B, C, D, cols=2)
dev.off()

## png
## 2

## Bubonic SIR with rat carrying capacity and resistance- Figure S6 (Panels A & B)

parameters <- c(r_r=0.014, K_r=499999, p_r=0.975, d_r=0.00055, beta_r = 0.09, alpha=3/500000, gamma_r = 1/5.15, g_r=0.1, r_f=0.0084, K_f=6, d_f=1/5, beta_h=0.19, gamma_h=1/10, g_h=0.34, b_h=1/(25*365), d_h=1/(25*365)) #you can play with transmission and recovery rates here

par(mfrow=c(1,2))
plot(bSIRrK$MaxInf~bSIRrK$r_r, main= expression(paste("Effect of ", r[r], " on Size")), xlab=expression(r[r]), ylab="Outbreak Size")
plot(bSIRrK$Thresh100~bSIRrK$r_r, main= expression(paste("Effect of ", r[r], " on Duration")), xlab=expression(r[r]), ylab="Detectable Duration (days)")


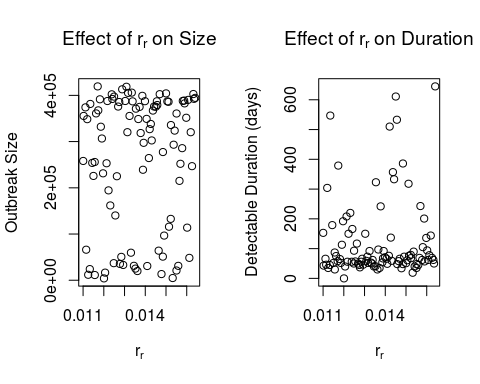


plot(bSIRrK$MaxInf~bSIRrK$K_r, main= expression(paste("Effect of ", K[r], " on Size")), xlab=expression(K[r]), ylab="Outbreak Size")
plot(bSIRrK$Thresh100~bSIRrK$K_r, main= expression(paste("Effect of ", K[r], " on Duration")), xlab=expression(K[r]), ylab="Detectable Duration (days)")


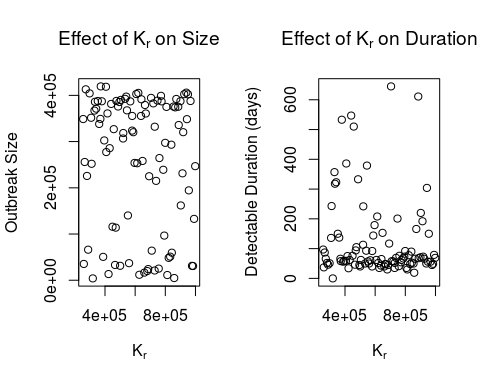


plot(bSIRrK$MaxInf~bSIRrK$p_r, main= expression(paste("Effect of ", p[r], " on Size")), xlab=expression(p[r]), ylab="Outbreak Size")
plot(bSIRrK$Thresh100~bSIRrK$p_r, main= expression(paste("Effect of ", p[r], " on Duration")), xlab=expression(p[r]), ylab="Detectable Duration (days)")


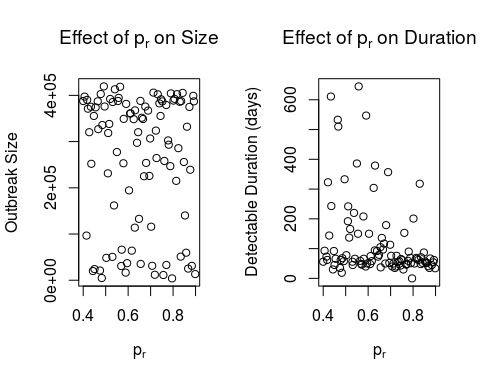


plot(bSIRrK$MaxInf~bSIRrK$d_r, main= expression(paste("Effect of ", d[r], " on Size")), xlab=expression(d[r]), ylab="Outbreak Size")
plot(bSIRrK$Thresh100~bSIRrK$d_r, main= expression(paste("Effect of ", d[r], " on Duration")), xlab=expression(d[r]), ylab="Detectable Duration (days)")


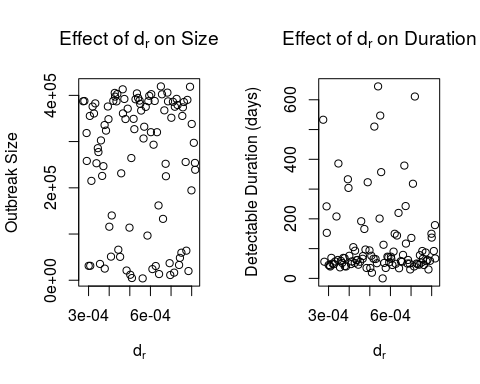


plot(bSIRrK$MaxInf~bSIRrK$beta_r, main= expression(paste("Effect of ", beta[r], " on Size")), xlab=expression(beta[r]), ylab="Outbreak Size")
plot(bSIRrK$Thresh100~bSIRrK$beta_r, main= expression(paste("Effect of ", beta[r], " on Duration")), xlab=expression(beta[r]), ylab="Detectable Duration (days)")


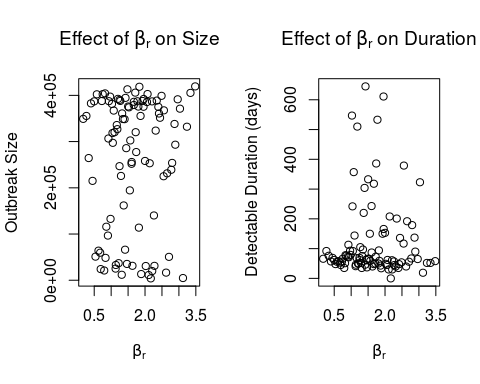


plot(bSIRrK$MaxInf~bSIRrK$alpha, main= expression(paste("Effect of ", alpha, " on Size")), xlab=expression(alpha), ylab="Outbreak Size")
plot(bSIRrK$Thresh100~bSIRrK$alpha, main= expression(paste("Effect of ", alpha, " on Duration")), xlab=expression(alpha), ylab="Detectable Duration (days)")


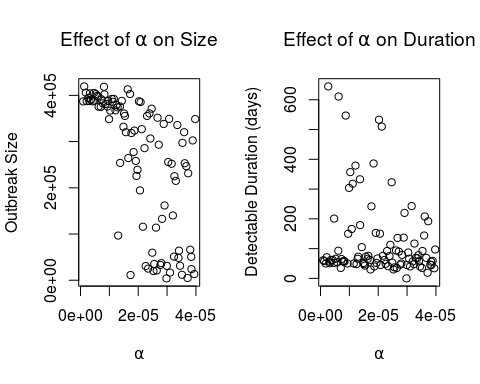


plot(bSIRrK$MaxInf~bSIRrK$gamma_r, main= expression(paste("Effect of ", gamma[r], " on Size")), xlab=expression(gamma[r]), ylab="Outbreak Size")
plot(bSIRrK$Thresh100~bSIRrK$gamma_r, main= expression(paste("Effect of ", gamma[r], " on Duration")), xlab=expression(gamma[r]), ylab="Detectable Duration (days)")


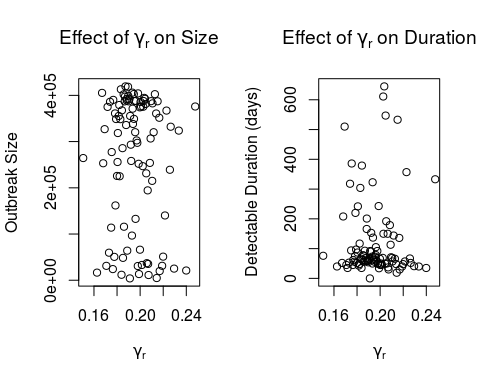


plot(bSIRrK$MaxInf~bSIRrK$g_r, main= expression(paste("Effect of ", g[r], " on Size")), xlab=expression(g[r]), ylab="Outbreak Size")
plot(bSIRrK$Thresh100~bSIRrK$g_r, main= expression(paste("Effect of ", g[r], " on Duration")), xlab=expression(g[r]), ylab="Detectable Duration (days)")


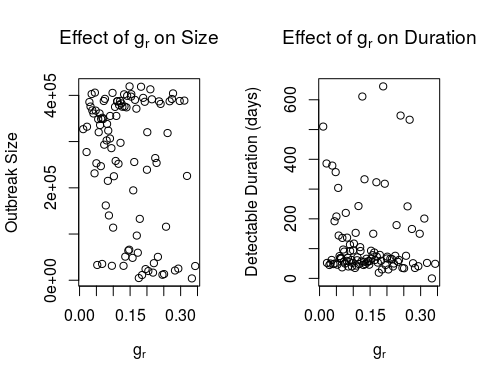


plot(bSIRrK$MaxInf~bSIRrK$r_f, main= expression(paste("Effect of ", r[f], " on Size")), xlab=expression(r[f]), ylab="Outbreak Size")
plot(bSIRrK$Thresh100~bSIRrK$r_f, main= expression(paste("Effect of ", r[f], " on Duration")), xlab=expression(r[f]), ylab="Detectable Duration (days)")


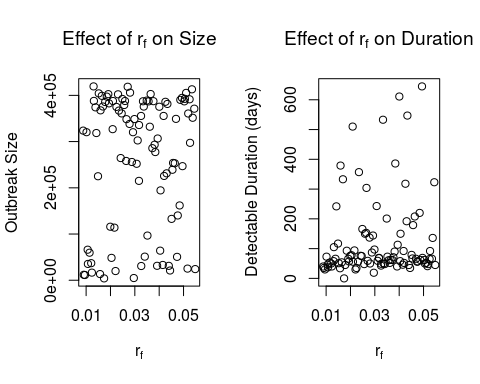


plot(bSIRrK$MaxInf~bSIRrK$K_f, main= expression(paste("Effect of ", K[f], " on Size")), xlab=expression(K[f]), ylab="Outbreak Size")
plot(bSIRrK$Thresh100~bSIRrK$K_f, main= expression(paste("Effect of ", K[f], " on Duration")), xlab=expression(K[f]), ylab="Detectable Duration (days)")


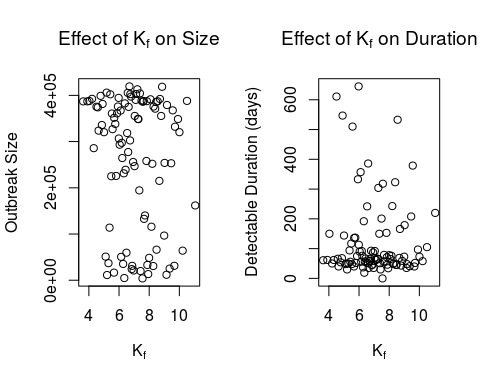


plot(bSIRrK$MaxInf~bSIRrK$d_f, main= expression(paste("Effect of ", d[f], " on Size")), xlab=expression(d[f]), ylab="Outbreak Size")
plot(bSIRrK$Thresh100~bSIRrK$d_f, main= expression(paste("Effect of ", d[f], " on Duration")), xlab=expression(d[f]), ylab="Detectable Duration (days)")


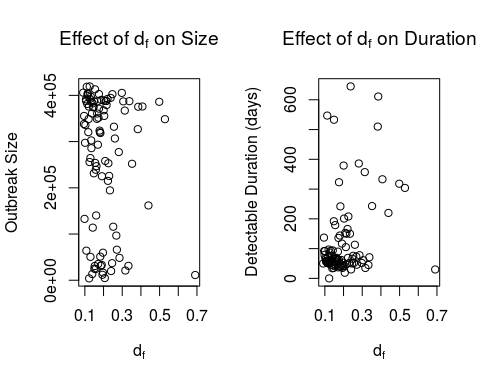


plot(bSIRrK$MaxInf~bSIRrK$beta_h, main= expression(paste("Effect of ", beta[b], " on Size")), xlab=expression(beta[b]), ylab="Outbreak Size")
plot(bSIRrK$Thresh100~bSIRrK$beta_h, main= expression(paste("Effect of ", beta[b], " on Duration")), xlab=expression(beta[b]), ylab="Detectable Duration (days)")


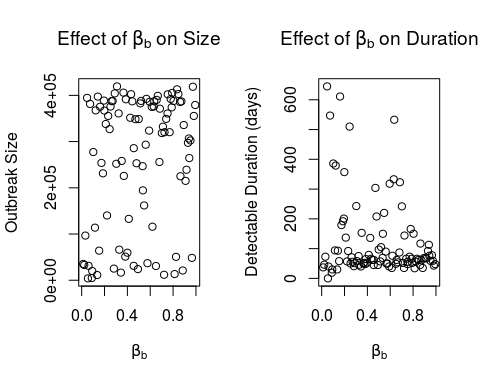


plot(bSIRrK$MaxInf~bSIRrK$gamma_h, main= expression(paste("Effect of ", gamma[b], " on Size")), xlab=expression(gamma[b]), ylab="Outbreak Size")
plot(bSIRrK$Thresh100~bSIRrK$gamma_h, main= expression(paste("Effect of ", gamma[b], " on Duration")), xlab=expression(gamma[b]), ylab="Detectable Duration (days)")


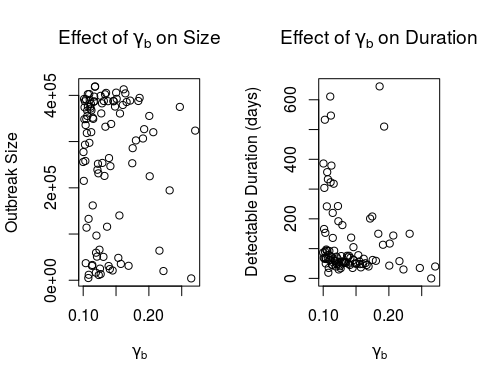


plot(bSIRrK$MaxInf~bSIRrK$g_h, main= expression(paste("Effect of ", g[h], " on Size")), xlab=expression(g[h]), ylab="Outbreak Size")
plot(bSIRrK$Thresh100~bSIRrK$g_h, main= expression(paste("Effect of ", g[h], " on Duration")), xlab=expression(g[h]), ylab="Detectable Duration (days)")


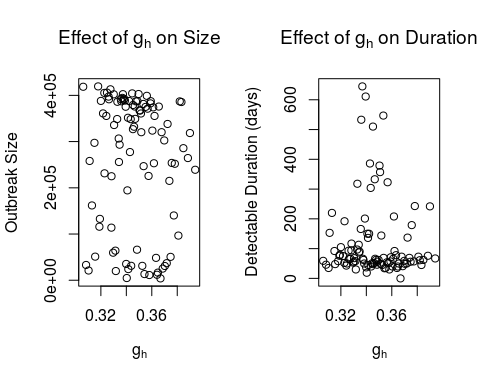


plot(bSIRrK$MaxInf~bSIRrK$b_h, main= expression(paste("Effect of ", b[h], " on Size")), xlab=expression(b[h]), ylab="Outbreak Size")
plot(bSIRrK$Thresh100~bSIRrK$b_h, main= expression(paste("Effect of ", b[h], " on Duration")), xlab=expression(r[r]), ylab="Detectable Duration (days)")


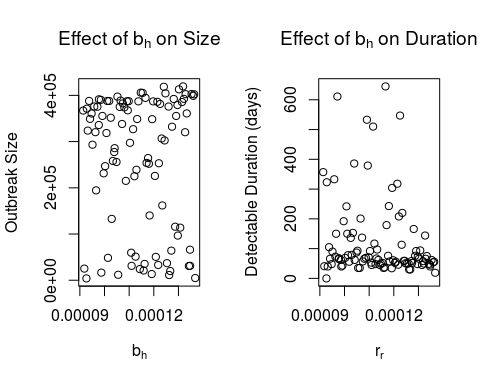


plot(bSIRrK$MaxInf~bSIRrK$d_h, main= expression(paste("Effect of ", d[h], " on Size")), xlab=expression(d[h]), ylab="Outbreak Size")
plot(bSIRrK$Thresh100~bSIRrK$d_h, main= expression(paste("Effect of ", d[h], " on Duration")))


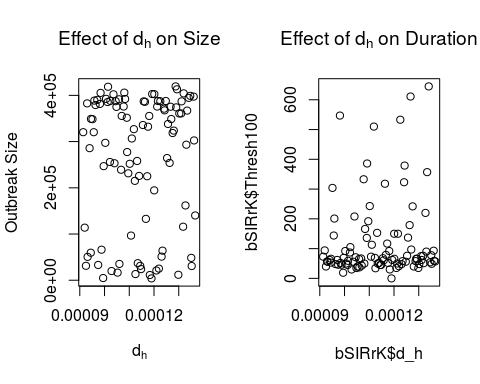


par(mfrow=c(1,2))
boxplot(bSIRrK$MaxInf, main= "Outbreak Size", ylab= "Number of Dead Humans", ylim=c(0,500000))
boxplot(bSIRrK$Thresh100, main= "Outbreak Duration", ylab="Time (Days)")


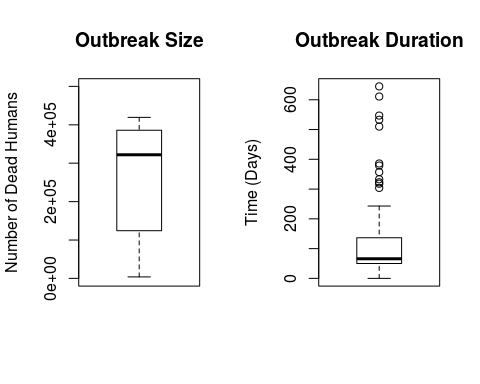


bonferroni.alpha <- 0.05/length(parameters)
prcc_size <- pcc(bSIRrK[,1:length(parameters)], bSIRrK$MaxInf, nboot = niter, rank=TRUE, conf=1-bonferroni.alpha)
prcc_duration <- pcc(bSIRrK[,1:length(parameters)], bSIRrK$Thresh100, nboot = niter, rank=TRUE, conf=1-bonferroni.alpha)

#plot correlation coefficients and confidence intervals for epidemic size and duration
#plot correlation coefficients and confidence intervals for epidemic size and duration
size<-prcc_size$PRCC
size$param<-rownames(size)
colnames(size)[4:5] <- c("maxCI", "minCI")
size$maxCI[which(size$maxCI>1)]<-1
size$maxCI[which(size$maxCI< -1)]<- -1
size$minCI[which(size$minCI>1)]<-1
size$minCI[which(size$minCI< -1)]<- -1

duration<-prcc_duration$PRCC
duration$param<-rownames(duration)
colnames(duration)[4:5] <- c("maxCI", "minCI")
duration$maxCI[which(duration$maxCI>1)]<-1
duration$maxCI[which(duration$maxCI< -1)]<- -1
duration$minCI[which(duration$minCI>1)]<-1
duration$minCI[which(duration$minCI< -1)]<- -1


A<- ggplot(size, aes(x=param, y = original)) +
 geom_point(size = 4)+
 geom_errorbar(aes(ymax = maxCI, ymin = minCI))+
 ggtitle("A")+
 xlab("Parameters")+
 ylab ("Partial Rank Correlation Coefficients")+
 scale_x_discrete(labels = c("r_r"=expression(r[r]), "d_r"=expression(d[r]), "K_r"=expression(K[r]), "p_r"=expression(p[r]), "alpha"=expression(alpha),"beta_h" = expression(beta[b]), "beta_r"=expression(beta[r]),
 "b_h" = expression(b[h]),"d_h" = expression(d[h]), "d_f"=expression(d[f]), "gamma_h" = expression(gamma[b]), "gamma_r" = expression(gamma[r]), "g_h"=expression(g[h]), "g_r"=expression(g[r]), "K_f"=expression(K[f]), "r_f"=expression(r[f])))+
 ylim(-1,1)

B<-ggplot(duration, aes(x=param, y = original)) +
 geom_point(size = 4)+
 geom_errorbar(aes(ymax = maxCI, ymin = minCI))+
 ggtitle("B")+
 xlab("Parameters")+
 ylab (" ")+
 scale_x_discrete(labels = c("r_r"=expression(r[r]), "d_r"=expression(d[r]), "K_r"=expression(K[r]), "p_r"=expression(p[r]), "alpha"=expression(alpha),"beta_h" = expression(beta[b]), "beta_r"=expression(beta[r]),
 "b_h" = expression(b[h]),"d_h" = expression(d[h]), "d_f"=expression(d[f]), "gamma_h" = expression(gamma[b]), "gamma_r" = expression(gamma[r]), "g_h"=expression(g[h]), "g_r"=expression(g[r]), "K_f"=expression(K[f]), "r_f"=expression(r[f])))+
 ylim(-1,1)

## Bubonic SEIR with rat carrying capacity and resistance- Figure S6 (Panels C & D)

parameters <- c(r_r=0.014, K_r=499999, p_r=0.975, d_r=0.00055, beta_r = 0.09, alpha=3/500000, gamma_r = 1/5.15, g_r=0.1, r_f=0.0084, K_f=6, d_f=1/5, beta_h=0.19, sigma_h= 1/4, gamma_h=1/10, g_h=0.34, b_h=1/(25*365), d_h=1/(25*365)) #you can play with transmission and recovery rates here

par(mfrow=c(1,2))
plot(bSEIRrK$MaxInf~bSEIRrK$r_r, main= expression(paste("Effect of ", r[r], " on Size")), xlab=expression(r[r]), ylab="Outbreak Size")
plot(bSEIRrK$Thresh100~bSEIRrK$r_r, main= expression(paste("Effect of ", r[r], " on Duration")), xlab=expression(r[r]), ylab="Detectable Duration (days)")


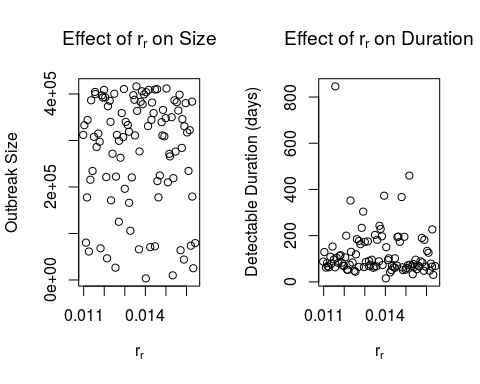


plot(bSEIRrK$MaxInf~bSEIRrK$K_r, main= expression(paste("Effect of ", K[r], " on Size")), xlab=expression(K[r]), ylab="Outbreak Size")
plot(bSEIRrK$Thresh100~bSEIRrK$K_r, main= expression(paste("Effect of ", K[r], " on Duration")), xlab=expression(K[r]), ylab="Detectable Duration (days)")


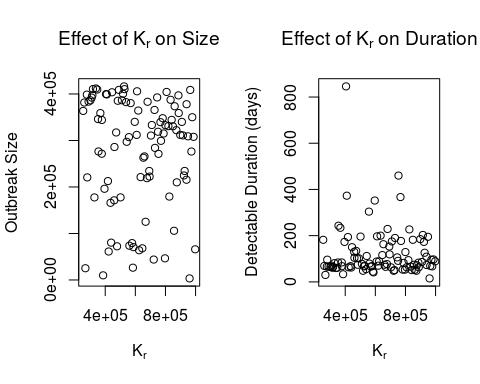


plot(bSEIRrK$MaxInf~bSEIRrK$p_r, main= expression(paste("Effect of ", p[r], " on Size")), xlab=expression(p[r]), ylab="Outbreak Size")
plot(bSEIRrK$Thresh100~bSEIRrK$p_r, main= expression(paste("Effect of ", p[r], " on Duration")), xlab=expression(p[r]), ylab="Detectable Duration (days)")


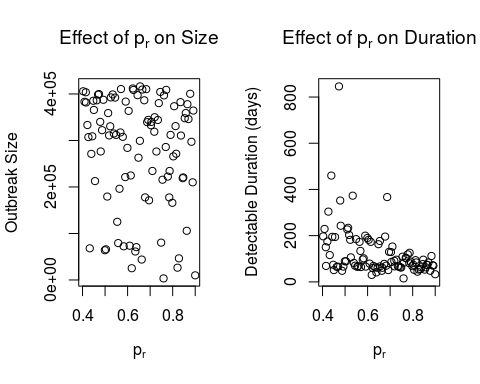


plot(bSEIRrK$MaxInf~bSEIRrK$d_r, main= expression(paste("Effect of ", d[r], " on Size")), xlab=expression(d[r]), ylab="Outbreak Size")
plot(bSEIRrK$Thresh100~bSEIRrK$d_r, main= expression(paste("Effect of ", d[r], " on Duration")), xlab=expression(d[r]), ylab="Detectable Duration (days)")


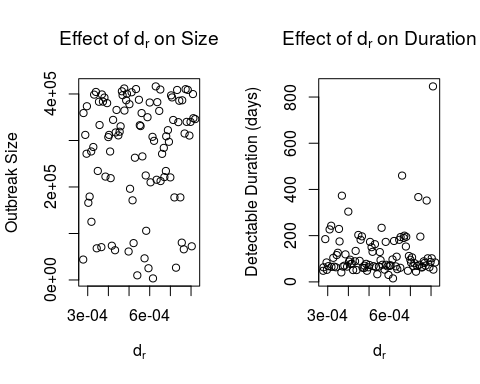


plot(bSEIRrK$MaxInf~bSEIRrK$beta_r, main= expression(paste("Effect of ", beta[r], " on Size")), xlab=expression(beta[r]), ylab="Outbreak Size")
plot(bSEIRrK$Thresh100~bSEIRrK$beta_r, main= expression(paste("Effect of ", beta[r], " on Duration")), xlab=expression(beta[r]), ylab="Detectable Duration (days)")


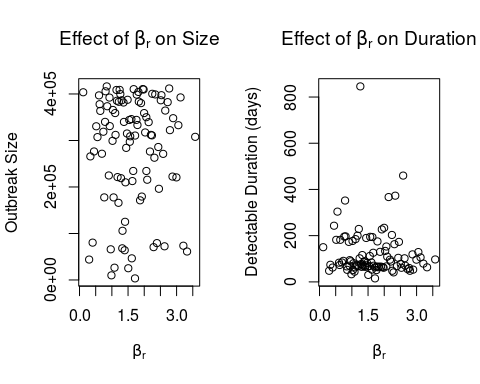


plot(bSEIRrK$MaxInf~bSEIRrK$alpha, main= expression(paste("Effect of ", alpha, " on Size")), xlab=expression(alpha), ylab="Outbreak Size")
plot(bSEIRrK$Thresh100~bSEIRrK$alpha, main= expression(paste("Effect of ", alpha, " on Duration")), xlab=expression(alpha), ylab="Detectable Duration (days)")


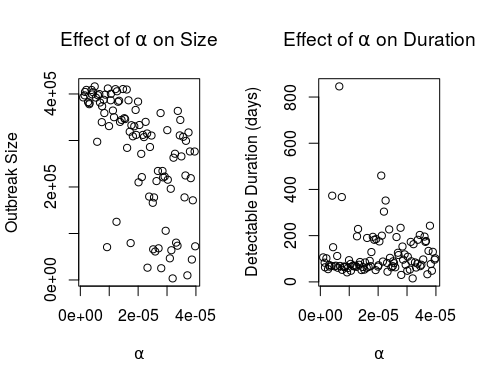


plot(bSEIRrK$MaxInf~bSEIRrK$gamma_r, main= expression(paste("Effect of ", gamma[r], " on Size")), xlab=expression(gamma[r]), ylab="Outbreak Size")
plot(bSEIRrK$Thresh100~bSEIRrK$gamma_r, main= expression(paste("Effect of ", gamma[r], " on Duration")), xlab=expression(gamma[r]), ylab="Detectable Duration (days)")


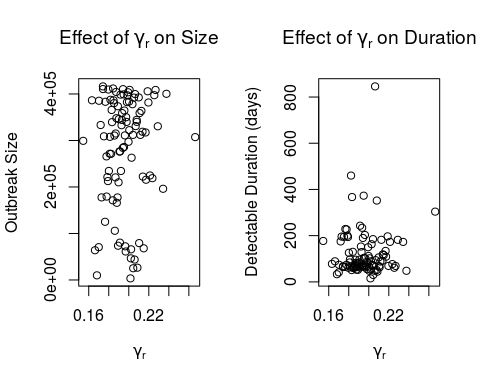


plot(bSEIRrK$MaxInf~bSEIRrK$g_r, main= expression(paste("Effect of ", g[r], " on Size")), xlab=expression(g[r]), ylab="Outbreak Size")
plot(bSEIRrK$Thresh100~bSEIRrK$g_r, main= expression(paste("Effect of ", g[r], " on Duration")), xlab=expression(g[r]), ylab="Detectable Duration (days)")


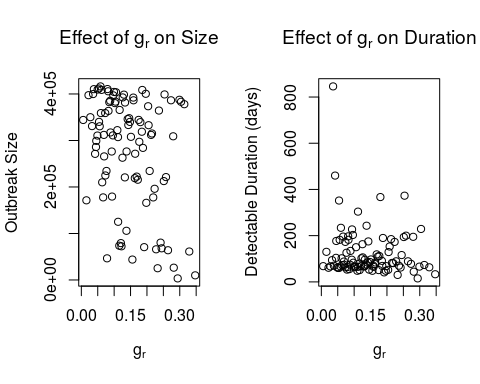


plot(bSEIRrK$MaxInf~bSEIRrK$r_f, main= expression(paste("Effect of ", r[f], " on Size")), xlab=expression(r[f]), ylab="Outbreak Size")
plot(bSEIRrK$Thresh100~bSEIRrK$r_f, main= expression(paste("Effect of ", r[f], " on Duration")), xlab=expression(r[f]), ylab="Detectable Duration (days)")


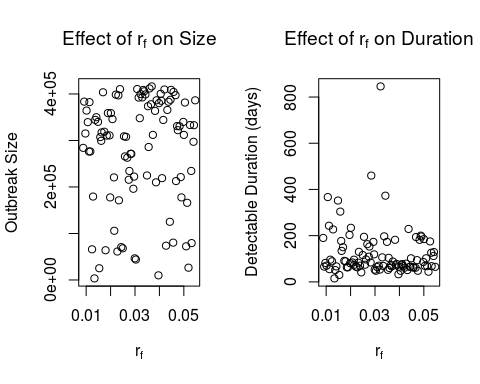


plot(bSEIRrK$MaxInf~bSEIRrK$K_f, main= expression(paste("Effect of ", K[f], " on Size")), xlab=expression(K[f]), ylab="Outbreak Size")
plot(bSEIRrK$Thresh100~bSEIRrK$K_f, main= expression(paste("Effect of ", K[f], " on Duration")), xlab=expression(K[f]), ylab="Detectable Duration (days)")


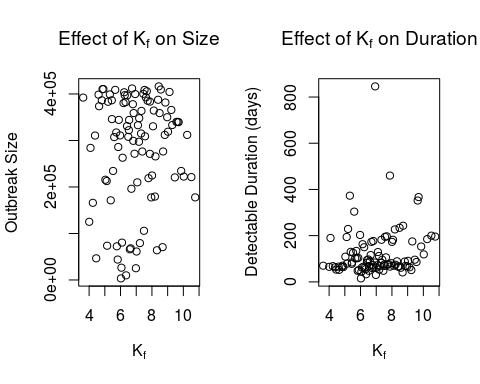


plot(bSEIRrK$MaxInf~bSEIRrK$d_f, main= expression(paste("Effect of ", d[f], " on Size")), xlab=expression(d[f]), ylab="Outbreak Size")
plot(bSEIRrK$Thresh100~bSEIRrK$d_f, main= expression(paste("Effect of ", d[f], " on Duration")), xlab=expression(d[f]), ylab="Detectable Duration (days)")


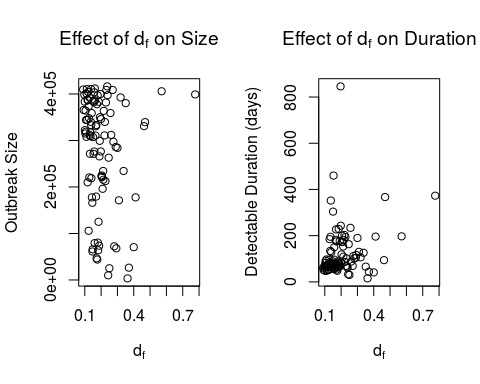


plot(bSEIRrK$MaxInf~bSEIRrK$beta_h, main= expression(paste("Effect of ", beta[b], " on Size")), xlab=expression(beta[b]), ylab="Outbreak Size")
plot(bSEIRrK$Thresh100~bSEIRrK$beta_h, main= expression(paste("Effect of ", beta[b], " on Duration")), xlab=expression(beta[b]), ylab="Detectable Duration (days)")


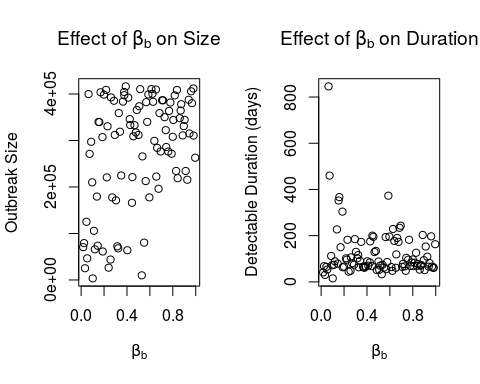


plot(bSEIRrK$MaxInf~bSEIRrK$beta_h, main= expression(paste("Effect of ", beta[b], " on Size")), xlab=expression(sigma[b]), ylab="Outbreak Size")
plot(bSEIRrK$Thresh100~bSEIRrK$beta_h, main= expression(paste("Effect of ", beta[b], " on Duration")), xlab=expression(sigma[b]), ylab="Detectable Duration (days)")


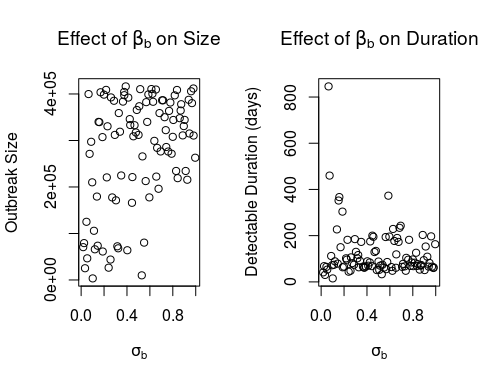


plot(bSEIRrK$MaxInf~bSEIRrK$gamma_h, main= expression(paste("Effect of ", gamma[b], " on Size")), xlab=expression(gamma[b]), ylab="Outbreak Size")
plot(bSEIRrK$Thresh100~bSEIRrK$gamma_h, main= expression(paste("Effect of ", gamma[b], " on Duration")), xlab=expression(gamma[b]), ylab="Detectable Duration (days)")


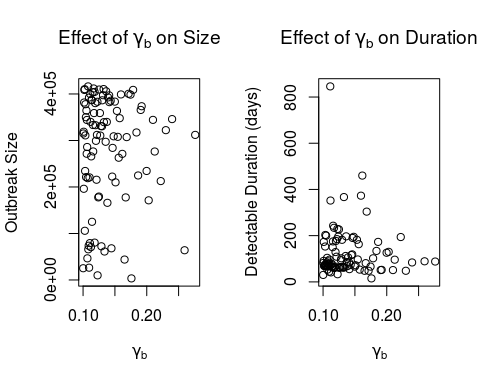


plot(bSEIRrK$MaxInf~bSEIRrK$g_h, main= expression(paste("Effect of ", g[h], " on Size")), xlab=expression(g[h]), ylab="Outbreak Size")
plot(bSEIRrK$Thresh100~bSEIRrK$g_h, main= expression(paste("Effect of ", g[h], " on Duration")), xlab=expression(g[h]), ylab="Detectable Duration (days)")


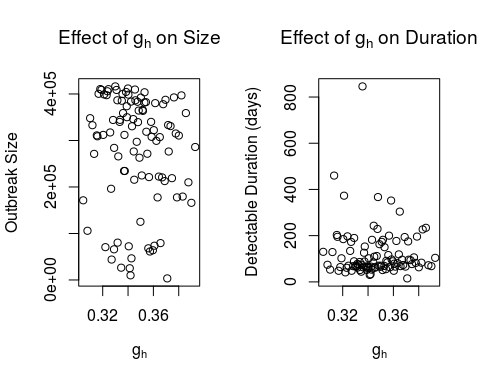


plot(bSEIRrK$MaxInf~bSEIRrK$b_h, main= expression(paste("Effect of ", b[h], " on Size")), xlab=expression(b[h]), ylab="Outbreak Size")
plot(bSEIRrK$Thresh100~bSEIRrK$b_h, main= expression(paste("Effect of ", b[h], " on Duration")), xlab=expression(r[r]), ylab="Detectable Duration (days)")


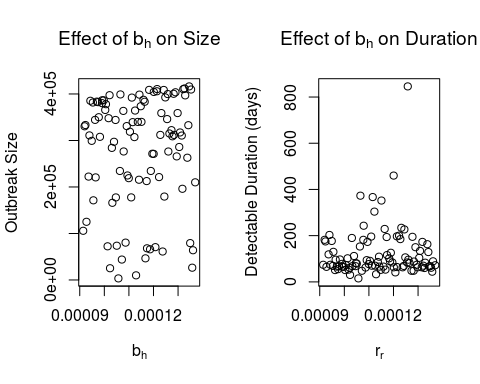


plot(bSEIRrK$MaxInf~bSEIRrK$d_h, main= expression(paste("Effect of ", d[h], " on Size")), xlab=expression(d[h]), ylab="Outbreak Size")
plot(bSEIRrK$Thresh100~bSEIRrK$d_h, main= expression(paste("Effect of ", d[h], " on Duration")), xlab=expression(d[h]), ylab="Detectable Duration (days)")


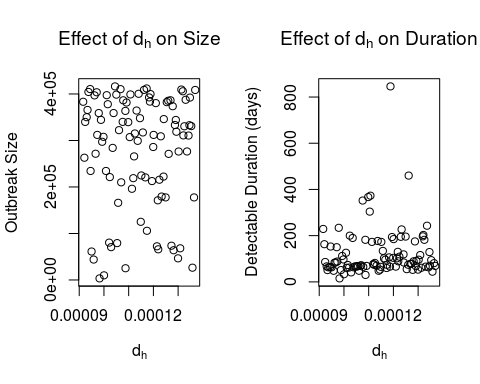


par(mfrow=c(1,2))
boxplot(bSEIRrK$MaxInf, main= "Outbreak Size", ylab= "Number of Dead Humans", ylim=c(0,500000))
boxplot(bSEIRrK$Thresh100, main= "Outbreak Duration", ylab="Time (Days)")


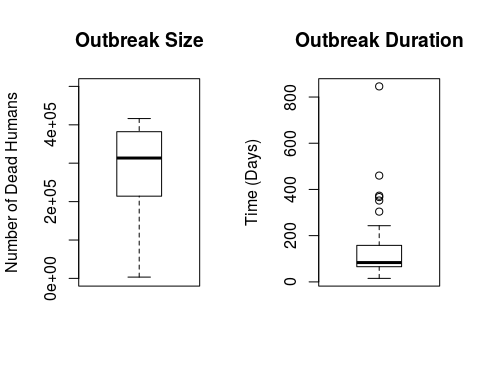


bonferroni.alpha <- 0.05/length(parameters)
prcc_size <- pcc(bSEIRrK[,1:length(parameters)], bSEIRrK$MaxInf, nboot = niter, rank=TRUE, conf=1-bonferroni.alpha)
prcc_duration <- pcc(bSEIRrK[,1:length(parameters)], bSEIRrK$Thresh100, nboot = niter, rank=TRUE, conf=1-bonferroni.alpha)

#plot correlation coefficients and confidence intervals for epidemic size and duration
size<-prcc_size$PRCC
size$param<-rownames(size)
colnames(size)[4:5] <- c("maxCI", "minCI")
size$maxCI[which(size$maxCI>1)]<-1
size$maxCI[which(size$maxCI< -1)]<- -1
size$minCI[which(size$minCI>1)]<-1
size$minCI[which(size$minCI< -1)]<- -1

duration<-prcc_duration$PRCC
duration$param<-rownames(duration)
colnames(duration)[4:5] <- c("maxCI", "minCI")
duration$maxCI[which(duration$maxCI>1)]<-1
duration$maxCI[which(duration$maxCI< -1)]<- -1
duration$minCI[which(duration$minCI>1)]<-1
duration$minCI[which(duration$minCI< -1)]<- -1


C<- ggplot(size, aes(x=param, y = original)) +
 geom_point(size = 4)+
 geom_errorbar(aes(ymax = maxCI, ymin = minCI))+
 ggtitle("C")+
 xlab("Parameters")+
 ylab ("Partial Rank Correlation Coefficients")+
 scale_x_discrete(labels = c("r_r"=expression(r[r]), "d_r"=expression(d[r]), "K_r"=expression(K[r]), "p_r"=expression(p[r]), "alpha"=expression(alpha),"beta_h" = expression(beta[b]), "beta_r"=expression(beta[r]), "sigma_h"=expression(sigma[b]),
 "b_h" = expression(b[h]),"d_h" = expression(d[h]), "d_f"=expression(d[f]), "gamma_h" = expression(gamma[b]), "gamma_r" = expression(gamma[r]), "g_h"=expression(g[h]), "g_r"=expression(g[r]), "K_f"=expression(K[f]), "r_f"=expression(r[f])))+
 ylim(-1,1)

D<-ggplot(duration, aes(x=param, y = original)) +
 geom_point(size = 4)+
 geom_errorbar(aes(ymax = maxCI, ymin = minCI))+
 ggtitle("D")+
 xlab("Parameters")+
 ylab (" ")+
 scale_x_discrete(labels = c("r_r"=expression(r[r]), "d_r"=expression(d[r]), "K_r"=expression(K[r]), "p_r"=expression(p[r]), "alpha"=expression(alpha),"beta_h" = expression(beta[b]), "beta_r"=expression(beta[r]), "sigma_h"=expression(sigma[b]),
 "b_h" = expression(b[h]),"d_h" = expression(d[h]), "d_f"=expression(d[f]), "gamma_h" = expression(gamma[b]), "gamma_r" = expression(gamma[r]), "g_h"=expression(g[h]), "g_r"=expression(g[r]), "K_f"=expression(K[f]), "r_f"=expression(r[f])))+
 ylim(-1,1)

tiff("FigS6.tiff", height =22.23 , width =19.05, units = "cm", compression = "lzw", res = 600)
multiplot(A, B, C, D, cols=2)
dev.off()

## png
## 2

## Bubonic/Pneumonic SEIR- Figure S7

parameters <- c(beta_r = 0.09, alpha=3/500000, gamma_r = 1/5.15, g_r=0.1, r_f=0.0084, K_f=6, d_f=1/5, beta_b=0.19, beta_p = 0.45, sigma_b= 1/6, sigma_p=1/4.3, gamma_b=1/10, gamma_p=1/2.5, p=0.2, g_h=0.34, b_h=1/(25*365), d_h=1/(25*365)) #you can play with transmission and recovery rates here

par(mfrow=c(1,2))
plot(bpSEIR$MaxInf~bpSEIR$beta_r, main= expression(paste("Effect of ", beta[r], " on Size")), xlab=expression(beta[r]), ylab="Outbreak Size")
plot(bpSEIR$Thresh100~bpSEIR$beta_r, main= expression(paste("Effect of ", beta[r], " on Duration")), xlab=expression(beta[r]), ylab="Detectable Duration (days)")


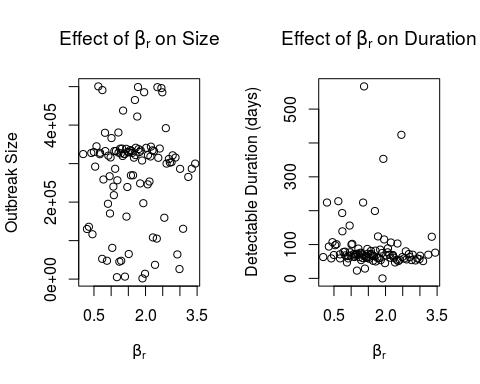


plot(bpSEIR$MaxInf~bpSEIR$alpha, main= expression(paste("Effect of ", alpha, " on Size")), xlab=expression(alpha), ylab="Outbreak Size")
plot(bpSEIR$Thresh100~bpSEIR$alpha, main= expression(paste("Effect of ", alpha, " on Duration")), xlab=expression(alpha), ylab="Detectable Duration (days)")


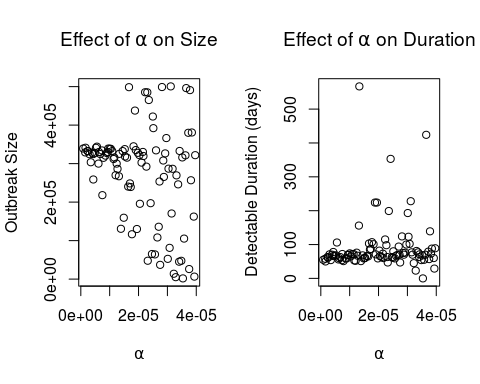


plot(bpSEIR$MaxInf~bpSEIR$gamma_r, main= expression(paste("Effect of ", gamma[r], " on Size")), xlab=expression(gamma[r]), ylab="Outbreak Size")
plot(bpSEIR$Thresh100~bpSEIR$gamma_r, main= expression(paste("Effect of ", gamma[r], " on Duration")), xlab=expression(gamma[r]), ylab="Detectable Duration (days)")


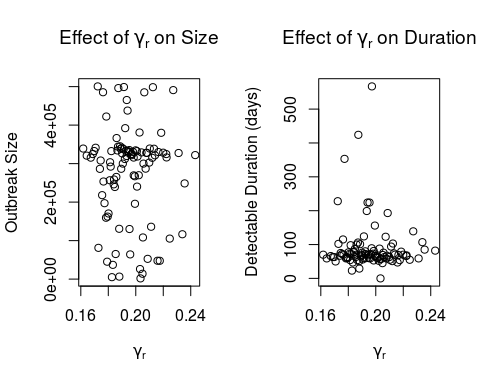


plot(bpSEIR$MaxInf~bpSEIR$g_r, main= expression(paste("Effect of ", g[r], " on Size")), xlab=expression(g[r]), ylab="Outbreak Size")
plot(bpSEIR$Thresh100~bpSEIR$g_r, main= expression(paste("Effect of ", g[r], " on Duration")), xlab=expression(g[r]), ylab="Detectable Duration (days)")


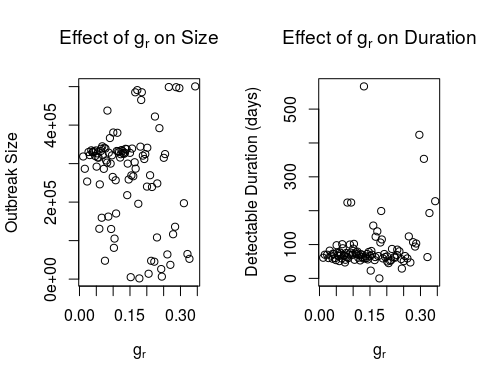


plot(bpSEIR$MaxInf~bpSEIR$r_f, main= expression(paste("Effect of ", r[f], " on Size")), xlab=expression(r[f]), ylab="Outbreak Size")
plot(bpSEIR$Thresh100~bpSEIR$r_f, main= expression(paste("Effect of ", r[f], " on Duration")), xlab=expression(r[f]), ylab="Detectable Duration (days)")


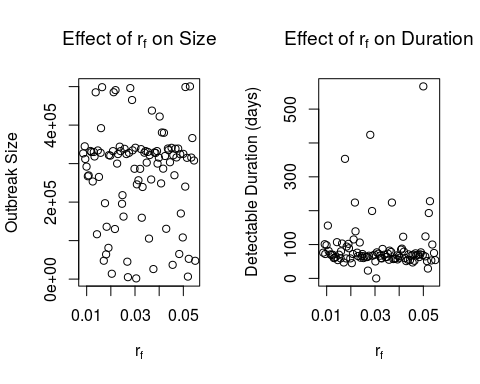


plot(bpSEIR$MaxInf~bpSEIR$K_f, main= expression(paste("Effect of ", K[f], " on Size")), xlab=expression(K[f]), ylab="Outbreak Size")
plot(bpSEIR$Thresh100~bpSEIR$K_f, main= expression(paste("Effect of ", K[f], " on Duration")), xlab=expression(K[f]), ylab="Detectable Duration (days)")


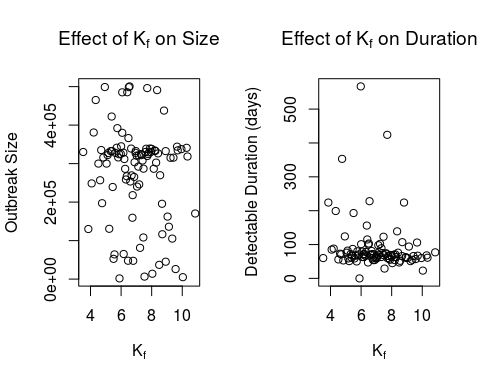


plot(bpSEIR$MaxInf~bpSEIR$d_f, main= expression(paste("Effect of ", d[f], " on Size")), xlab=expression(d[f]), ylab="Outbreak Size")
plot(bpSEIR$Thresh100~bpSEIR$d_f, main= expression(paste("Effect of ", d[f], " on Duration")), xlab=expression(d[f]), ylab="Detectable Duration (days)")


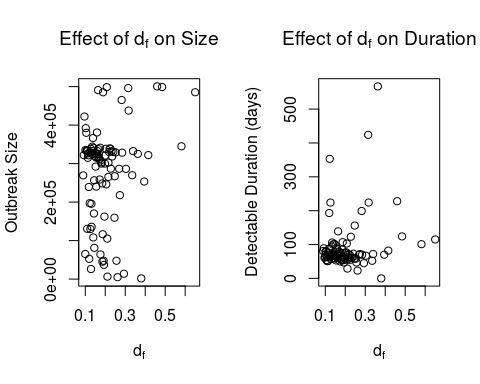


plot(bpSEIR$MaxInf~bpSEIR$beta_b, main= expression(paste("Effect of ", beta[b], " on Size")), xlab=expression(beta[b]), ylab="Outbreak Size")
plot(bpSEIR$Thresh100~bpSEIR$beta_b, main= expression(paste("Effect of ", beta[b], " on Duration")), xlab=expression(beta[b]), ylab="Detectable Duration (days)")


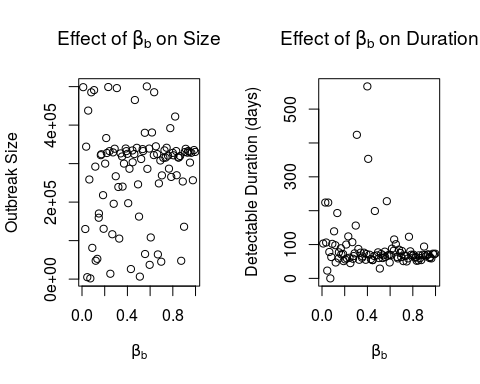


plot(bpSEIR$MaxInf~bpSEIR$sigma_b, main= expression(paste("Effect of ", sigma[b], " on Size")), xlab=expression(sigma[b]), ylab="Outbreak Size")
plot(bpSEIR$Thresh100~bpSEIR$sigma_b, main= expression(paste("Effect of ", sigma[b], " on Duration")), xlab=expression(sigma[b]), ylab="Detectable Duration (days)")


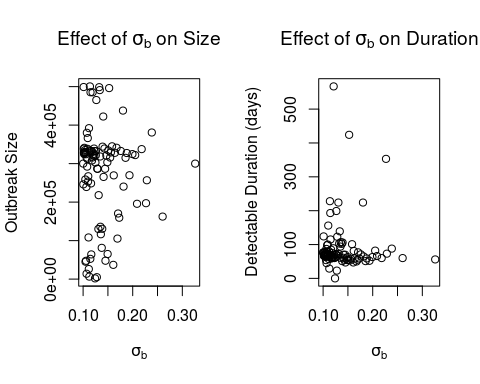


plot(bpSEIR$MaxInf~bpSEIR$gamma_b, main= expression(paste("Effect of ", gamma[b], " on Size")), xlab=expression(gamma[b]), ylab="Outbreak Size")
plot(bpSEIR$Thresh100~bpSEIR$gamma_b, main= expression(paste("Effect of ", gamma[b], " on Duration")), xlab=expression(gamma[b]), ylab="Detectable Duration (days)")


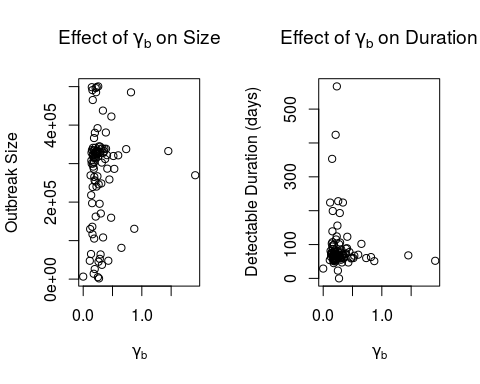


plot(bpSEIR$MaxInf~bpSEIR$beta_p, main= expression(paste("Effect of ", beta[p], " on Size")), xlab=expression(beta[p]), ylab="Outbreak Size")
plot(bpSEIR$Thresh100~bpSEIR$beta_p, main= expression(paste("Effect of ", beta[p], " on Duration")), xlab=expression(beta[p]), ylab="Detectable Duration (days)")


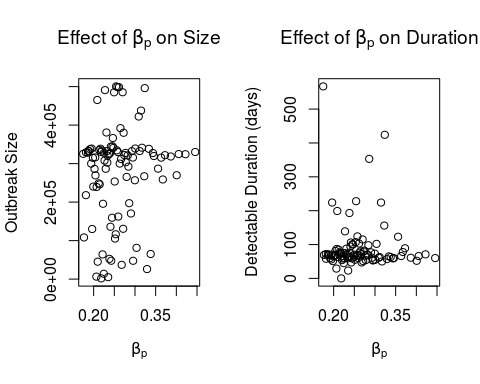


plot(bpSEIR$MaxInf~bpSEIR$sigma_p, main= expression(paste("Effect of ", sigma[p], " on Size")), xlab=expression(sigma[p]), ylab="Outbreak Size")
plot(bpSEIR$Thresh100~bpSEIR$sigma_p, main= expression(paste("Effect of ", sigma[p], " on Duration")), xlab=expression(sigma[p]), ylab="Detectable Duration (days)")


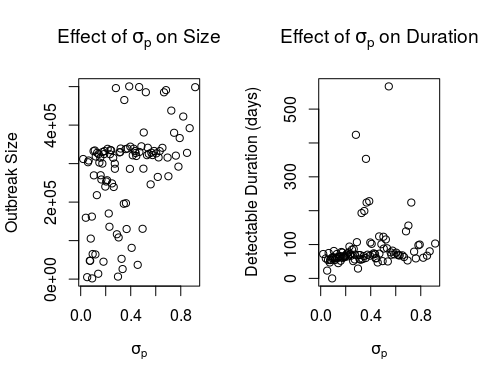


plot(bpSEIR$MaxInf~bpSEIR$gamma_p, main= expression(paste("Effect of ", gamma[p], " on Size")), xlab=expression(gamma[p]), ylab="Outbreak Size")
plot(bpSEIR$Thresh100~bpSEIR$gamma_p, main= expression(paste("Effect of ", gamma[p], " on Duration")), xlab=expression(gamma[p]), ylab="Detectable Duration (days)")


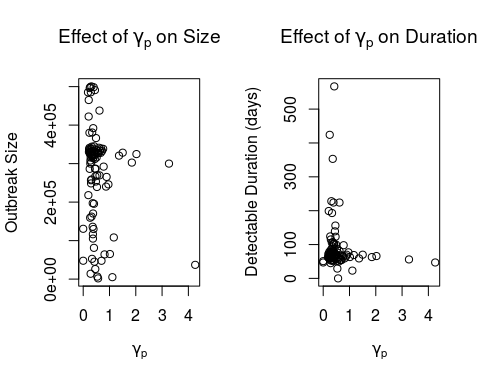


plot(bpSEIR$MaxInf~bpSEIR$g_h, main= expression(paste("Effect of ", g[h], " on Size")), xlab=expression(g[h]), ylab="Outbreak Size")
plot(bpSEIR$Thresh100~bpSEIR$g_h, main= expression(paste("Effect of ", g[h], " on Duration")), xlab=expression(g[h]), ylab="Detectable Duration (days)")


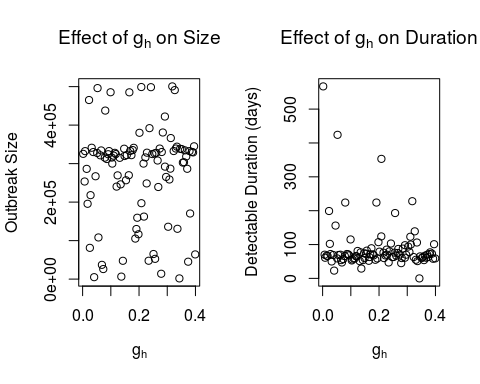


plot(bpSEIR$MaxInf~bpSEIR$p, main= expression(paste("Effect of ", p, " on Size")), xlab="p", ylab="Outbreak Size")
plot(bpSEIR$Thresh100~bpSEIR$p, main= expression(paste("Effect of ", p, " on Duration")), xlab="p", ylab="Detectable Duration (days)")


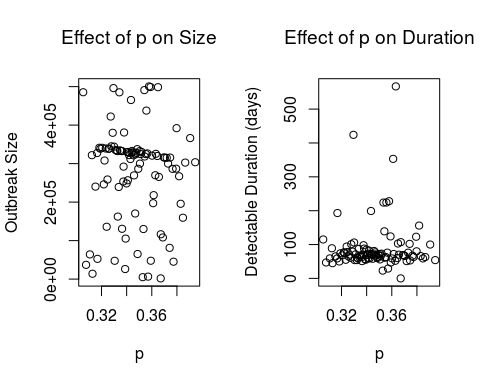


plot(bpSEIR$MaxInf~bpSEIR$b_h, main= expression(paste("Effect of ", b[h], " on Size")), xlab=expression(b[h]), ylab="Outbreak Size")
plot(bpSEIR$Thresh100~bpSEIR$b_h, main= expression(paste("Effect of ", b[h], " on Duration")), xlab=expression(b[h]), ylab="Detectable Duration (days)")


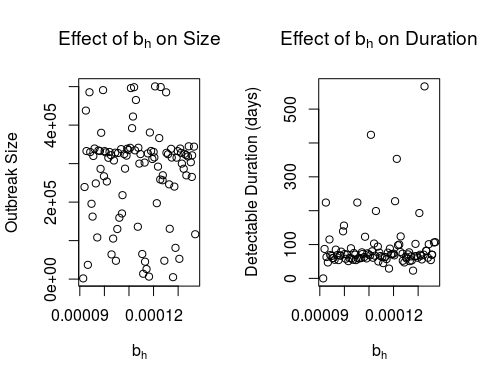


plot(bpSEIR$MaxInf~bpSEIR$d_h, main= expression(paste("Effect of ", d[h], " on Size")), xlab=expression(d[h]), ylab="Outbreak Size")
plot(bpSEIR$Thresh100~bpSEIR$d_h, main= expression(paste("Effect of ", d[h], " on Duration")), xlab=expression(d[h]), ylab="Detectable Duration (days)")


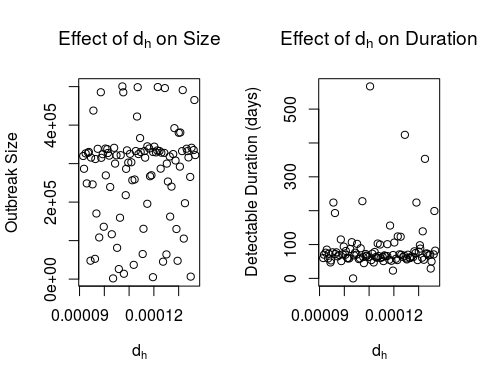


par(mfrow=c(1,2))
boxplot(bpSEIR$MaxInf, main= "Outbreak Size", ylab= "Number of Dead Humans", ylim=c(0,500000))
boxplot(bpSEIR$Thresh100, main= "Outbreak Duration", ylab="Time (Days)")


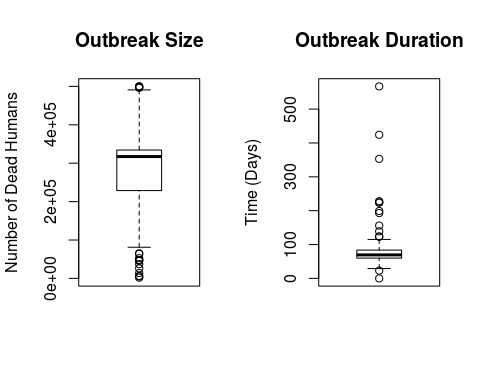


bonferroni.alpha <- 0.05/length(parameters)
prcc_size <- pcc(bpSEIR[,1:length(parameters)], bpSEIR[,length(parameters)+1], nboot = niter, rank=TRUE, conf=1-bonferroni.alpha)
prcc_duration <- pcc(bpSEIR[,1:length(parameters)], bpSEIR[,length(parameters)+2], nboot = niter, rank=TRUE, conf=1-bonferroni.alpha)

#plot correlation coefficients and confidence intervals for epidemic size and duration
size<-prcc_size$PRCC
size$param<-rownames(size)
colnames(size)[4:5] <- c("maxCI", "minCI")
size$maxCI[which(size$maxCI>1)]<-1
size$maxCI[which(size$maxCI< -1)]<- -1
size$minCI[which(size$minCI>1)]<-1
size$minCI[which(size$minCI< -1)]<- -1

duration<-prcc_duration$PRCC
duration$param<-rownames(duration)
colnames(duration)[4:5] <- c("maxCI", "minCI")
duration$maxCI[which(duration$maxCI>1)]<-1
duration$maxCI[which(duration$maxCI< -1)]<- -1
duration$minCI[which(duration$minCI>1)]<-1
duration$minCI[which(duration$minCI< -1)]<- -1


A<- ggplot(size, aes(x=param, y = original)) +
 geom_point(size = 4)+
 geom_errorbar(aes(ymax = maxCI, ymin = minCI))+
 ggtitle("A")+
 xlab("Parameters")+
 ylab ("Partial Rank Correlation Coefficients")+
 scale_x_discrete(labels = c("alpha"=expression(alpha),"beta_b" = expression(beta[b]), "beta_p" = expression(beta[p]),"beta_r"=expression(beta[r]),
 "b_h" = expression(b[h]),"d_h" = expression(d[h]), "d_f"=expression(d[f]), "sigma_b"=expression(sigma[b]), "gamma_b" = expression(gamma[b]), "sigma_p"=expression(sigma[p]), "gamma_p"=expression(gamma[p]), "gamma_r" = expression(gamma[r]), "g_h"=expression(g[h]), "g_r"=expression(g[r]), "K_f"=expression(K[f]), "r_f"=expression(r[f])))+
 ylim(-1 ,1)


B<-ggplot(duration, aes(x=param, y = original)) +
 geom_point(size = 4)+
 geom_errorbar(aes(ymax = maxCI, ymin = minCI))+
 ggtitle("B")+
 xlab("Parameters")+
 ylab (" ")+
 scale_x_discrete(labels = c("alpha"=expression(alpha),"beta_b" = expression(beta[b]), "beta_p" = expression(beta[p]),"beta_r"=expression(beta[r]),
 "b_h" = expression(b[h]),"d_h" = expression(d[h]), "d_f"=expression(d[f]), "sigma_b"=expression(sigma[b]), "gamma_b" = expression(gamma[b]), "sigma_p"=expression(sigma[p]), "gamma_p"=expression(gamma[p]), "gamma_r" = expression(gamma[r]), "g_h"=expression(g[h]), "g_r"=expression(g[r]), "K_f"=expression(K[f]), "r_f"=expression(r[f])))+
 ylim(-1 ,1)

multiplot(A, B, cols=2)


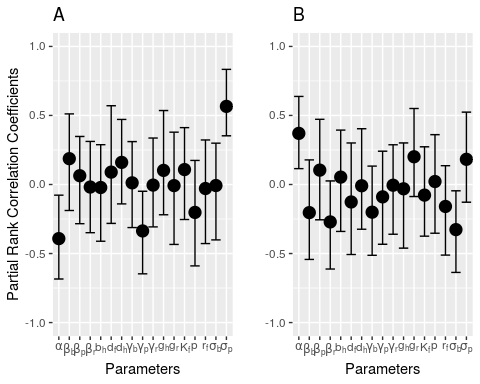


tiff("FigS7.tiff", height =10 , width =19.05, units = "cm", compression = "lzw", res = 600)
multiplot(A, B, cols=2)
dev.off()

## png
## 2
